# Mapping 2-Year Psychiatric and Neurologic Risks After Infections Across Body Systems and Age Groups

**DOI:** 10.1001/jamapsychiatry.2026.1904

**Published:** 2026-07-15

**Authors:** Maxime Taquet, Patrick Oliver, Ahmad Mezher, Campbell Robertson, Carla Handford, Thomas A. Pollak, Edoardo G. Ostinelli, Orestis Efthimiou, Andrea Cipriani, Paul J. Harrison

**Affiliations:** 1 Department of Psychiatry, University of Oxford, Oxford, United Kingdom; 2 Oxford Health NHS Foundation Trust, Oxford, United Kingdom; 3 Medical Sciences Division, University of Oxford, Oxford, United Kingdom; 4 Department of Psychosis Studies, Institute of Psychiatry, Psychology and Neuroscience, King’s College London, London, United Kingdom; 5 Institute of Primary Health Care, University of Bern, Bern, Switzerland; 6 Institute of Social and Preventive Medicine, University of Bern, Bern, Switzerland

## Abstract

**Importance:**

Infections are associated with postacute psychiatric and neurologic disorders, but whether risks generalize across infections affecting different body systems and how they vary by age remain unclear.

**Objective:**

To map risks of 14 psychiatric and neurologic disorders among patients hospitalized with infections across 10 body systems and 4 age groups.

**Design, Setting, and Participants:**

This was a retrospective multicohort study using propensity score matching of patients hospitalized between 2014 and 2018, with a 2-year follow-up. Patient data were taken from 62 health care organizations within the TriNetX US Collaborative Network. Included were cohorts of individuals hospitalized with an infection compared with each other, with individuals hospitalized for noninfectious causes, and with the general population. Data were analyzed from May 2024 to March 2026.

**Exposure:**

Hospitalization with an infection.

**Main Outcomes and Measures:**

Thirteen neurologic or psychiatric disorders (plus a composite category of cognitive deficits) diagnosed between 1 month and 2 years after hospital admission. Measures included the ratio of restricted mean time lost (RMTL), absolute risk difference, and infection specificity per outcome via network meta-analysis.

**Results:**

Among 1 062 722 matched pairs of individuals (mean [SD] age, 48.4 [23.6] years; 547 328 female [51.5%]), infections were associated with a greater risk of most disorders. When compared with hospitalization for other causes, RMTL ratios exceeded 1 in 116 of 140 infection-disorder tests; the highest relative risk was observed for encephalitis (eg, among cardiac infections, median RMTL ratio, 6.54; 95% CI, 3.78-11.31; *P* = 2.0 × 10^−11^), and the highest absolute risk difference was for cognitive deficits (eg, among cardiac infections, risk difference, 12.37%; 19.08% [95% CI, 18.51-19.65] vs 6.71% [95% CI, 6.36-7.07]). Similar patterns were observed (though with higher RMTL ratios) when patients hospitalized with infections were compared with the general population. Although children also faced increased risks of psychiatric and neurologic disorders after infections, absolute risk differences were significantly lower than those in adults. In 45 head-to-head comparisons (median [IQR] sample, 30 766 [11 856-104 336]), infectious encephalitides were the leading risk factor for 7 of 14 disorders and among the top 3 for 12 of 14 disorders. Other infectious risk factors were disorder specific.

**Conclusions and Relevance:**

Results of this multicohort study suggest that patients hospitalized with infections across all body systems had an associated increase in psychiatric and neurologic diagnoses over the subsequent 2 years, particularly in adults. Infectious encephalitides carried the greatest associated excess risk. Taken together with the existing literature, these findings identify infections as important risk factors for brain health conditions.

## Key Points

**Question:**

Do risks of psychiatric and neurologic disorders increase in the 2 years after infections, and how do they vary by body system affected and age?

**Findings:**

In this multicohort study of more than 1 million infections during which patients were hospitalized, infections were associated with significantly higher risk for most outcomes across age groups, though less so in children. Infectious encephalitides were a leading infectious risk factor for most disorders, whereas other infections were more disorder specific.

**Meaning:**

These findings suggest that infections are important risk factors for psychiatric and neurologic disorders, can guide research into underlying mechanisms, and may help improve future pandemic preparedness.

## Teaser

This cohort study investigates the association between infection and risks of psychiatric and neurologic disorders and how risks vary by body system affected and age.

## Introduction

Infections have long been considered risk factors for psychiatric and neurologic conditions. Seminal nationwide studies^1,2,3^ from Denmark have found that infections are associated with an increased risk of psychiatric disorders. A Swedish study^4^ showed an association between viral infections of the central nervous system in childhood and risk of psychosis, with findings subsequently extended to other psychiatric disorders and cognitive impairment.^5^ Another large study^6^ found that hospitalized infections are associated with an increased risk of dementia. Likewise, infections have been associated with increased risks of neurologic conditions including strokes after respiratory and urinary infections,^7^ multiple sclerosis after infectious mononucleosis,^8^ seizures after encephalitis and meningitis,^9^ and Parkinson disease after respiratory and gastrointestinal infections.^10^ The COVID-19 pandemic further underscored this burden, with large electronic health record (EHR) studies^11,12,13^ showing increased risks of psychiatric and neurologic diagnoses after SARS-CoV-2 infection, partly shared with other infections among hospitalized individuals.^14^

Despite growing evidence implicating infections in the onset of psychiatric and neurologic disorders, the literature is fragmented and important gaps remain. Large-scale studies^1,2,3^ either aggregate heterogeneous infections as a single exposure (ie, any infection) or focus on a single pathogen (notably SARS-CoV-2^11,12,13^) or body system.^4^ To our knowledge, no study has assessed the risk of a wide range of psychiatric and neurologic disorders across infections affecting different body systems or has stratified risks by age in the same analytic setting, limiting assessment of moderation of risks by age. A risk map across body systems, disorders, and age groups is, therefore, needed to identify shared and distinct patterns of association.

The present study addresses these gaps by comparing individuals hospitalized with infections across 10 body systems with each other, with hospitalizations for noninfectious causes and with the general population, assessing age-stratified risks of 14 psychiatric and neurologic disorders during the 1-month to 2-year period after hospital admission.

## Methods

### Study Design and Data Collection

We used the TriNetX US Collaborative Network, a federated EHR database of over 100 million patients (both insured and uninsured) across 62 health care organizations in the US. Data include sociodemographics, *International Statistical Classification of Diseases, Tenth Revision, Clinical Modification *(*ICD-10-CM*) diagnostic codes, medications, and health care visits from primary and specialist health care centers. Data deidentification is formally attested as per section §164.514(b)(1) of the Health Insurance Portability and Accountability Act Privacy Rule. As we used anonymized routinely collected data, no participant consent or ethical approval was required. Additional details about TriNetX are presented in eMethods 1 in Supplement 1. Our study followed the Strengthening the Reporting of Observational Studies in Epidemiology (STROBE) reporting guidelines.

### Cohorts and Exposures

We identified individuals hospitalized between January 1, 2014, and December 31, 2018 (chosen to predate the COVID-19 pandemic and balance sample size with consistent diagnostic practice).

Infection cohorts comprised individuals hospitalized with an infection in 1 of 10 body systems coded on hospital admission: infectious encephalitides, infectious hepatitides, meningitides, bone, cardiac, gastrointestinal, respiratory tract infection (RTI), skin, sexually transmitted infection (STI), and urinary tract infection (UTI). Where relevant, we only included acute forms of infections (eg, acute but not chronic viral hepatitides). One exception is HIV, whose acute and chronic forms are undifferentiated in *ICD-10*.

The cohorts without infection comprised (1) hospitalized individuals and (2) the general population who made at least 1 outpatient visit (including primary care visit) during the study period. Those who had a recorded diagnosis of infection, prescription of antimicrobials, serological evidence of infection, or fever of unknown origin up to 1 month before their hospitalization or outpatient visit were excluded from these cohorts without infection to ensure that their visit was not infection related.

All cohorts were stratified by age as follows: 18 years and younger (children), 19 to 44 years (young adults), 45 to 64 years (middle-aged adults), and 65 years and older (older adults).

A complete list of *ICD-10* and medication codes used can be found in the eMethods 2 in Supplement 1.

### Covariates

For each pairwise comparison, we used propensity score matching to adjust for 92 potential confounders: sociodemographic factors (age, sex, race, ethnicity, marital status, socioeconomic deprivation), comorbidities across body systems, prior vaccinations (eg, against influenza; tetanus, diphtheria, and pertussis; pneumococcus; hepatitis A; hepatitis B), and prior or concurrent antidepressant or antipsychotic medication use. More details, including codes, are provided in eMethods 3 in Supplement 1. Race categories included American Indian or Alaska Native, Asian, Black or African American, Native Hawaiian or Other Pacific Islander, White, other race, and unknown race. Ethnicity categories included Hispanic or Latino, not Hispanic or Latino, and unknown ethnicity. Race and ethnicity were recorded in the patients’ health records and were included as covariates because of their known associations with health outcomes, health inequalities, and health care use.

### Outcomes

We investigated the risk of 14 psychiatric and neurologic disorders in the 31 to 730 days after hospital admission, excluding the first month to focus on postacute onset and limit reverse causation. The outcomes were anxiety, mood, and psychotic disorders; insomnia; cognitive deficits (a composite measure used in previous studies^11,15,16^); dementia; epilepsy and seizures; encephalitis (both infectious and noninfectious, unlike infectious encephalitides used as an exposure); intracranial hemorrhage; ischemic stroke; parkinsonism; multiple sclerosis; nerve, nerve root, and plexus disorders; and neuromuscular disease. As in our previous studies,^11,16,17^ for outcomes that were chronic or recurrent (eg, dementia, mood disorder), we excluded those with the disorder before the index event and report the incidence of first diagnosis (because recurrent diagnostic codes often capture a preexisting disorder). For outcomes that do not tend to recur after they have resolved (eg, nerve, nerve root, and plexus disorder), we estimated the risk of any diagnosis (because recurrent codes more likely capture new events, eg, nerve disorder affecting another body part). eMethods 4 in Supplement 1 provides a list of *ICD-10* codes.

### Statistical Analysis

We used 1:1 greedy nearest neighbor propensity score matching for covariates. The Kaplan-Meier estimator was used to estimate the cumulative incidence (risk) of each disorder. Given the long follow-up, we did not assume proportional hazards. Our primary estimand was the ratio of restricted mean time lost (RMTL),^18^ which provides a clinically meaningful measure of how much more time, on average, an individual lived with the disorder during the follow-up. Absolute risk differences were calculated at 2 years. We also estimated relative risks (ratios of cumulative incidences at 2 years) for ease of interpretation.

To assess moderation by age, we estimated inverse variance–weighted regression models where the log-RMTL ratio and absolute risk differences were dependent variables, and age group, infection, disorder, age × infection, and age × disorder interactions were independent variables. We report marginal means for the overall age moderation and for the age moderation within specific infections and disorders.

For each outcome, we used network meta-analysis methods to rank infections by their association with the outcome. Using the log-RMTL ratios as inputs, the network meta-analysis model provided P scores (ranging from 0-1) for each infection, representing the certainty that the disorder is more likely after that infection compared with other infections.^19^ This was performed within each age group and in the pooled cohort. Inconsistency was assessed using the between-design Q statistic.^20^

To assess if disorders shared infectious risk factors, we computed pairwise Pearson correlations between the risk factor profiles (the list of 45 pairwise RMTL ratios comparing the risk of the disorder between 2 infections). We represented correlations in a matrix with *P* values (Bonferroni corrected for 91 = 14 × 13/2 comparisons). Hierarchical clustering with dynamic dendrogram pruning was used to identify groups of disorders with similar risk factors.

Unrecorded health events were assumed not to have occurred. Missing sociodemographic data (sex, race, ethnicity, marital status) were assigned as *unknown* and included as a category in the propensity score matching (as a result, matched cohorts had similar proportions of unknown characteristics).

All statistical analyses were conducted from May 2024 to March 2026 using R, version 4.2.1 (R Project for Statistical Computing), unless otherwise stated. Statistical significance was set at 2-sided *P* < .05. To compare the 10 cohorts with infection against the controls without infection, Bonferroni correction was applied for the 10 comparisons made per disorder. For age moderation analyses, Benjamini-Hochberg adjustment was applied within each family of 4 comparisons. Further details about statistical analyses can be found in eMethods 5 in the Supplement.

## Results

### Cohort Descriptions

We conducted 65 pairwise studies with 1 062 722 matched pairs (mean [SD] age, 48.4 [23.6] years; 547 328 female [51.5%]; 494 665 male [46.5%]; 20 729 other or unknown [2.0%]) including 10 comparisons between individuals hospitalized with an infection vs hospitalized controls (148 204 children, 244 421 young adults, 348 420 middle-aged adults, and 321 677 older adults) (Table), 10 comparisons between individuals hospitalized with an infection vs the general population (1 106 145 matched pairs), and 45 comparisons between individuals hospitalized with different infections (median [IQR] sample size, 30 766 [11 856-104 336] individuals per comparison after matching). Participant races recorded in the health records were as follows: 6091 American Indian or Alaska Native (0.6%), 27 296 Asian (2.6%), 179 739 Black or African American (16.9%), 8070 Native Hawaiian or Other Pacific Islander (0.8%), 693 824 White (65.3%), 33 241 other (3.1%), and 114 461 unknown (10.8%). Participant ethnicities recorded in the health records were as follows: 110 695 Hispanic or Latino (10.4%), 733 890 not Hispanic or Latino (69.1%), and 218 137 unknown (20.5%).

**Table.** Sample Sizes, Age, and Sex Distribution of the Different Cohorts Stratified by Age Before Matching

Cohort	Children	Adults		
		Young	Middle aged	Older
**Bone infections**				
No.	4196	12 721	33 672	22 195
Age, mean (SD)	8.9 (5.3)	34.7 (6.9)	55.4 (5.5)	72.2 (5.3)
F:M	0.67	0.50	0.44	0.60
**Cardiac infections**				
No.	1278	10 571	13 512	13 850
Age, mean (SD)	8.6 (6.7)	32.7 (6.8)	55.5 (5.6)	73.2 (5.4)
F:M	0.72	0.90	0.63	0.75
**GI infections**				
No.	19 253	23 941	38 215	38 830
Age, mean (SD)	5.9 (5.8)	32.5 (7.4)	55.7 (5.6)	73.1 (5.4)
F:M	0.89	1.24	1.09	1.24
**Hepatitides**				
No.	552	6028	6985	2938
Age, mean (SD)	10.6 (6.3)	32.9 (6.9)	54.9 (5.5)	71.4 (5.2)
F:M	1.12	0.99	0.56	0.74
**Infectious encephalitis**				
No.	249	256	437	494
Age, mean (SD)	5.9 (6.1)	32.4 (7.8)	56.3 (5.5)	73.0 (5.4)
F:M	0.89	0.96	0.86	0.91
**Meningitides**				
No.	3368	3403	2648	1446
Age, mean (SD)	5.2 (6.2)	31.4 (7.2)	54.7 (5.7)	71.9 (5.1)
F:M	0.74	1.05	0.91	0.89
**RTI**				
No.	66 478	25 369	46 572	51 278
Age, mean (SD)	2.9 (4.6)	31.9 (7.5)	54.3 (8.6)	73.4 (5.5)
F:M	0.77	1.06	0.87	0.93
**Skin infections**				
No.	39 421	94 395	139 462	105 217
Age, mean (SD)	6.8 (6.0)	33.3 (7.0)	55.1 (5.6)	72.8 (5.4)
F:M	0.86	0.85	0.69	0.90
**STI**				
No.	1854	39 487	22 942	5186
Age, mean (SD)	15.6 (3.9)	30.9 (6.8)	53.6 (5.4)	70.1 (4.8)
F:M	6.69	2.62	0.57	0.61
**UTI**				
No.	23 464	73 748	120 112	181 734
Age, mean (SD)	7.3 (6.7)	31.9 (7.4)	56.1 (5.6)	74.1 (5.5)
F:M	2.64	3.96	1.89	1.78
**Hospitalized controls without infection**				
No.	450 667	795 638	892 130	711 393
Age, mean (SD)	7.7 (6.0)	31.7 (7.2)	55.2 (5.6)	72.4 (5.4)
F:M	0.82	1.83	0.95	1.02
**General population**				
No.	4 351 083	5 169 584	4 961 135	2 759 374
Age, mean (SD)	8.6 (5.9)	31.4 (7.8)	54.3 (6.0)	70.6 (4.8)
F:M	0.89	1.30	1.29	1.25

Abbreviations: F, female; GI, gastrointestinal; M, male; RTI, respiratory tract infection; STI, sexually transmitted infection; UTI, urinary tract infection.

The most recorded infections (and noninfectious events in controls) on hospital admission are summarized in eResults 1 in Supplement 1. For instance, the most common skin infection across age groups was cellulitis, the most common RTI was influenza in adults and respiratory syncytial virus bronchiolitis in children, and the most common STI in middle-aged and older adults was HIV. In the cohort without infection, the most coded acute events were injury and poisoning for children, peripartum events for young adults, diabetes for middle-aged adults, and atherosclerosis in older adults.

### Comparison With Hospitalizations for Noninfectious Causes and the General Population

**Figure 1.**
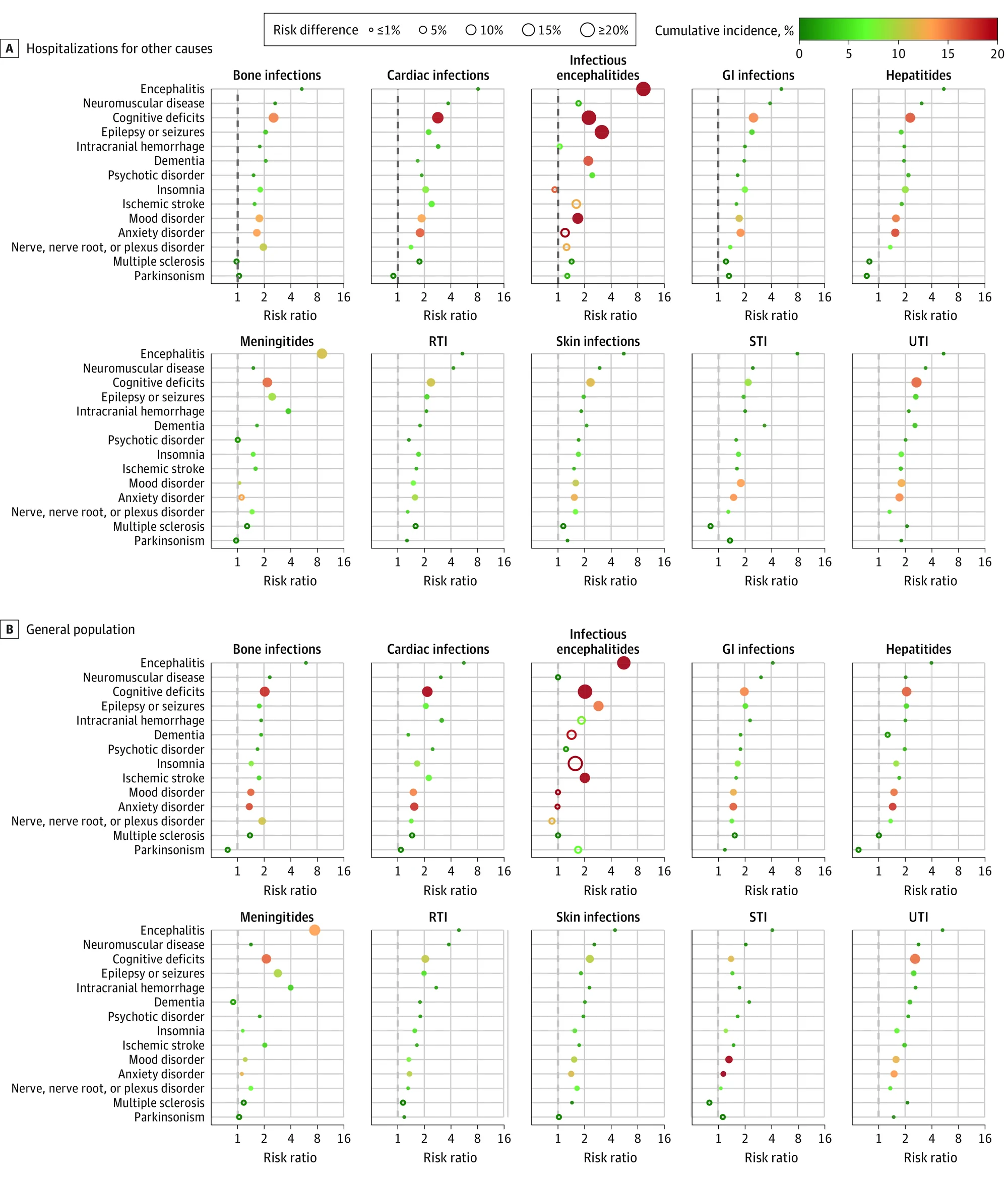
Dot Plots Showing the Comparison of the Risk of Each Outcome Between 15 Patients Hospitalized With Infections and Patients Hospitalized for Other Causes and the General Population The location of each dot on the x-axis represents the risk ratio of the disorder occurring within 2 years; its size represents the absolute risk difference, and its color represents the 2-year risk of the condition after the infection. Dots that are filled represent statistically significant risks after Bonferroni correction, whereas empty dots denote no statistical significance. Similar figure by age groups can be found in eFigures 2 to 5 in Supplement 1. GI indicates gastrointestinal; RTI, respiratory tract infection; STI, sexually transmitted infection; UTI, urinary tract infection.

Compared with matched cohorts hospitalized for noninfective causes, those hospitalized with an infection had a significantly higher associated risk of most psychiatric and neurologic conditions: relative risks were significantly greater than 1 after Bonferroni correction (dots filled and to the right of the null line in Figure 1) for 116 of 140 infection-disorder pairs (Figure 1A and eTable 1 in Supplement 1). The disorder with the highest RMTL ratio was encephalitis (eg, after cardiac infections, RMTL ratio, 6.54; 95 CI, 3.78-11.31; *P* = 2.0 × 10^−11^).

Although encephalitis presented the highest relative risk, it is a rare outcome of hospitalization with infections as seen by the small dots in Figure 1 (median absolute risk increase 0.45%). By contrast, cognitive deficits were associated with the highest absolute risk increase as depicted by the larger dots in Figure 1 with a median absolute risk increase of 8.76% and an absolute risk increase more pronounced after certain infections (eg, among cardiac infections, risk difference, 12.37%; 19.08% [95% CI, 18.51-19.65] vs 6.71% [95% CI, 6.36-7.07]).

Similar patterns were observed when patients hospitalized with an infection were compared with the general population (Figure 1B and eTable 2 in Supplement 1), with slightly higher RMTL ratios than in comparisons with hospitalized controls (mean [SD] difference in RMTL ratio, 0.19 [0.93]; paired *t* test *P* = .01). Relative risk was highest for encephalitis, whereas absolute risk was greatest for cognitive deficits.

### Comparison Between Infections

**Figure 2.**
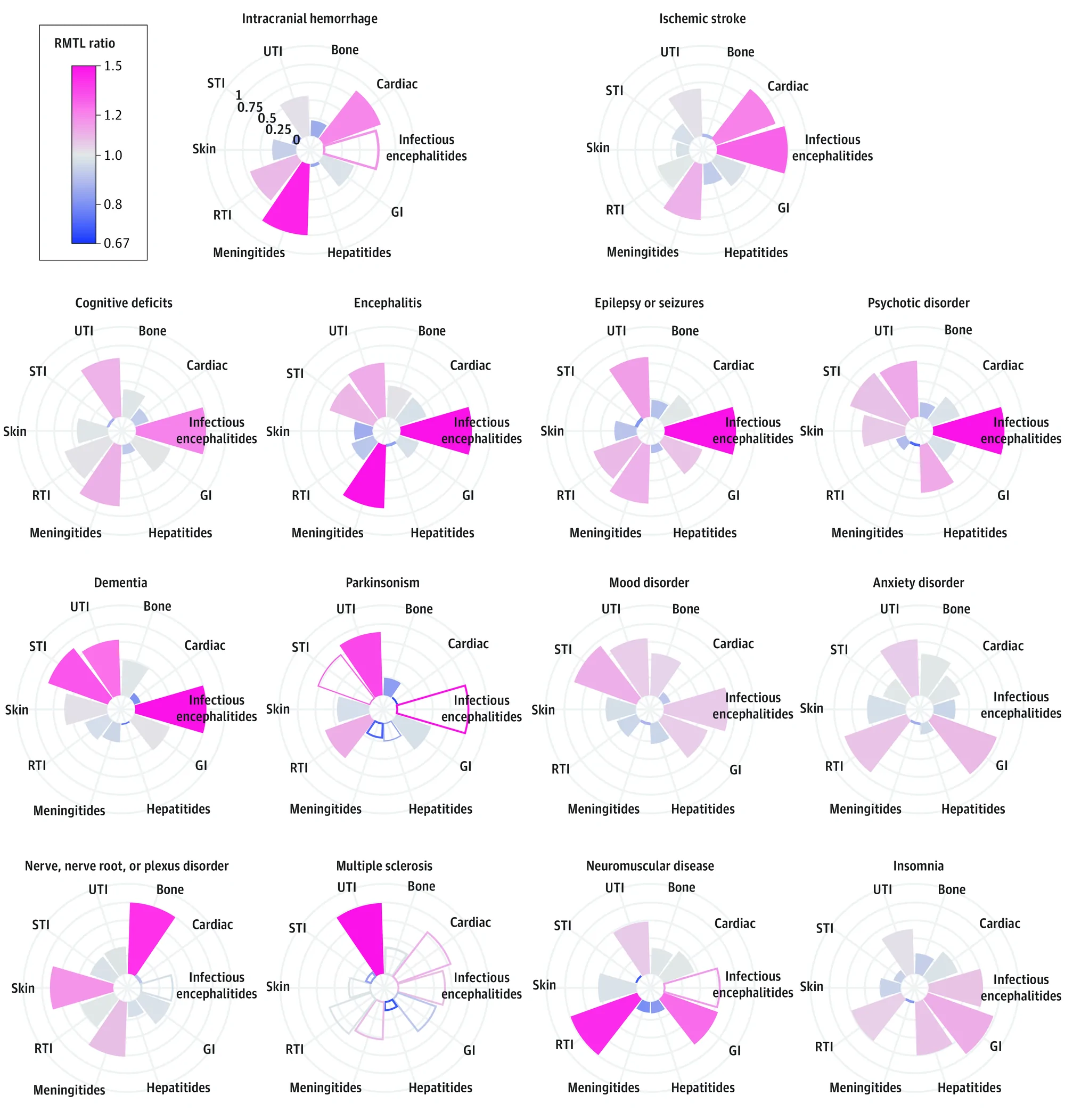
Radar Plots Showing Summary Results for the Head-to-Head Comparisons Between Patients Hospitalized With Infections Each panel represents the risk of 1 disorder after each infection. The color of the spoke encodes the median restricted mean time lost (RMTL) ratio for the comparison between the corresponding infection and all other infections. It is kept empty if the risk did not differ from that seen in the control group of those hospitalized for another reason (ie, if the RMTL ratio for the comparison with the control group was not significantly different from 1). The length of each spoke encodes the P score (graduations provided in the top left panel) representing the specificity of the infection for the disorder. Similar figure by age groups can be found in eFigure 1 in Supplement 1. GI indicates gastrointestinal; RTI, respiratory tract infection; STI, sexually transmitted infection; UTI, urinary tract infection.

In 45 head-to-head comparisons between infections (median [IQR] sample, 30 766 [11 856-104 336]) (Figure 2 and eFigure 1 in Supplement 1), infectious encephalitides were the leading risk factor for 7 of 14 disorders and ranked among the top 3 for 12 of 14 disorders, indicated by high P scores in Figure 2. Other leading infectious risk factors were disorder specific as follows: cardiac infections for intracranial hemorrhage and ischemic stroke (P score of 0.85 and 0.88, respectively); meningitides for cognitive deficits and epilepsy and seizures (P score of 0.86 and 0.82, respectively); STI for psychotic disorder, mood disorder, and dementia (P scores of 0.83, 0.93, and 0.89, respectively); and RTI for neuromuscular disease and anxiety disorder (P scores of 0.99 and 0.92, respectively). Network meta-analyses showed no evidence of inconsistency.

### Results Stratified by Age

The increased risk of psychiatric and neurologic disorders after infection was observed across age groups, though with differences in magnitude and pattern (eFigures 2-5 in Supplement 1).

In comparison with hospitalized controls, both absolute risk differences and RMTL ratios followed an age gradient, with the lowest risks in children, followed by young adults, then middle-aged and older adults. Absolute risk differences were, on average, 0.33% lower in children (95% CI, 0.27%-0.38%; *P* < .001) and 0.42% higher in older adults (95% CI, 0.33%-0.51%; *P* < .001) than the overall average. RMTL ratios showed a similar trend, being 21% lower in children (ratio, 0.79; 95% CI, 0.74-0.84; *P* < .001) and 11% higher in older adults (ratio, 1.11; 95% CI, 1.08-1.14; *P* < .001). Age significantly moderated many disorder-specific effects (eTables 3-6 in Supplement 1), with children having lower absolute risk differences and RMTL ratios for cognitive deficits, insomnia, mood, and anxiety disorders.

In comparison with the general population, the pattern of moderation of absolute risks by age was similar (eTables 7 and 8 in Supplement 1). The pattern of relative risks differed with children showing greater RMTL ratios (ratio, 1.21; 95% CI, 1.16-1.26; *P* < .001) (eTables 9 and 10 in Supplement 1).

### Clustering of Disorders

**Figure 3.**
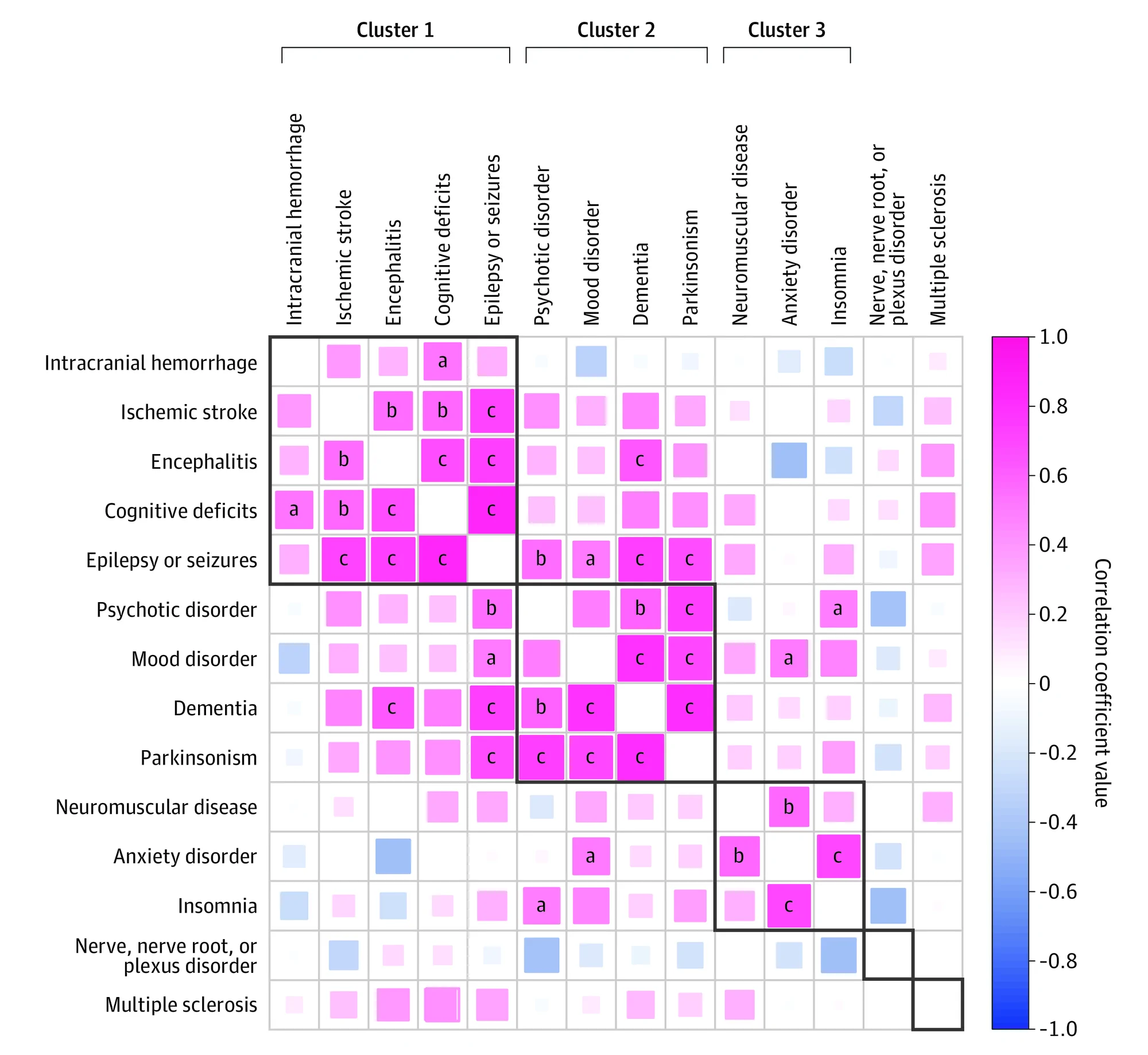
Correlation Matrix Between the Infectious Risk Factor Profiles of Different Disorders A highly positive value between 2 disorders means that the risk of both tends to increase following the same infections. Clusters are delineated by gray squares. *P* values after Bonferroni correction for 91 comparisons (all off-diagonal elements of the matrix) are represented by footnotes. ^a^*P* < .05. ^b^*P* < .01. ^c^*P* < .001.

Hierarchical clustering identified 3 clusters of disorders (Figure 3):

- Cluster 1: epilepsy and seizures, cognitive deficits, encephalitis, ischemic stroke, and intracranial hemorrhage
- Cluster 2: psychotic disorder, mood disorder, dementia, and parkinsonism.
- Cluster 3: neuromuscular disease, anxiety disorder, and insomnia.

## Discussion

This multicohort study found that risks of psychiatric and neurologic disorders are elevated 1 month to 2 years after hospitalization with infections across body systems. The increase was most associated with encephalitis (in relative risk) and cognitive deficits (in absolute risk). Although disorders show distinct infectious risk profiles, infectious encephalitides were a primary risk factor for most. Older age was particularly associated with the risk of psychiatric and neurologic disorders, whereas younger age (ie, children) was associated with lower absolute risks.

Many associations are expected and mechanistically understandable, serving as positive controls supporting the validity of our approach. Postencephalitis epilepsy is well documented,^21^ neurovascular events after cardiac infections may arise from cardioembolism and anticoagulant use, and bone or skin infections may cause nerve disorders via compression or irritation. Dementia risk after STI (notably in young adults) reflects HIV-associated neurocognitive disorder (coded under dementias in *ICD-10*), as HIV was a leading STI on admission. A prior study^22^ linked encephalitis with psychiatric and cognitive disorders but adjusted only for demographics, raising the possibility that associations resulted from shared risk factors. Our results suggest that these risks remain after extensive adjustment, persist when limited to infectious encephalitides, and extend to neurologic outcomes. Mechanisms linking infectious encephalitides to brain outcomes may involve secondary autoimmune encephalitis^21^ or direct injury to medial temporal structures and persistent neuroinflammation.^23^

Other associations were less expected. For example, cognitive deficits are associated with infectious encephalitides and meningitides (Figure 2) and their infectious risk profile strongly correlates with other cluster 1 disorders (Figure 3). This suggests a shared pathophysiology, possibly involving vascular injury and blood-brain barrier breakdown allowing peripheral immune mediator intrusion.^24^ The risk of encephalitis as an outcome was elevated after infections across body systems, possibly reflecting infection-triggered autoimmunity or initial misdiagnosis of encephalitides as systemic infections,^25^ although the latter is limited by the 30-day separation between infection and follow-up. Anxiety clusters with insomnia and neuromuscular disease (Figure 3), possibly reflecting activation of the brain-muscle signaling axis, mediated by interleukin 6 expression and muscle mitochondrial dysfunction.^26^ These explanations are plausible but speculative. Psychological and social factors (eg, HIV-related stigma leading to depression; RTI-related dyspnea causing anxiety) are also likely contributing. Mechanistic studies are therefore needed. By mapping specificity across disorders, infections, and age groups, our results provide an atlas for generating hypotheses about the infection-brain health interface, guiding future research.

The choice of control group influences relative and absolute risks. In comparisons with hospitalized controls, estimates reflect excess risk beyond that associated with hospitalization itself, whereas comparisons with the general population capture the combined outcomes of infection and hospitalization, explaining their slightly higher relative risks. Both comparisons are informative: the former helps clarify the role of infection in the etiology of neuropsychiatric conditions, whereas the latter is more relevant for public health. Differences between the 2 comparisons in age-related patterns of relative risk may partly reflect variation in baseline risk. Children in the general population are at low risk of psychiatric and neurologic conditions; therefore, small increases in risks among children hospitalized with an infection translate into high RMTL ratios.

Absolute risk differences were consistently smaller in children across comparisons with both hospitalized and general population control groups. This risk gradient might reflect variations in host immunity (influencing acute illness severity and postacute complications as seen with SARS-CoV-2 infection^27,28^), neurovascular status, and developmental or lifestyle factors that affect age groups differently. Mechanistic studies should aim to elucidate these factors, as they might inform management strategies.

Our findings have clinical implications. They suggest taking a focused history of recent or past infections when assessing patients with new-onset neurologic or psychiatric disorders, particularly when presentations are atypical (eg, late-onset psychosis or early-onset cognitive decline). Recognizing a plausible postinfectious contribution can help patients and clinicians make sense of the disorder, even when it does not alter management. If mechanistic studies delineate biologically distinct postinfectious entities, this research could underpin prognostic tools and more targeted preventive and therapeutic strategies.

These findings also inform epidemic and pandemic preparedness. The COVID-19 pandemic highlighted the need to anticipate postinfectious brain sequelae and to plan services to diagnose and manage them. When a novel pathogen emerges, authorities have little basis to predict neuropsychiatric sequelae; thus, recognition typically lags until cases accumulate. Our results offer a simple starting rule: use the primary body system affected to build an a priori map of possible postacute psychiatric and neurologic disorders and the associated age groups. Applied to SARS-CoV-2 infections (an RTI), our prepandemic data would have predicted elevated risks of encephalitis, cognitive deficits, neuromuscular disorders, and epilepsy/seizures—patterns later borne out by COVID-19 epidemiology.^11,12,16^

### Strengths and Limitations

This study has several strengths, including the large sample size, comparison of infections with each other and with separate control groups, and age-stratified analysis.

This study also has key limitations. First, EHR data are prone to misrecording or omission, which likely adds noise rather than bias if equally distributed across infections. Second, findings are limited to hospitalized patients and may not generalize to milder infections also linked to neuropsychiatric sequelae (eg, multiple sclerosis after infectious mononucleosis^8^ and psychosis after toxoplasmosis^29^). Third, despite matching on 92 factors, residual confounding remains possible, particularly from lifestyle variables poorly captured in EHR. Fourth, reverse causation may occur if infections unmask undiagnosed disorders (for example, neurogenic bladder in early multiple sclerosis predisposing to UTI). Fifth, we did not assess mediation by acute complications such as intensive care unit admission or delirium. Infection-specific risk patterns rule out the possibility that all excess risks are explained by these complications (otherwise infections increasing the risk of one disorder would also increase the risk of all others). The effects of treatments used during the acute infection were also not examined. Sixth, reasons for hospitalization are not recorded; hence, we relied on admission-day diagnostic codes, referring to index events as *hospitalizations with an infection*. Future work could also assess people hospitalized for the same noninfective indication and compare those who do and those who do not subsequently develop an infection. Seventh, risks were analyzed by affected body system rather than pathogen both to provide a framework for anticipating sequelae of emerging pathogens and because pathogens are often unrecorded. Complementary pathogen-specific studies are thus needed. Eighth, risks of some disorders were not assessed, including neurodevelopmental (given prenatal origins) and substance use disorders (due to poorly captured psychosocial factors that are particularly relevant). Ninth, recurrent or new infections (requiring hospitalization or not) during follow-up were not assessed, although previous studies^2,3^ suggest these may further increase risks of mood and psychotic disorders. Tenth, some individuals might have entered more than one cohort if they experienced multiple qualifying exposures during the study period. Privacy constraints within TriNetX preclude linking individuals across cohorts. However, the proportion meeting criteria for multiple cohorts was small and unlikely to affect the results (eResults 2 in Supplement 1).

## Conclusions

Results of this multicohort study show that hospital admission with an acute infection was associated with an increased risk of a broad range of psychiatric and neurologic disorders across ages and body systems in the following 2 years. The mechanisms underlying these associations remain unclear, but the age-related and infection-specific risk patterns suggest that multiple causal pathways might be at play. These findings will hopefully inform preparedness planning for future epidemics and pandemics and provide a basis to help design prospective and mechanistic studies seeking to explain these associations. Ultimately, uncovering the biological and clinical mechanisms linking infection to neuropsychiatric outcomes could open new avenues for prognosis, prevention, and treatment, helping to reduce the long-term burden of postinfectious neuropsychiatric disorders.

## Notes

Supplement 1. **eMethods 1.** TriNetX Network **eMethods 2.** Definition of Cohorts **eMethods 3.** Definition of Covariates **eMethods 4.** Definition of Outcomes **eMethods 5.** Details on Statistical Analyses
eReferences.
**eResults 1.** Main Infections by Body System and Main Noninfective Events on the Day of Admission **eResults 2.** Repeated Exposures **eFigure 1.** Summary of the Head-to-Head Comparisons Between Hospitalized Infections, Stratified by Age Group **eFigure 2.** Comparison of the Risk of Each Outcome Between Hospitalized Infections and Hospitalizations for Other Causes, the General Population in Children **eFigure 3.** Comparison of the Risk of Each Outcome Between Hospitalized Infections and Hospitalizations for Other Causes, the General Population in Young Adults **eFigure 4.** Comparison of the Risk of Each Outcome Between Hospitalized Infections and Hospitalisations for Other Causes, the General Population in Middle-Aged Adults **eFigure 5.** Comparison of the Risk of Each Outcome Between Hospitalized Infections and Hospitalizations for Other Causes, the General Population in Older Adults **eTable 1.** Comparison Between Each Infection and Hospitalization for Other Causes (Pooled Across Age Groups) **eTable 2.** Comparison Between Each Infection and the General Population (Pooled Across Age Groups) **eTable 3.** Moderation of RMTL Ratios by Age Group and Infection (Hospitalization for Other Causes) **eTable 4.** Moderation of RMTL Ratios by Age Group and Outcome (Hospitalization for Other Causes) **eTable 5.** Moderation of Absolute Risks by Age Group and Infection (Hospitalization for Other Causes) **eTable 6.** Moderation of Absolute Risks by Age Group and Outcome (Hospitalization for Other Causes) **eTable 7.** Moderation of RMTL Ratios by Age Group and Infection (General Population) **eTable 8.** Moderation of RMTL Ratios by Age Group and Outcome (General Population) **eTable 9.** Moderation of Absolute Risks by Age Group and Infection (General Population) **eTable 10.** Moderation of Absolute Risks by Age Group and Outcome (General Population)

Supplement 2.
Data Sharing Statement.
