# Supplementary material for: Mapping 2-Year Psychiatric and Neurologic Risks After Infections Across Body Systems and Age Groups
Source: JAMA Psychiatry. 2026 Jul 15:e261904. Online ahead of print. doi: 10.1001/jamapsychiatry.2026.1904 (PMC13373730; doi:10.1001/jamapsychiatry.2026.1904)
Supplement: Supplement 1. — eMethods 1. TriNetX Network eMethods 2. Definition of Cohorts eMethods 3. Definition of Covariates eMethods 4. Definition of Outcomes eMethods 5. Details on Statistical Analyses eReferences. eResults 1. Main Infections by Body System and Main Noninfective Events on the Day of Admission eResults 2. Repeated Exposures eFigure 1. Summary of the Head-to-Head Comparisons Between Hospitalized Infections, Stratified by Age Group eFigure 2. Comparison of the Risk of Each Outcome Between Hospitalized Infections and Hospitalizations for Other Causes, the General Population in Children eFigure 3. Comparison of the Risk of Each Outcome Between Hospitalized Infections and Hospitalizations for Other Causes, the General Population in Young Adults eFigure 4. Comparison of the Risk of Each Outcome Between Hospitalized Infections and Hospitalisations for Other Causes, the General Population in Middle-Aged Adults eFigure 5. Comparison of the Risk of Each Outcome Between Hospitalized Infections and Hospitalizations for Other Causes, the General Population in Older Adults eTable 1. Comparison Between Each Infection and Hospitalization for Other Causes (Pooled Across Age Groups) eTable 2. Comparison Between Each Infection and the General Population (Pooled Across Age Groups) eTable 3. Moderation of RMTL Ratios by Age Group and Infection (Hospitalization for Other Causes) eTable 4. Moderation of RMTL Ratios by Age Group and Outcome (Hospitalization for Other Causes) eTable 5. Moderation of Absolute Risks by Age Group and Infection (Hospitalization for Other Causes) eTable 6. Moderation of Absolute Risks by Age Group and Outcome (Hospitalization for Other Causes) eTable 7. Moderation of RMTL Ratios by Age Group and Infection (General Population) eTable 8. Moderation of RMTL Ratios by Age Group and Outcome (General Population) eTable 9. Moderation of Absolute Risks by Age Group and Infection (General Population) eTable 10. Moderation of Absolute Risks by Age Group and Outcome (General Popul [file jamapsychiatry-e261904-s001.pdf]

Supplementary Online Content

Taquet M, Oliver P, Mezher A, et al. Mapping 2-year psychiatric and neurologic risks after infections across body systems and age groups. *JAMA Psychiatry*. Published online July 15, 2026. doi:10.1001/jamapsychiatry.2026.1904

eMethods 1. TriNetX Network

eMethods 2. Definition of Cohorts

eMethods 3. Definition of Covariates

eMethods 4. Definition of Outcomes

eMethods 5. Details on Statistical Analyses

eReferences.

eResults 1. Main Infections by Body System and Main Non-Infective Events on the Day of Admission

eResults 2. Repeated Exposures

eFigure 1. Summary of the Head-to-Head Comparisons Between Hospitalised Infections, Stratified by Age Group

eFigure 2. Comparison of the Risk of Each Outcome Between Hospitalised Infections and (A) Hospitalisations for Other Causes and (B) the General Population in Children

eFigure 3. Comparison of the Risk of Each Outcome Between Hospitalised Infections and (A) Hospitalisations for Other Causes and (B) the General Population in Young Adults

eFigure 4. Comparison of the Risk of Each Outcome Between Hospitalised Infections and (A) Hospitalisations for Other Causes and (B) the General Population in Middle-Aged Adults

eFigure 5. Comparison of the Risk of Each Outcome Between Hospitalised Infections and (A) Hospitalisations for Other Causes and (B) the General Population in Older Adults

eTable 1. Comparison Between Each Infection and Hospitalisation for Other Causes (Pooled Across Age Groups)

eTable 2. Comparison Between Each Infection and the General Population (Pooled Across Age Groups)

eTable 3. Moderation of RMTL Ratios by Age Group and Infection (Hospitalisation for Other Causes)

© 2026 Taquet M et al. *JAMA Psychiatry*.

eTable 4. Moderation of RMTL Ratios by Age Group and Outcome (Hospitalisation for Other Causes)

eTable 5. Moderation of Absolute Risks by Age Group and Infection (Hospitalisation for Other Causes)

eTable 6. Moderation of Absolute Risks by Age Group and Outcome (Hospitalisation for Other Causes)

eTable 7. Moderation of RMTL Ratios by Age Group and Infection (General Population)

eTable 8. Moderation of RMTL Ratios by Age Group and Outcome (General Population)

eTable 9. Moderation of Absolute Risks by Age Group and Infection (General Population)

eTable 10. Moderation of Absolute Risks by Age Group and Outcome (General Population)

This supplementary material has been provided by the authors to give readers additional information about their work.

© 2026 Taquet M et al. *JAMA Psychiatry*.

Supplementary Methods

eMethods 1. TriNetX network

This section largely replicates our previous description of the network.[1]

Legal and ethical status
TriNetX's networks are compliant with the Health Insurance Portability and Accountability Act (HIPAA), the US federal law which protects the privacy and security of healthcare data. TriNetX is certified to the ISO 27001:2013 standard and maintains an Information Security Management System (ISMS) to ensure the protection of the healthcare data it has access to and to meet the requirements of the HIPAA Security Rule. Any data displayed on the TriNetX Platform in aggregate form, or any patient level data provided in a data set generated by the TriNetX Platform, only contains de-identified data as per the de-identification standard defined in Section §164.514(a) of the HIPAA Privacy Rule. The process by which the data is de-identified is attested to through a formal determination by a qualified expert as defined in Section §164.514(b)(1) of the HIPAA Privacy Rule. This formal determination by a qualified expert, refreshed in December 2020, supersedes the need for TriNetX's previous waiver from the Western Institutional Review Board (IRB). The network contains data that are provided by participating Health Care Organizations (HCOs), each of which represents and warrants that it has all necessary rights, consents, approvals and authority to provide the data to TriNetX under a Business Associate Agreement (BAA), so long as their name remains anonymous as a data source and their data are utilized for research purposes. The data shared through the TriNetX Platform are attenuated to ensure that they do not include sufficient information to facilitate the determination of which HCO contributed which specific information about a patient. The HCOs warrant that they have all necessary rights, consents, approvals, and authority to provide the data to TriNetX, as long as their name remains anonymous as a data source and their data are used for research purposes. Keeping the identity of participating HCOs from the researchers using the data also contribute to complying with legal frameworks and ethical guidelines guarding against data re-identification.

Acquisition of data, quality control, and other procedures
The data are stored onboard a TriNetX appliance – a physical server residing at the institution's data centre or a virtual hosted appliance. The TriNetX platform is a fleet of these appliances connected into a federated network able to broadcast queries to each appliance. Results are subsequently collected and aggregated.

Once the data are sent to the network, they are mapped to a standard and controlled set of clinical terminologies and undergo a data quality assessment including 'data cleaning' that rejects records which do not meet the TriNetX quality standards. HIPAA compliance of the clinical patient data is achieved using de-identification. Different data modalities are available in the network. They include demographics (coded to HL7 version 3 administrative standards), diagnoses (represented by ICD-10-CM codes), procedures (coded in ICD-10-PCS or CPT), and measurements (coded to LOINC). While extensive information is provided about patients' diagnoses and procedures, other variables (such as socioeconomic and lifetime factors are not comprehensively represented).

The data from a typical HCO generally go back around 7 years, with some going back 16 years. The data are continuously updated. HCOs update their data at various times, with most refreshing every 1, 2, or 4 weeks.

Data quality assessment followed a standardised strategy wherein the data are reviewed for conformance (adherence to specified standards and formats), completeness (quantifying data presence or absence) and plausibility (believability of the data from a clinical perspective). There are pre-defined metrics for each of the above assessment categories. Results for these metrics are visualised and reviewed for each new site that joins the network as well as on an ongoing basis. Any identified issue is communicated to the data provider and resolved before continuing data collection.

The basic formatting of contributed data is also checked (e.g. to ensure that dates are properly represented). Records are checked against a list of required fields (e.g., patient identifier) and records for which the required information is missing are rejected. Referential integrity checking is done to ensure that data spanning multiple database tables can be successfully joined together. As the data are refreshed, changes in volume of data over time is monitored to ensure data validity. At least one non-demographic fact for each patient is required for them to be counted in the dataset. Patient records with only demographics information are discarded.

© 2026 Taquet M et al. *JAMA Psychiatry.*

The software also undergoes quality control. The engineers testing the software are independent from the engineers developing it. Each test code is checked by two independent testing engineers. Each piece of software is tested extensively against a range of synthetic data (i.e. generated for the purpose of testing) for which the expected output is established independently. If the software fails to return this output, then the software is deemed to have failed the test and is examined and modified accordingly. For statistical software (including that used for propensity score matching, for Kaplan-Meier analysis, etc), an additional quality control step is implemented. Two independent codes are written in two different programming languages (typically R and python) and the statistical results are compared. If discrepancies are identified, then the codes are deemed to have failed the test and are examined and modified accordingly. All the code is reviewed independently by another engineer.

The test strategy follows three levels of granularity:

1. Unit tests: These test specific blocks, or units, of code that perform specific actions (e.g. querying the database).
2. Integration tests: These ensure that different components are working together correctly.
3. End-to-end tests: These tests run the entire system and check the final output.

© 2026 Taquet M et al. *JAMA Psychiatry*.

eMethods 2. Definition of cohorts

We defined three sets of cohorts:

- **Primary cohorts**: having an infection recorded on the same day as being hospitalised

- **Hospitalised controls**: being hospitalised without an infection

- **General population**: having at least one outpatient visit (including primary care visit) during the study period (2013–2018).

Hospitalisation was defined as any instance of the following code within TriNetX:

- Visit: Inpatient Encounter
- Visit: Inpatient Non-acute
- Critical Care Services
- Visit: Short Stay
- Hospital Inpatient Services
- Inpatient Consultations
- Initial Hospital Care

For the **primary cohorts**, we identified infections in ten different body systems: cardiac infections, respiratory tract infections, meningitis, encephalitis, gastrointestinal infections, urinary tract infections, skin infections, bone infections, sexually transmitted infections, and hepatitis. Where relevant, we only included acute forms of an infection, such as including acute but not chronic forms of viral hepatitis. We included cases where acute and chronic infection could not be discriminated, such as HIV. In addition, each cohort required that a patient be hospitalised on the same date that their infectious diagnostic code was recorded to ensure that only new diagnoses and acute infections were considered. The ICD-10-CM codes used for defining infections in each body system are detailed below:

Cardiac Infection:

- B33.21 Viral endocarditis
- B33.22 Viral myocarditis
- B33.23 Viral pericarditis
- I30.1 Infective pericarditis
- I33.0 Acute and subacute infective endocarditis
- I38 Endocarditis, valve unspecified
- I40.0 Infective myocarditis

Respiratory Tract Infection:

- J00 Acute nasopharyngitis [common cold]
- J02.0 Streptococcal pharyngitis
- J02.8 Acute pharyngitis due to other specified organisms
- J03.0 Streptococcal tonsillitis
- J03.8 Acute tonsillitis due to other specified organisms
- J09.X Influenza due to identified novel influenza A virus
- J10.0 Influenza due to other identified influenza virus with pneumonia
- J10.1 Influenza due to other identified influenza virus with other respiratory manifestations
- J10.2 Influenza due to other identified influenza virus with gastrointestinal manifestations
- J10.8 Influenza due to other identified influenza virus with other manifestations
- J12.0 Adenoviral pneumonia
- J12.2 Parainfluenza virus pneumonia
- J12.3 Human metapneumovirus pneumonia
- J12.81 Pneumoniae due to SARS-associated coronavirus
- J13 Pneumoniae due to Streptococcus pneumoniae
- J14 Pneumoniae due to Haemophilus influenzae
- J15.0 Pneumonia due to Klebsiella pneumoniae
- J15.1 Pneumoniae due to Pseudomonas
- J15.211 Pneumoniae due to Methicillin susceptible Staphylococcus aureus
- J15.212 Pneumoniae due to Methicillin resistant Staphylococcus aureus

© 2026 Taquet M et al. *JAMA Psychiatry*.

- J15.29 Pneumoniae due to other staphylococcus
- J15.3 Pneumoniae due to Streptococcus, group B
- J15.4 Pneumoniae due to other streptococci
- J15.5 Pneumoniae due to Escherichia coli
- J15.6 Pneumoniae due to other Gram-negative bacteria
- J15.7 Pneumoniae due to Mycoplasma pneumoniae
- J15.8 Pneumoniae due to other specified bacteria
- J16.0 Chlamydial pneumoniae
- J16.8 Pneumoniae due to other specified infectious organisms
- J20.0 Acute bronchitis due to Mycoplasma pneumoniae
- J20.1 Acute bronchitis due to Haemophilus influenzae
- J20.2 Acute bronchitis due to streptococcus
- J20.3 Acute bronchitis due to coxsackievirus
- J20.4 Acute bronchitis due to parainfluenza virus
- J20.5 Acute bronchitis due to respiratory syncytial virus
- J20.6 Acute bronchitis due to rhinovirus
- J20.7 Acute bronchitis due to echovirus
- J20.8 Acute bronchitis due to other specified organisms
- J21.0 Acute bronchiolitis due to respiratory syncytial virus
- J21.1 Acute bronchiolitis due to human metapneumovirus
- J21.8 Acute bronchiolitis due to other specified organisms

Meningitis:
- A87 Viral meningitis
- G00 Bacterial meningitis, not elsewhere classified
- G01 Meningitis in bacterial diseases classified elsewhere
- G02 Meningitis in other infectious and parasitic diseases classified elsewhere

Encephalitis:
- A85.0 Enteroviral encephalitis
- A85.8 Other specified viral encephalitis
- B00.4 Herpes viral encephalitis

Gastrointestinal Tract Infection:
- A00 Cholera
- A01 Typhoid and paratyphoid fevers
- A02 Other salmonella infections
- A03 Shigellosis
- A04 Other bacterial intestinal infections
- A06 Amebiasis
- A07 Other protozoal intestinal diseases
- A08 Viral and other specified intestinal infections
- A09 Infectious gastroenteritis and colitis, unspecified

Urinary Tract Infection:
- N10 Acute pyelonephritis
- N39.0 Urinary tract infection, site not specified

Skin Infection:
- A69.2 Lyme disease
- B00.0 Eczema herpeticum
- B00.1 Herpes vesicular dermatitis
- B01 Varicella [chickenpox]
- B02 Zoster [herpes zoster]
- B05 Measles
- B07 Viral warts
- B08.1 Molluscum contagiosum
- B35 Dermatophytosis
- B37.2 Candidiasis of skin and nail
- B86 Scabies
- L00 Staphylococcal scalded skin syndrome
- L01 Impetigo

© 2026 Taquet M et al. *JAMA Psychiatry*.

- L02 Cutaneous abscess, furuncle and carbuncle
- L03 Cellulitis and acute lymphangitis
- L08 Other local infections of skin and subcutaneous tissue
- M72.6 Necrotising fasciitis

Bone Infections:

- M86 Osteomyelitis

Sexually Transmitted Infection:

- A51 Early syphilis
- A53 Other and unspecified syphilis
- A54 Gonococcal infection
- A55 Chlamydial lymphogranuloma (venereum)
- A56 Other sexually transmitted chlamydial diseases
- A57 Chancroid
- A59 Trichomoniasis
- A60 Anogenital herpesviral [herpes simplex] infections
- A63 Other predominantly sexually transmitted diseases, not elsewhere classified
- B20 Human immunodeficiency virus [HIV] disease

Hepatitis:

- B15 Acute Hepatitis A
- B16 Acute Hepatitis B
- B17 Other acute viral hepatitis

For the **control cohorts**, to ensure we did not include any patients hospitalised due to infection (for the **hospitalised controls**) or seen as an outpatient for an infection at the index date (for the **general population**), we excluded those with a diagnostic code for an infection (or diagnostic codes that can be used in infectious contexts such as aspiration pneumonia), prescription of antimicrobial, or serological/microbiological evidence of infection on the day of admission to hospital or up to one month before. That is because some people might be diagnosed with an infection in the community and then be hospitalised for that same infection a few days or weeks later. People with an infection recorded at an earlier date (> 1 month before hospitalisation/outpatient visit) could still be included in the control group.

Infection and other diagnostic codes

- N39.0 Urinary tract infection, site not specified
- N10 Acute pyelonephritis
- B00.4 Herpesviral encephalitis
- A85.8 Other specified viral encephalitis
- A85.0 Enteroviral encephalitis
- A04 Other bacterial intestinal infections
- A08 Viral and other specified intestinal infections
- A04.8 Other specified bacterial intestinal infections
- A00 Cholera
- A01 Typhoid and paratyphoid fevers
- A02 Other salmonella infections
- A03 Shigellosis
- A06 Amebiasis
- A07 Other protozoal intestinal diseases
- A09 Infectious gastroenteritis and colitis, unspecified
- H10 Conjunctivitis
- M86 Osteomyelitis
- L08 Other local infections of skin and subcutaneous tissue
- B35.3 Tinea pedis
- B35.4 Tinea corporis
- B35.0 Tinea barbae and tinea capitis
- L03 Cellulitis and acute lymphangitis
- L02 Cutaneous abscess, furuncle and carbuncle
- B05 Measles

© 2026 Taquet M et al. *JAMA Psychiatry.*

- B86 Scabies
- B01 Varicella [chickenpox]
- B02 Zoster [herpes zoster]
- B07 Viral warts
- B00.0 Eczema herpeticum
- B00.1 Herpesviral vesicular dermatitis
- A69.2 Lyme disease
- B35 Dermatophytosis
- L01 Impetigo
- B08.1 Molluscum contagiosum
- B37.2 Candidiasis of skin and nail
- L00 Staphylococcal scalded skin syndrome
- M72.6 Necrotizing fasciitis
- G00 Bacterial meningitis, not elsewhere classified
- A87 Viral meningitis
- G00.2 Streptococcal meningitis
- G00.0 Hemophilus meningitis
- G00.1 Pneumococcal meningitis
- G00.8 Other bacterial meningitis
- G01 Meningitis in bacterial diseases classified elsewhere
- G02 Meningitis in other infectious and parasitic diseases classified elsewhere
- A59.0 Urogenital trichomoniasis
- A51 Early syphilis
- A53 Other and unspecified syphilis
- A54 Gonococcal infection
- A55 Chlamydial lymphogranuloma (venereum)
- A56 Other sexually transmitted chlamydial diseases
- A57 Chancroid
- A59 Trichomoniasis
- A60 Anogenital herpesviral [herpes simplex] infections
- A63 Other predominantly sexually transmitted diseases, not elsewhere classified
- B20 Human immunodeficiency virus [HIV] disease
- B16 Acute hepatitis B
- B17 Other acute viral hepatitis
- B15 Acute hepatitis A
- J20.0 Acute bronchitis due to Mycoplasma pneumoniae
- J20.1 Acute bronchitis due to Hemophilus influenzae
- J20.2 Acute bronchitis due to streptococcus
- J20.3 Acute bronchitis due to coxsackievirus
- J20.4 Acute bronchitis due to parainfluenza virus
- J20.5 Acute bronchitis due to respiratory syncytial virus
- J20.6 Acute bronchitis due to rhinovirus
- J20.7 Acute bronchitis due to echovirus
- J20.8 Acute bronchitis due to other specified organisms
- J21.0 Acute bronchiolitis due to respiratory syncytial virus
- J21.1 Acute bronchiolitis due to human metapneumovirus
- J21.8 Acute bronchiolitis due to other specified organisms
- J15.0 Pneumonia due to Klebsiella pneumoniae
- J15.1 Pneumonia due to Pseudomonas
- J13 Pneumonia due to Streptococcus pneumoniae
- J14 Pneumonia due to Hemophilus influenzae
- J16.0 Chlamydial pneumonia
- J15.3 Pneumonia due to streptococcus, group B
- J15.4 Pneumonia due to other streptococci
- J15.5 Pneumonia due to Escherichia coli
- J15.6 Pneumonia due to other Gram-negative bacteria
- J15.7 Pneumonia due to Mycoplasma pneumoniae

© 2026 Taquet M et al. *JAMA Psychiatry*.

- J15.8 Pneumonia due to other specified bacteria
- J16.8 Pneumonia due to other specified infectious organisms
- J15.29 Pneumonia due to other staphylococcus
- J15.211 Pneumonia due to Methicillin susceptible Staphylococcus aureus
- J15.212 Pneumonia due to Methicillin resistant Staphylococcus aureus
- J00 Acute nasopharyngitis [common cold]
- J02.0 Streptococcal pharyngitis
- J02.8 Acute pharyngitis due to other specified organisms
- J03.0 Streptococcal tonsillitis
- J03.8 Acute tonsillitis due to other specified organisms
- J12.0 Adenoviral pneumonia
- J10.0 Influenza due to other identified influenza virus with pneumonia
- J10.1 Influenza due to other identified influenza virus with other respiratory manifestations
- J10.8 Influenza due to other identified influenza virus with other manifestations
- J12.2 Parainfluenza virus pneumonia
- J12.3 Human metapneumovirus pneumonia
- J12.81 Pneumonia due to SARS-associated coronavirus
- J09.X Influenza due to identified novel influenza A virus
- J98.8 Other specified respiratory disorders
- J98.4 Other disorders of lung
- J60-J70 Lung diseases due to external agents
- L00-L08 Infections of the skin and subcutaneous tissue
- A50-A64 Infections with a predominantly sexual mode of transmission
- T81.4 Infection following a procedure
- O23 Infections of genitourinary tract in pregnancy
- O41.1 Infection of amniotic sac and membranes
- T83.5 Infection and inflammatory reaction due to prosthetic device, implant and graft in urinary system
- T81.49 Infection following a procedure, other surgical site
- J06 Acute upper respiratory infections of multiple and unspecified sites
- H66.9 Otitis media, unspecified
- H60 Otitis externa
- B37.0 Candidal stomatitis
- I38 Endocarditis, valve unspecified
- B97.89 Other viral agents as the cause of diseases classified elsewhere
- A00-B99 Certain infectious and parasitic diseases
- M00-M02 Infectious arthropathies
- J09-J18 Influenza and pneumonia
- J20-J22 Other acute lower respiratory infections
- J00-J06 Acute upper respiratory infections
- L00-L08 Infections of the skin and subcutaneous tissue
- M00-M02 Infectious arthropathies
- M25.60 Stiffness of unspecified joint, not elsewhere classified
- K81.0 Acute cholecystitis
- G03 Meningitis due to other and unspecified causes
- G04 Encephalitis, myelitis and encephalomyelitis
- I33 Acute and subacute endocarditis
- K35 Acute appendicitis
- L03 Cellulitis and acute lymphangitis
- B02 Zoster [herpes zoster]
- N39.0 Urinary tract infection, site not specified
- R50 Fever of other and unknown origin

Prescription of antimicrobials

- AM000 ANTIMICROBIALS
- 31099-5 Amikacin [Mass/volume] in Serum or Plasma

© 2026 Taquet M et al. *JAMA Psychiatry*.

Serological (and other microbiological) evidence of infection

- 1012134 Microbiology Procedures
- 1011223 Urinalysis Procedures
- 10676-5 Hepatitis C virus RNA [Units/volume] (viral load) in Serum or Plasma by Probe with amplification
- 10900-9 Hepatitis B virus surface Ab [Presence] in Serum by Immunoassay
- 11006-4 Borrelia burgdorferi Ab [Presence] in Serum
- 11011-4 Hepatitis C virus RNA [Units/volume] (viral load) in Serum or Plasma by NAA with probe detection
- 11259-9 Hepatitis C virus RNA [Presence] in Serum or Plasma by NAA with probe detection
- 11268-0 Streptococcus pyogenes [Presence] in Throat by Organism specific culture
- 11475-1 Microorganism identified in Specimen by Culture
- 11483-5 Varicella zoster virus DNA [Presence] in Specimen by NAA with probe detection
- 11545-1 Microscopic observation [Identifier] in Specimen by Acid fast stain
- 11546-9 Microscopic observation [Identifier] in Specimen by Other stain
- 13315-7 Bacteria identified in 24 hour Urine by Culture
- 13317-3 Methicillin resistant Staphylococcus aureus [Presence] in Specimen by Organism specific culture
- 13503-8 Borrelia burgdorferi IgM band pattern [Interpretation] in Serum by Immunoblot
- 13949-3 Cytomegalovirus IgG Ab [Presence] in Serum or Plasma by Immunoassay
- 13950-1 Hepatitis A virus IgM Ab [Presence] in Serum or Plasma by Immunoassay
- 13951-9 Hepatitis A virus Ab [Presence] in Serum by Immunoassay
- 13952-7 Hepatitis B virus core Ab [Presence] in Serum or Plasma by Immunoassay
- 13953-5 Hepatitis B virus e Ab [Presence] in Serum or Plasma by Immunoassay
- 13954-3 Hepatitis B virus e Ag [Presence] in Serum or Plasma by Immunoassay
- 13955-0 Hepatitis C virus Ab [Presence] in Serum or Plasma by Immunoassay
- 14367-7 Trichomonas vaginalis [Presence] in Vaginal fluid by Wet preparation
- 15209-0 Saccharomonospora viridis Ab [Presence] in Serum by Immune diffusion (ID)
- 15410-4 Varicella zoster virus IgG Ab [Presence] in Serum by Immunoassay
- 16128-1 Hepatitis C virus Ab [Presence] in Serum
- 16129-9 Hepatitis C virus IgG Ab [Presence] in Serum
- 16130-7 Herpes simplex virus 1 DNA [Presence] in Specimen by NAA with probe detection
- 16131-5 Herpes simplex virus 2 DNA [Presence] in Specimen by NAA with probe detection
- 16280-0 Human papilloma virus DNA [Presence] in Specimen by Probe with amplification
- 16599-3 Chlamydia trachomatis DNA [Presence] in Blood by NAA with probe detection
- 16933-4 Hepatitis B virus core Ab [Presence] in Serum
- 17780-8 Helicobacter pylori Ag [Presence] in Stool by Immunoassay
- 17859-0 Helicobacter pylori IgG Ab [Presence] in Serum or Plasma by Immunoassay
- 17928-3 Bacteria identified in Blood by Aerobe culture
- 17934-1 Bacteria identified in Blood by Anaerobe culture
- 18396-2 HIV 1 p24 Ag [Presence] in Serum or Plasma by Immunoassay
- 19162-7 Varicella zoster virus IgG Ab [Presence] in Serum
- 20416-4 Hepatitis C virus RNA [#/volume] (viral load) in Serum or Plasma by NAA with probe detection
- 20430-5 Culture medium [Type] in Isolate
- 20447-9 HIV 1 RNA [#/volume] (viral load) in Serum or Plasma by NAA with probe detection
- 20449-5 Borrelia burgdorferi Ab [Presence] in Serum by Immunoassay
- 20458-6 Rubella virus IgG Ab [Interpretation] in Serum
- 20479-2 Measles virus IgG Ab [Presence] in Serum
- 20507-0 Reagin Ab [Presence] in Serum by RPR
- 20966-8 Staphylococcus sp identified in Specimen by Organism specific culture
- 21003-9 Fungus identified in Specimen by Fungus stain
- 21089-8 Babesia microti DNA [Presence] in Blood by NAA with probe detection
- 21190-4 Chlamydia trachomatis DNA [Presence] in Cervix by NAA with probe detection
- 21233-2 Cryptosporidium sp Ag [Presence] in Stool
- 21262-1 Escherichia coli shiga-like toxin [Presence] in Stool by Immunoassay

© 2026 Taquet M et al. *JAMA Psychiatry*.

- 21414-8 Neisseria gonorrhoeae DNA [Presence] in Cervical mucus by NAA with probe detection
- 21416-3 Neisseria gonorrhoeae DNA [Presence] in Urine by NAA with probe detection
- 21613-5 Chlamydia trachomatis DNA [Presence] in Specimen by NAA with probe detection
- 22244-8 Cytomegalovirus IgG Ab [Presence] in Serum or Plasma
- 22314-9 Hepatitis A virus IgM Ab [Presence] in Serum
- 22322-2 Hepatitis B virus surface Ab [Presence] in Serum
- 22415-4 Mumps virus IgG Ab [Presence] in Serum
- 22496-4 Rubella virus Ab [Presence] in Serum
- 22585-4 Treponema pallidum Ab [Units/volume] in Blood
- 22587-0 Treponema pallidum Ab [Presence] in Serum
- 23826-1 Bordetella pertussis DNA [Presence] in Specimen by NAA with probe detection
- 24027-5 Streptococcus pneumoniae Ag [Presence] in Urine
- 24110-9 Treponema pallidum Ab [Presence] in Serum by Immunoassay
- 24111-7 Neisseria gonorrhoeae DNA [Presence] in Specimen by NAA with probe detection
- 24113-3 Hepatitis B virus core IgM Ab [Presence] in Serum or Plasma by Immunoassay
- 24119-0 Cytomegalovirus IgM Ab [Presence] in Serum or Plasma by Immunoassay
- 24312-1 Treponema pallidum Ab [Presence] in Serum by Agglutination
- 25296-5 Streptococcus pneumoniae Danish serotype 7F IgG Ab [Mass/volume] in Serum
- 25418-5 Mumps virus IgG Ab [Units/volume] in Serum by Immunoassay
- 25514-1 Rubella virus IgG Ab [Presence] in Serum
- 27395-3 Streptococcus pneumoniae Danish serotype 18C IgG Ab [Mass/volume] in Serum
- 27994-3 Ehrlichia chaffeensis DNA [Presence] in Blood by NAA with probe detection
- 29257-3 Mycoplasma pneumoniae DNA [Presence] in Specimen by NAA with probe detection
- 29541-0 HIV 1 RNA [Log #/volume] (viral load) in Serum or Plasma by NAA with probe detection
- 29723-4 Bordetella parapertussis DNA [Presence] in Specimen by NAA with probe detection
- 29893-5 HIV 1 Ab [Presence] in Serum or Plasma by Immunoassay
- 29908-1 Parainfluenza virus 1 RNA [Presence] in Specimen by NAA with probe detection
- 29909-9 Parainfluenza virus 2 RNA [Presence] in Specimen by NAA with probe detection
- 29910-7 Parainfluenza virus 3 RNA [Presence] in Specimen by NAA with probe detection
- 30039-2 Anaplasma phagocytophilum DNA [Presence] in Blood by NAA with probe detection
- 30075-6 Respiratory syncytial virus A RNA [Presence] in Specimen by NAA with probe detection
- 30076-4 Respiratory syncytial virus B RNA [Presence] in Specimen by NAA with probe detection
- 30083-0 Epstein Barr virus nuclear IgG Ab [Units/volume] in Serum by Immunoassay
- 30122-6 Aeromonas sp [Presence] in Specimen by Organism specific culture
- 30247-1 Cytomegalovirus DNA [#/volume] (viral load) in Serum or Plasma by NAA with probe detection
- 30339-6 Epstein Barr virus capsid IgG Ab [Presence] in Serum
- 30340-4 Epstein Barr virus capsid IgM Ab [Presence] in Serum
- 30361-0 HIV 2 Ab [Presence] in Serum or Plasma by Immunoassay
- 31147-2 Reagin Ab [Titer] in Serum by RPR
- 31201-7 HIV 1+2 Ab [Presence] in Serum or Plasma by Immunoassay
- 31204-1 Hepatitis B virus core IgM Ab [Presence] in Serum
- 31418-7 Heterophile Ab [Presence] in Serum
- 31777-6 Chlamydia trachomatis Ag [Presence] in Specimen
- 31830-3 Giardia lamblia Ag [Presence] in Stool
- 31833-7 Haemophilus influenzae A Ag [Presence] in Specimen
- 31834-5 Haemophilus influenzae B Ag [Presence] in Specimen
- 31856-8 Histoplasma capsulatum Ag [Units/volume] in Urine
- 31859-2 Influenza virus A Ag [Presence] in Specimen
- 31864-2 Influenza virus B Ag [Presence] in Specimen
- 31950-9 Respiratory syncytial virus Ag [Presence] in Specimen
- 31970-7 Streptococcus pyogenes Ag [Presence] in Serum
- 31971-5 Streptococcus pyogenes Ag [Presence] in Specimen
- 32018-4 Hepatitis A virus IgG Ab [Presence] in Serum
- 32286-7 Hepatitis C virus genotype [Identifier] in Serum or Plasma by NAA with probe detection
- 32355-0 Bacteria identified in Specimen by Respiratory culture
- 32685-0 Hepatitis B virus core IgG Ab [Presence] in Serum

© 2026 Taquet M et al. *JAMA Psychiatry*.

- 32705-6 Neisseria gonorrhoeae DNA [Presence] in Vaginal fluid by NAA with probe detection
- 32763-5 Bacteria [Presence] in Specimen by Wet preparation
- 32764-3 Clue cells [Presence] in Specimen by Wet preparation
- 32765-0 Yeast [Presence] in Specimen by Wet preparation
- 32766-8 Trichomonas vaginalis [Presence] in Specimen by Wet preparation
- 32781-7 Legionella sp Ag [Presence] in Urine
- 33764-2 Shiga toxin stx gene [Identifier] in Specimen by NAA with probe detection
- 33931-7 Lymphocyte proliferation stimulated by Borrelia burgdorferi [Ratio] in Blood
- 34147-9 Treponema pallidum IgG+IgM Ab [Presence] in Serum
- 34162-8 Hepatitis C virus IgG band pattern [Interpretation] in Serum by Immunoblot
- 34468-9 Clostridioides difficile toxin A+B [Presence] in Stool by Immunoassay
- 34487-9 Influenza virus A RNA [Presence] in Specimen by NAA with probe detection
- 34645-2 Chlamydophila pneumoniae DNA [Presence] in Specimen by NAA with probe detection
- 35275-7 Measles virus IgG Ab [Presence] in Serum by Immunoassay
- 35729-3 Chlamydia sp DNA [Presence] in Specimen by NAA with probe detection
- 35730-1 Chlamydia sp rRNA [Presence] in Specimen by Probe
- 38180-6 Hepatitis C virus RNA [log units/volume] (viral load) in Serum or Plasma by NAA with probe detection
- 38381-0 Influenza virus A cDNA [Presence] in Specimen by NAA with probe detection
- 38917-1 Human metapneumovirus RNA [Presence] in Specimen by NAA with probe detection
- 38990-8 Escherichia coli O157:H7 DNA [Presence] in Specimen by NAA with probe detection
- 39017-9 Mycobacterium tuberculosis tuberculin stimulated gamma interferon/Mitogen stimulated gamma interferon in Blood
- 39528-5 Adenovirus DNA [Presence] in Specimen by NAA with probe detection
- 40667-8 Rubella virus IgG Ab [Presence] in Serum or Plasma by Immunoassay
- 40976-3 Adenovirus DNA [Identifier] in Specimen by NAA with probe detection
- 40978-9 Human metapneumovirus RNA [Identifier] in Specimen by NAA with probe detection
- 40982-1 Influenza virus B RNA [Presence] in Specimen by NAA with probe detection
- 40987-0 Respiratory syncytial virus RNA [Identifier] in Specimen by NAA with probe detection
- 40988-8 Respiratory syncytial virus RNA [Presence] in Specimen by NAA with probe detection
- 40991-2 Rhinovirus+Enterovirus RNA [Presence] in Specimen by NAA with probe detection
- 41003-5 Human coronavirus 229E RNA [Presence] in Specimen by NAA with probe detection
- 41005-0 Human coronavirus NL63 RNA [Presence] in Specimen by NAA with probe detection
- 41009-2 Human coronavirus OC43 RNA [Presence] in Specimen by NAA with probe detection
- 41010-0 Parainfluenza virus 4 RNA [Presence] in Specimen by NAA with probe detection
- 41399-7 Herpes simplex virus 1+2 IgM Ab [Units/volume] in Serum by Immunoassay
- 41436-7 Cyclospora cayetanensis DNA [Presence] in Specimen by NAA with probe detection
- 41511-7 Trichomonas vaginalis Ag [Presence] in Vaginal fluid
- 42588-4 Bordetella parapertussis DNA [Presence] in Nasopharynx by NAA with probe detection
- 42595-9 Hepatitis B virus DNA [Units/volume] (viral load) in Serum or Plasma by NAA with probe detection
- 42768-2 HIV 1 and 2 Ab [Interpretation] in Serum Narrative
- 43304-5 Chlamydia trachomatis rRNA [Presence] in Specimen by NAA with probe detection
- 43305-2 Neisseria gonorrhoeae rRNA [Presence] in Specimen by NAA with probe detection
- 43403-5 Neisseria gonorrhoeae DNA [Presence] in Specimen by Probe with signal amplification
- 43404-3 Chlamydia trachomatis DNA [Presence] in Specimen by Probe with signal amplification
- 43408-4 Bacteria identified in Tissue by Culture
- 43874-7 Influenza virus A Ag [Presence] in Nasopharynx
- 43895-2 Influenza virus B Ag [Presence] in Nasopharynx
- 43913-3 Bordetella pertussis DNA [Presence] in Nasopharynx by NAA with probe detection
- 44563-5 Influenza virus A Ag [Presence] in Nose
- 45084-1 Chlamydia trachomatis DNA [Presence] in Vaginal fluid by NAA with probe detection
- 45323-3 Mycobacterium tuberculosis tuberculin stimulated gamma interferon [Presence] in Blood
- 46154-1 Trichomonas vaginalis rRNA [Presence] in Specimen by NAA with probe detection
- 46216-8 Mycobacterium tuberculosis tuberculin stimulated gamma interferon/Mitogen stimulated gamma interferon [Units/volume] in Control Blood

© 2026 Taquet M et al. *JAMA Psychiatry*.

- 46217-6 Mycobacterium tuberculosis stimulated gamma interferon release by CD4+ T-cells [Units/volume] in Blood
- 46455-2 Shigella sp DNA [Presence] in Specimen by NAA with probe detection
- 47000-5 Candida sp rRNA [Presence] in Vaginal fluid by Probe
- 47212-6 Chlamydia trachomatis DNA [Identifier] in Specimen by NAA with probe detection
- 47236-5 Treponema pallidum IgG+IgM Ab [Presence] in Serum by Immunoassay
- 47238-1 Treponema pallidum IgG Ab [Presence] in Serum by Immunoassay
- 47387-6 Neisseria gonorrhoeae DNA [Presence] in Genital specimen by NAA with probe detection
- 48159-8 Hepatitis C virus Ab Signal/Cutoff in Serum or Plasma by Immunoassay
- 48345-3 HIV 1+O+2 Ab [Presence] in Serum or Plasma
- 48646-4 Yersinia sp DNA [Identifier] in Specimen by NAA with probe detection
- 48683-7 Streptococcus agalactiae DNA [Presence] in Specimen by NAA with probe detection
- 49178-7 Epstein Barr virus Ab [Presence] in Serum
- 49521-8 Influenza virus A H1 RNA [Presence] in Specimen by NAA with probe detection
- 49524-2 Influenza virus A H3 RNA [Presence] in Specimen by NAA with probe detection
- 49580-4 HIV 1+2 Ab [Presence] in Specimen by Rapid immunoassay
- 49609-1 Vibrio sp DNA [Identifier] in Specimen by NAA with probe detection
- 49610-9 Streptococcus pyogenes DNA [Identifier] in Specimen by NAA with probe detection
- 49612-5 Salmonella sp DNA [Presence] in Specimen by NAA with probe detection
- 49614-1 Campylobacter sp DNA [Identifier] in Specimen by NAA with probe detection
- 49896-4 Human papilloma virus 16+18+31+33+35+39+45+51+52+56+58+59+68 DNA [Presence] in Specimen by NAA with probe detection
- 4993-2 Chlamydia trachomatis rRNA [Presence] in Specimen by Probe
- 5036-9 Streptococcus pyogenes rRNA [Presence] in Specimen by Probe
- 50412-6 Neisseria gonorrhoeae rRNA [Presence] in Specimen from Donor by NAA with probe detection
- 5060-9 Borrelia burgdorferi Ab [Units/volume] in Serum by Immunoassay
- 50690-7 Reagin Ab [Titer] in Serum by VDRL
- 50941-4 Mycobacterium sp [Presence] in Specimen by Organism specific culture
- 5124-3 Cytomegalovirus IgG Ab [Units/volume] in Serum or Plasma by Immunoassay
- 5126-8 Cytomegalovirus IgM Ab [Units/volume] in Serum or Plasma by Immunoassay
- 5157-3 Epstein Barr virus capsid IgG Ab [Units/volume] in Serum by Immunoassay
- 5159-9 Epstein Barr virus capsid IgM Ab [Units/volume] in Serum by Immunoassay
- 51656-7 Hepatitis C virus Ab Signal/Cutoff in Body fluid
- 5176-3 Helicobacter pylori IgG Ab [Units/volume] in Serum by Immunoassay
- 5187-0 Hepatitis B virus core Ab [Units/volume] in Serum by Immunoassay
- 51914-0 Hepatitis B virus core IgG+IgM Ab [Presence] in Serum
- 51916-5 Herpes simplex virus 1 IgG Ab [Presence] in Serum or Plasma by Immunoassay
- 51931-4 Rubella virus Ab [Units/volume] in Serum by Immunoassay
- 5195-3 Hepatitis B virus surface Ag [Presence] in Serum
- 5196-1 Hepatitis B virus surface Ag [Presence] in Serum or Plasma by Immunoassay
- 5206-8 Herpes simplex virus 1 IgG Ab [Units/volume] in Serum by Immunoassay
- 5209-2 Herpes simplex virus 2 IgG Ab [Units/volume] in Serum by Immunoassay
- 5213-4 Heterophile Ab [Presence] in Serum by Latex agglutination
- 5244-9 Measles virus IgG Ab [Units/volume] in Serum by Immunoassay
- 5255-5 Mycoplasma pneumoniae IgG Ab [Units/volume] in Serum by Immunoassay
- 5273-8 Parvovirus B19 IgG Ab [Units/volume] in Serum by Immunoassay
- 5274-6 Parvovirus B19 IgM Ab [Units/volume] in Serum by Immunoassay
- 52969-3 Methicillin resistant Staphylococcus aureus [Presence] in Nose by Organism specific culture
- 53251-5 Influenza virus B RNA [#/volume] (viral load) in Specimen by NAA with probe detection
- 53252-3 Parainfluenza virus 1 RNA [#/volume] (viral load) in Specimen by NAA with probe detection
- 53253-1 Parainfluenza virus 2 RNA [#/volume] (viral load) in Specimen by NAA with probe detection
- 53254-9 Parainfluenza virus 3 RNA [#/volume] (viral load) in Specimen by NAA with probe detection
- 5334-8 Rubella virus IgG Ab [Units/volume] in Serum or Plasma by Immunoassay
- 5370-2 Streptolysin O Ab [Units/volume] in Serum or Plasma
- 53776-1 Hepatitis A virus Ab [Interpretation] in Serum
- 5388-4 Toxoplasma gondii IgG Ab [Units/volume] in Serum or Plasma by Immunoassay

© 2026 Taquet M et al. *JAMA Psychiatry*.

- 5393-4 Treponema pallidum Ab [Presence] in Serum by Immunofluorescence
- 53947-8 Escherichia coli Stx1 and Stx2 toxin stx1 and stx2 and H7 flagellar fliC genes [Identifier] in Specimen by NAA with probe detection
- 5403-1 Varicella zoster virus IgG Ab [Units/volume] in Serum by Immunoassay
- 5404-9 Varicella zoster virus IgM Ab [Units/volume] in Serum by Immunoassay
- 54067-4 Clostridioides difficile toxin genes [Presence] in Stool by NAA with probe detection
- 54142-5 Candida sp DNA [Presence] in Vaginal fluid by Probe with signal amplification
- 54143-3 Gardnerella vaginalis DNA [Presence] in Vaginal fluid by Probe with signal amplification
- 54144-1 Trichomonas vaginalis DNA [Presence] in Vaginal fluid by Probe with signal amplification
- 546-2 Streptococcus.beta-hemolytic [Presence] in Throat by Organism specific culture
- 547-0 Streptococcus.beta-hemolytic [Presence] in Specimen by Organism specific culture
- 55465-9 Influenza virus A H1 2009 pandemic RNA [Presence] in Specimen by NAA with probe detection
- 56888-1 HIV 1+2 Ab+HIV1 p24 Ag [Presence] in Serum or Plasma by Immunoassay
- 57768-4 Campylobacter jejuni+Campylobacter coli Ag [Presence] in Stool
- 580-1 Fungus identified in Specimen by Culture
- 58452-4 Hepatitis B virus surface Ag [Units/volume] in Serum
- 586-8 Streptococcus agalactiae [Presence] in Specimen by Organism specific culture
- 5862-8 Influenza virus A Ag [Presence] in Specimen by Immunoassay
- 5866-9 Influenza virus B Ag [Presence] in Specimen by Immunoassay
- 5876-8 Respiratory syncytial virus Ag [Presence] in Specimen by Immunoassay
- 59263-4 Human papilloma virus 16 DNA [Presence] in Cervix by Probe with signal amplification
- 59264-2 Human papilloma virus 18 DNA [Presence] in Cervix by Probe with signal amplification
- 59420-0 Human papilloma virus 16+18+31+33+35+39+45+51+52+56+58+59+66+68 DNA [Presence] in Cervix by Probe with signal amplification
- 600-7 Bacteria identified in Blood by Culture
- 60269-8 Parainfluenza virus 1 RNA [Presence] in Isolate by NAA with probe detection
- 60271-4 Respiratory syncytial virus RNA [Presence] in Isolate by NAA with probe detection
- 60415-7 Parainfluenza virus 4 RNA [Presence] in Isolate by NAA with probe detection
- 60416-5 Parainfluenza virus 3 RNA [Presence] in Isolate by NAA with probe detection
- 60417-3 Parainfluenza virus 2 RNA [Presence] in Isolate by NAA with probe detection
- 60489-2 Streptococcus pyogenes DNA [Presence] in Throat by NAA with probe detection
- 60544-4 Giardia lamblia DNA [Presence] in Specimen by NAA with probe detection
- 60545-1 Cryptosporidium sp DNA [Presence] in Specimen by NAA with probe detection
- 611-4 Bacteria identified in Body fluid by Culture
- 61367-9 Clostridioides difficile DNA [Presence] in Specimen by NAA with probe detection
- 61371-1 Vibrio cholerae DNA [Presence] in Specimen by NAA with probe detection
- 61372-9 Human papilloma virus 16 DNA [Presence] in Specimen by NAA with probe detection
- 61373-7 Human papilloma virus 18 DNA [Presence] in Specimen by NAA with probe detection
- 61398-4 Escherichia coli DNA [Presence] in Specimen by NAA with probe detection
- 61404-0 Staphylococcus aureus DNA [Presence] in Specimen by NAA with probe detection
- 62423-9 Human coronavirus HKU1 RNA [Presence] in Specimen by NAA with probe detection
- 62460-1 Candida sp DNA [Presence] in Vaginal fluid by NAA with probe detection
- 62461-9 Trichomonas vaginalis DNA [Presence] in Vaginal fluid by NAA with probe detection
- 62462-7 Influenza virus A+B RNA [Presence] in Specimen by NAA with probe detection
- 625-4 Bacteria identified in Stool by Culture
- 626-2 Bacteria identified in Throat by Culture
- 62859-4 Rotavirus RNA [Presence] in Specimen by NAA with probe detection
- 630-4 Bacteria identified in Urine by Culture
- 6320-6 Borrelia burgdorferi IgG Ab [Presence] in Serum by Immunoblot
- 6321-4 Borrelia burgdorferi IgM Ab [Presence] in Serum by Immunoblot
- 634-6 Bacteria identified in Specimen by Aerobe culture
- 63427-9 Escherichia coli Stx1 toxin stx1 gene [Presence] in Specimen by NAA with probe detection
- 63428-7 Escherichia coli Stx2 toxin stx2 gene [Presence] in Specimen by NAA with probe detection
- 635-3 Bacteria identified in Specimen by Anaerobe culture
- 6356-0 Chlamydia trachomatis DNA [Presence] in Genital specimen by NAA with probe detection
- 6357-8 Chlamydia trachomatis DNA [Presence] in Urine by NAA with probe detection

© 2026 Taquet M et al. *JAMA Psychiatry*.

- 6362-8 Clostridioides difficile toxin A+B [Presence] in Stool by Cytotoxin tissue culture assay
- 6371-9 Cryptosporidium sp Ag [Presence] in Stool by Immunoassay
- 6396-6 Entamoeba histolytica DNA [Presence] in Specimen by NAA with probe detection
- 64084-7 Mycobacterium tuberculosis stimulated gamma interferon release by CD4+ T-cells [Units/volume] corrected for background in Blood
- 6410-5 Gardnerella vaginalis rRNA [Presence] in Genital specimen by Probe
- 6412-1 Giardia lamblia Ag [Presence] in Stool by Immunoassay
- 6462-6 Bacteria identified in Wound by Culture
- 6463-4 Bacteria identified in Specimen by Culture
- 6476-6 Mumps virus IgG Ab [Presence] in Serum by Immunoassay
- 6483-2 Mycoplasma pneumoniae rRNA [Presence] in Specimen by Probe
- 6516-9 Human papilloma virus rRNA [Presence] in Specimen by NAA with probe detection
- 6558-1 Streptococcus pyogenes Ag [Presence] in Specimen by Immunoassay
- 6559-9 Streptococcus pyogenes Ag [Presence] in Specimen by Immunofluorescence
- 6561-5 Treponema pallidum IgG Ab [Presence] in Serum
- 664-3 Microscopic observation [Identifier] in Specimen by Gram stain
- 68954-7 Streptococcus pyogenes rRNA [Presence] in Throat by Probe
- 69002-4 Human papilloma virus E6+E7 mRNA [Presence] in Cervix by NAA with probe detection
- 69562-7 Candida albicans DNA [Presence] in Vaginal fluid by NAA with probe detection
- 69563-5 Candida glabrata DNA [Presence] in Vaginal fluid by NAA with probe detection
- 69565-0 Atopobium vaginae DNA [Presence] in Vaginal fluid by NAA with probe detection
- 69566-8 Bacterial vaginosis associated bacterium 2 DNA [Presence] in Vaginal fluid by NAA with probe detection
- 69567-6 Megasphaera sp type 1 DNA [Presence] in Vaginal fluid by NAA with probe detection
- 69668-2 HIV 1 and 2 Ab [Identifier] in Serum or Plasma by Rapid immunoassay
- 698-1 Neisseria gonorrhoeae [Presence] in Specimen by Organism specific culture
- 69936-3 Gardnerella vaginalis DNA [Presence] in Specimen by NAA with probe detection
- 69937-1 Trichomonas vaginalis DNA [Presence] in Specimen by NAA with probe detection
- 69938-9 Astrovirus RNA [Presence] in Specimen by NAA with probe detection
- 70296-9 Plesiomonas shigelloides DNA [Presence] in Stool by NAA with probe detection
- 71429-5 Campylobacter sp DNA.diarrheagenic [Presence] in Stool by NAA with probe detection
- 71431-1 Human papilloma virus 31+33+35+39+45+51+52+56+58+59+66+68 DNA [Presence] in Cervix by NAA with probe detection
- 71772-8 Mitogen stimulated gamma interferon [Units/volume] in Blood
- 71773-6 Mycobacterium tuberculosis stimulated gamma interferon [Interpretation] in Blood Qualitative
- 71774-4 Mitogen stimulated gamma interferon [Units/volume] corrected for background in Blood
- 71776-9 Gamma interferon background [Units/volume] in Blood by Immunoassay
- 72111-8 Sapovirus RNA [Presence] in Specimen by NAA with probe detection
- 72356-9 Influenza virus A and B Ag [Identifier] in Specimen by Rapid immunoassay
- 72607-5 Streptococcus agalactiae [Presence] in Vag+Rectum by Organism specific culture
- 72885-7 Respiratory syncytial virus Ag [Presence] in Nasopharynx by Rapid immunoassay
- 72892-3 MRSA SCCmec and mecA genes panel - Nose by NAA with probe detection
- 75666-8 HIV 1+2 Ab and HIV1 p24 Ag [Identifier] in Serum, Plasma or Blood by Rapid immunoassay
- 76070-2 Adenovirus DNA [Presence] in Nasopharynx by NAA with probe detection
- 76078-5 Influenza virus A RNA [Presence] in Nasopharynx by NAA with probe detection
- 76080-1 Influenza virus B RNA [Presence] in Nasopharynx by NAA with probe detection
- 76084-3 Parainfluenza virus 1 RNA [Presence] in Nasopharynx by NAA with probe detection
- 76085-0 Parainfluenza virus 2 RNA [Presence] in Nasopharynx by NAA with probe detection
- 76086-8 Parainfluenza virus 3 RNA [Presence] in Nasopharynx by NAA with probe detection
- 76087-6 Parainfluenza virus 4 RNA [Presence] in Nasopharynx by NAA with probe detection
- 76089-2 Respiratory syncytial virus RNA [Presence] in Nasopharynx by NAA with probe detection
- 77026-3 Influenza virus A H1 RNA [Presence] in Nasopharynx by NAA with probe detection
- 77399-4 Human papilloma virus 16 DNA [Presence] in Cervix by NAA with probe detection
- 77400-0 Human papilloma virus 18 DNA [Presence] in Cervix by NAA with probe detection
- 7883-2 Epstein Barr virus nuclear IgG Ab [Presence] in Serum
- 7885-7 Epstein Barr virus capsid IgG Ab [Units/volume] in Serum

© 2026 Taquet M et al. *JAMA Psychiatry*.

- 7886-5 Epstein Barr virus capsid IgM Ab [Units/volume] in Serum
- 7905-3 Hepatitis B virus surface Ag [Presence] in Serum or Plasma by Neutralization test
- 79177-2 Clostridioides difficile glutamate dehydrogenase and toxins A+B [Presence] in Stool by Rapid immunoassay
- 7918-6 HIV 1+2 Ab [Presence] in Serum
- 7966-5 Mumps virus IgG Ab [Units/volume] in Serum
- 7974-9 Norovirus RNA [Presence] in Stool by NAA with probe detection
- 7993-9 Rhinovirus RNA [Presence] in Specimen by NAA with probe detection
- 8014-3 Rubella virus IgG Ab [Units/volume] in Serum
- 80348-6 Escherichia coli enteropathogenic eae gene [Presence] in Stool by NAA with non-probe detection
- 80349-4 Escherichia coli enteroaggregative pAA plasmid aggR+aatA genes [Presence] in Stool by NAA with non-probe detection
- 80350-2 Shigella species+EIEC invasion plasmid antigen H ipaH gene [Presence] in Stool by NAA with non-probe detection
- 80351-0 Escherichia coli enterotoxigenic ltA+st1a+st1b genes [Presence] in Stool by NAA with non-probe detection
- 80382-5 Influenza virus A Ag [Presence] in Upper respiratory specimen by Rapid immunoassay
- 80383-3 Influenza virus B Ag [Presence] in Upper respiratory specimen by Rapid immunoassay
- 80387-4 HIV 1+2 Ab [Presence] in Serum, Plasma or Blood by Rapid immunoassay
- 8047-3 Varicella zoster virus IgG Ab [Units/volume] in Serum
- 81658-7 Suspected organism [Identifier] in Specimen
- 82160-3 Adenovirus DNA [Presence] in Nasopharynx by NAA with non-probe detection
- 82161-1 Human coronavirus HKU1 RNA [Presence] in Nasopharynx by NAA with non-probe detection
- 82162-9 Human coronavirus NL63 RNA [Presence] in Nasopharynx by NAA with non-probe detection
- 82163-7 Human coronavirus 229E RNA [Presence] in Nasopharynx by NAA with non-probe detection
- 82164-5 Human coronavirus OC43 RNA [Presence] in Nasopharynx by NAA with non-probe detection
- 82165-2 Human metapneumovirus RNA [Presence] in Nasopharynx by NAA with non-probe detection
- 82166-0 Influenza virus A RNA [Presence] in Nasopharynx by NAA with non-probe detection
- 82167-8 Influenza virus A H1 RNA [Presence] in Nasopharynx by NAA with non-probe detection
- 82168-6 Influenza virus A H1 2009 pandemic RNA [Presence] in Nasopharynx by NAA with non-probe detection
- 82169-4 Influenza virus A H3 RNA [Presence] in Nasopharynx by NAA with non-probe detection
- 82170-2 Influenza virus B RNA [Presence] in Nasopharynx by NAA with non-probe detection
- 82171-0 Parainfluenza virus 1 RNA [Presence] in Nasopharynx by NAA with non-probe detection
- 82172-8 Parainfluenza virus 2 RNA [Presence] in Nasopharynx by NAA with non-probe detection
- 82173-6 Parainfluenza virus 3 RNA [Presence] in Nasopharynx by NAA with non-probe detection
- 82174-4 Parainfluenza virus 4 RNA [Presence] in Nasopharynx by NAA with non-probe detection
- 82175-1 Rhinovirus+Enterovirus RNA [Presence] in Nasopharynx by NAA with non-probe detection
- 82176-9 Respiratory syncytial virus RNA [Presence] in Nasopharynx by NAA with non-probe detection
- 82177-7 Mycoplasma pneumoniae DNA [Presence] in Nasopharynx by NAA with non-probe detection
- 82178-5 Chlamydophila pneumoniae DNA [Presence] in Nasopharynx by NAA with non-probe detection
- 82179-3 Bordetella pertussis.pertussis toxin promoter region [Presence] in Nasopharynx by NAA with non-probe detection
- 82196-7 Campylobacter coli+jejuni+upsaliensis DNA [Presence] in Stool by NAA with non-probe detection
- 82198-3 Plesiomonas shigelloides DNA [Presence] in Stool by NAA with non-probe detection
- 82199-1 Salmonella enterica+bongori DNA [Presence] in Stool by NAA with non-probe detection
- 82200-7 Vibrio cholerae+parahaemolyticus+vulnificus DNA [Presence] in Stool by NAA with non-probe detection
- 82201-5 Vibrio cholerae DNA [Presence] in Stool by NAA with non-probe detection
- 82202-3 Yersinia enterocolitica DNA [Presence] in Stool by NAA with non-probe detection

© 2026 Taquet M et al. *JAMA Psychiatry*.

- 82203-1 Escherichia coli Stx1 and Stx2 toxin stx1+stx2 genes [Presence] in Stool by NAA with non-probe detection
- 82205-6 Cryptosporidium sp DNA [Presence] in Stool by NAA with non-probe detection
- 82206-4 Cyclospora cayetanensis DNA [Presence] in Stool by NAA with non-probe detection
- 82207-2 Entamoeba histolytica DNA [Presence] in Stool by NAA with non-probe detection
- 82208-0 Giardia lamblia DNA [Presence] in Stool by NAA with non-probe detection
- 82209-8 Adenovirus 40+41 DNA [Presence] in Stool by NAA with non-probe detection
- 82210-6 Astrovirus subtypes 1-8 RNA [Presence] in Stool by NAA with non-probe detection
- 82211-4 Norovirus genogroup I+II RNA [Presence] in Stool by NAA with non-probe detection
- 82212-2 Rotavirus A RNA [Presence] in Stool by NAA with non-probe detection
- 82213-0 Sapovirus genogroups I+II+IV+V RNA [Presence] in Stool by NAA with non-probe detection
- 82509-1 Gamma interferon.negative control [Units/volume] in Blood
- 85477-8 Influenza virus A RNA [Presence] in Upper respiratory specimen by NAA with probe detection
- 85478-6 Influenza virus B RNA [Presence] in Upper respiratory specimen by NAA with probe detection
- 85479-4 Respiratory syncytial virus RNA [Presence] in Upper respiratory specimen by NAA with probe detection
- 87621-9 Bordetella parapertussis IS1001 DNA [Presence] in Nasopharynx by NAA with non-probe detection
- 88517-8 Mycobacterium tuberculosis stimulated gamma interferon release by CD4+ and CD8+ T-cells [Units/volume] corrected for background in Blood
- 88721-6 Rhinovirus+Enterovirus RNA [Presence] in Nasopharynx by NAA with probe detection
- 90435-9 Microorganism preliminary growth [Presence] in Blood by Aerobe culture
- 90437-5 Microorganism preliminary growth [Presence] in Blood by Anaerobe culture
- 6984-9 Beta lactamase.extended spectrum [Susceptibility]

eMethods 3. Definition of covariates

To reduce the effect of confounding on associations between a diagnosis of a variety of infections and a subsequent neurologic or psychiatric diagnosis, cohorts were matched for established or suspected risk factors for these infections, vaccination status and for concurrent or past use of antidepressants and antipsychotics that are associated with both infection risk and subsequence psychiatric and neurologic conditions. Because a history of the psychiatric and neurologic disorders can be associated with subsequent onset of other disorders, each of the disorders was also included as a covariate. The following confounding factors were therefore included (with ICD-10/CDC codes in brackets):

1) **Age** at time of index event
2) **Sex** coded as female, male or unknown
3) **Race** encoded as 6 separate dichotomous variables: White (2106-3), Black or African American (2054-5), American Indian or Alaska Native (1002-5), Asian (2089-9), Native Hawaiian or Other Pacific Islander (2076-8), Other Race (2131-1) or Unknown Race (UKN)
4) **Ethnicity** encoded as Hispanic or Latino (2135-2), Not Hispanic or Latino (2186-5), or Unknown Ethnicity (UN)
5) **Socioeconomic** encoded as the ICD-10 code for Problems related to housing and economic circumstances (Z59)
6) **Relationship status** encoded as Never Married (S), Married (M), Divorced (D), Widowed (W), Legally Separated (L), Domestic partner (T)
7) **Obesity** encoded as Overweight and obesity (E66)
8) **Hypertension** coded as Hypertensive diseases (I10-I1A)
9) **Diabetes mellitus** encoded as 2 dichotomous variables: Type 1 diabetes mellitus (E10) and Type 2 diabetes mellitus (E11).
10) **Chronic lower respiratory diseases** encoded by each sub-category of the corresponding ICD-10 group: Bronchitis, not specified as acute or chronic (J40), Simple and mucopurulent chronic bronchitis (J41), Unspecified chronic bronchitis (J42), Emphysema (J43), Other chronic obstructive pulmonary disease (J44), Asthma (J45), Bronchiectasis (J47).
11) **Nicotine dependence** encoded as the corresponding ICD-10 diagnosis (F17.2).

© 2026 Taquet M et al. *JAMA Psychiatry*.

12) **Substance use disorders** encoded as the ICD-10 code for mental and behavioural disorders due to psychoactive substance use (F10-F19).
13) **Psychotic disorders** encoded as the ICD-10 code for schizophrenia, schizotypal, delusional, and other non-mood psychotic disorders (F20-F29).
14) **Mood disorders** encoded as a single variable (as well as individual codes, see below) with any of the ICD-10 code for mood [affective] disorders (F30-F39).
15) **Anxiety disorders** encoded as the ICD-10 code for anxiety, dissociative, stress-related, somatoform and other nonpsychotic mental disorders (F40-F48)
16) **Heart diseases** encoded as 2 categorical variables: Ischaemic heart disease (I20-I25) and Other forms of heart disease (I30-I5A).
17) **Chronic kidney disease** encoded as 2 dichotomous variables: Chronic kidney disease (N18) and Hypertensive chronic kidney disease (I12).
18) **Chronic liver disease** encoded as 8 categorical variables: Alcoholic liver disease (K70), Hepatic failure, not elsewhere classified (K72), Chronic hepatitis, not elsewhere classified (K73), Fibrosis and cirrhosis of liver (K74), Fatty (change of) liver, not elsewhere classified (K76.0), Chronic passive congestion of liver (K76.1), Portal hypertension (K76.6), Other specified diseases of liver (K76.8).
19) **Stroke** encoded as the dichotomous variable Cerebral infarction (I63) .
20) **Dementia** encoded as 6 dichotomous variables: Vascular dementia (F01), Dementia in other diseases classified elsewhere (F02), Unspecified dementia (F03), Alzheimer's disease (G30), Frontotemporal dementia (G31.0), and Neurocognitive disorder with Lewy bodies (G31.83).
21) **Neoplasm and haematological cancer** in particular encoded as 2 dichotomous variables: Neoplasms (C00-D49) and Malignant neoplasms of lymphoid, hematopoietic and related tissue (C81-C96).
22) **Rheumatoid arthritis** encoded as 2 dichotomous variables: Rheumatoid arthritis with rheumatoid factor (M05) and Other rheumatoid arthritis (M06).
23) **Systemic lupus erythematosus** encoded as a dichotomous variable corresponding ICD-10 code (M32).
24) **Disorders involving an immune mechanism** encoded as a dichotomous variable "Certain disorders involving the immune mechanism" (D80-D89).
25) **Dermatitis and eczema** encoded as a dichotomous variable corresponding ICD-10 code (L20-30).
26) I**nsomnia** encoded as 2 dichotomous variables: Insomnia (G47.0) and Insomnia not due to a substance or known physiological condition (F51.0)
27) **Intracranial haemorrhage** encoded as 3 dichotomous variables: Nontraumatic subarachnoid haemorrhage (I60), Nontraumatic intracerebral haemorrhage (I61) and Other and unspecified nontraumatic intracranial haemorrhage (I62).
28) **Parkinsonism encoded** as 2 dichotomous variables: Parkinson's disease (G20) and Secondary parkinsonism (G21).
29) **Central neurologic disorder** encoded as a dichotomous variable Multiple sclerosis (G35).
30) **Peripheral** neurologic disorders encoded as 3 dichotomous variables: Guillan-Barre syndrome (G61.0), Nerve, nerve root and plexus disorders (G50-G59) and Diseases of myoneural junction and muscle (G70-G73).
31) **Encephalopathy and encephalitis** encoded as 5 dichotomous variables: Encephalopathy, unspecified (G93.40), Encephalitis, myelitis and encephalomyelitis (G04), Encephalitis, myelitis and encephalomyelitis in diseases classified elsewhere (G05), Unspecified viral encephalitis (A86) and Other specified viral encephalitis (A85.8).
32) **Cognitive impairment and mental disorders** encoded as 3 dichotomous variables: Mild cognitive impairment of uncertain or unknown etiology (G31.84), Dyslexia and other symbolic dysfunctions, not elsewhere classified (R48), Other specified mental disorders due to known physiological condition (F06.8).
33) **Altered mental state** encoded as 3 dichotomous variables: Delirium due to known physiological condition (F05), Somnolence, stupor, coma (R40), Other symptoms and signs involving cognitive functions and awareness (R41).
34) **Epilepsy and seizures** encoded as 2 dichotomous variables: Epilepsy and recurrent seizures (G40) and Convulsions, not elsewhere classified (R56).
35) **Antipsychotics** as a class, encoded as VA Class CN700
36) **Antidepressants** as a class, encoded as VA Class CN600
37) **Vaccinations**, including:
    - Bacterial vaccines as a class, encoded as J07A
    - Varicella zoster vaccines as a class, encoded as J07BK
    - Flu
    - TDAP

© 2026 Taquet M et al. *JAMA Psychiatry.*

- HepA
- HepB
- HIB
- DTAP
- POLIO
- Td
- PneumoPPV
- PneumoPCV
- MMR
- Varicella
- Zoster
- Meningitis
- HPV
- Rotavirus

Each individual code was considered a confounding factor in and of itself, so that matching was achieved for each of them individually. For instance, matching was achieved for each subcategory (and not just for the whole category) of chronic lower respiratory diseases. For variables representing diagnoses, socioeconomic deprivation, and medications, the confounder was considered present if the corresponding code was recorded at least once in a patient's health record before the index event.

Data on race, ethnicity, and sex could be recorded as unknown and this was considered as a category of its own so that little difference in the number of people with unknown race/ethnicity/sex would be present in matched cohorts. This appeared to be a better approach than excluding those with unknown race/ethnicity/sex since this might affect the generalisability of the results (e.g. by excluding individuals who prefer not to disclose their ethnicities).

The primary analysis was stratified by age group (≤ 18, 19-44, 45-64, ≥ 65 years-old) and age was also included as a continuous covariate within each stratum, thus providing additional controlling for age.

eMethods 4. Definition of outcomes

All outcomes were defined as a diagnosis recorded in the patient's electronic health record from the day following the index event until the end of follow-up. As mentioned in the manuscript, for chronic illnesses, only first diagnoses were counted (i.e. patients with the diagnosis before the index event were excluded from the survival analysis). For diagnoses that can recur or relapse, we separately estimated the incidence of first diagnosis and the incidence of any diagnosis, but we focus on first diagnosis in the main manuscript (while presenting the results for any diagnosis in this appendix) given that any diagnosis might include re-coding of previous diagnoses. For outcomes that do not tend to recur after they have resolved (e.g., Guillain-Barré syndrome), we estimated the incidence of any diagnoses.

Specifically, the following ICD-10 codes (with the ICD-10 labels in brackets) were used to define outcomes:

1) **Anxiety disorder** (first diagnosis): F40-F48 (Anxiety, dissociative, stress-related, somatoform and other nonpsychotic mental disorders)
2) **Cognitive deficit** (first diagnosis): F01 (Vascular dementia), F02 (Dementia in other diseases classified elsewhere), F03 (Unspecified dementia), F05 (Delirium due to known physiological condition), F06.8 (Other specified mental disorders due to known physiological condition), G30 (Alzheimer's disease), G31.0 (Frontotemporal dementia), G31.83 (Neurocognitive disorder with Lewy bodies), G31.84 (Mild cognitive impairment of uncertain or unknown etiology), G93.40 (Encephalopathy, unspecified), R40 (Somnolence, stupor and coma), R41 (Other symptoms and signs involving cognitive functions and awareness), R48 (Dyslexia and other symbolic dysfunctions, not elsewhere classified). These terms which mix actual diagnoses and symptoms were used to capture the range of diagnostic codes that patients presenting with 'brain fog' might receive. They are the same terms as used in our previous study of long-COVID features. This outcome overlaps with the next one ('Dementia') by including all dementia codes. The reason for including dementia diagnoses in 'cognitive deficit' is to account for possible misdiagnoses of brain fog as dementia, and to more fully represent any cognitive deficit that can occur after infection.

3) **Dementia** (first diagnosis): F01 (Vascular dementia), F02 (Dementia in other diseases classified elsewhere), F03 (Unspecified dementia), G30 (Alzheimer's disease), G31.0 (Frontotemporal dementia), G31.83 (Neurocognitive disorder with Lewy bodies)
4) **Encephalitis** (any diagnosis): G04 (Encephalitis, myelitis and encephalomyelitis), G05 (Encephalitis, myelitis and encephalomyelitis in diseases classified elsewhere), A86 (Unspecified viral encephalitis), or A85.8 (Other specified viral encephalitis)
5) **Epilepsy/seizures** (first diagnosis): G40 (Epilepsy and recurrent seizures), R56 (Convulsions, not elsewhere classified)
6) **Insomnia (**first diagnosis): F51.0 (Insomnia not due to a substance or known physiological condition) or G47.0 (Insomnia)
7) **Intracranial haemorrhage** (first diagnosis): I60 (non-traumatic subarachnoid haemorrhage), I61 (non-traumatic intracerebral haemorrhage), and I62 (other and unspecified non-traumatic intracranial haemorrhage)
8) **Ischaemic stroke** (first diagnosis): I63 (cerebral infarction)
9) **Mood disorder** (first diagnosis): F30-F39 (Mood disorders). ICD-10 codes representing remission (e.g. F32.4 - Major depressive disorder, single episode, in partial remission) were excluded.
10) **Myoneural junction/muscle disease** (first diagnosis): G70-G73 (Diseases of myoneural junction and muscle) – these disorders are often called neuromuscular, but we use the ICD-10 term
11) **Nerve/nerve root/plexus disorders** (any diagnosis): G50-G59 (Nerve, nerve root and plexus disorders)
12) **Multiple Sclerosis** (first diagnosis): G35 (Multiple Sclerosis)
13) **Parkinsonism** (first diagnosis): G20 (Parkinson's disease) or G21 (Secondary parkinsonism)
14) **Psychotic disorder** (first diagnosis): F20-F29 (Schizophrenia, schizotypal, delusional, and other non-mood psychotic disorders)

eMethods 5. Details on statistical analyses

Implementation details of propensity score matching
In propensity score matching, the propensity score was calculated using a logistic regression (implemented by the function LogisticRegression of the scikit-learn package in Python 3.7) including each of the covariates mentioned above. To eliminate the influence of ordering of records, the order of the records in the covariate matrix were randomised before matching. A greedy nearest neighbour matching approach was used with a calliper distance of 0.1 pooled standard deviations of the logit of the propensity score.

Estimation of RMTL ratios
RMTL ratios and their 95% CI alongside the p-value for the null hypothesis that the ratio equals 1, were estimated using survRM2 package version 1.0.4.

Assessment of the most recorded infectious/non-infectious events on admission in the cohorts
For the infectious cohorts, we included all diagnostic codes reflecting ≥5% of infection-related codes recorded on the day of admission, grouped by body system. For the non-infectious cohorts, we based our selection on the most frequent principal diagnoses identified in the 2018 Healthcare Cost and Utilization Project (HCUP) Statistical Brief by McDermott and Roemer, which reports the most frequent principal diagnoses for inpatient stays in the United States during the study period covered.[1] From this list we assessed the 16 non-infectious categories (excluding those corresponding to infections) and additionally included 'pregnancy, childbirth, and the puerperium' as an important non-infectious cause of hospitalisation that was explicitly excluded from the HCUP list. In total 17 non-infectious codes were evaluated for each age group.

Assessment of marginal means
Marginal means were obtained using the emmeans package v. 2.0.0 in R. We report marginal means (over all infections and disorders) for the overall age effect and marginal means (over all infections) for the age effect within each disorder (and conversely for the age effect within each infection). We used sum-to-zero coding so the age coefficient represents the age contrast relative to the average effect (i.e. average log-RMTL ratio or average absolute risk difference) across ages.

Details on network meta-analysis
Since each infection was compared to all other infections, we borrowed tools from the network meta-analysis literature to summarise results from these 45 comparisons. Specifically, we were interested in assessing the degree to which an infection is specifically associated with a disorder (i.e. more likely than other infections to be

© 2026 Taquet M et al. *JAMA Psychiatry.*

associated with an increased risk of the disorder). This can be quantified by the P-score often used in network meta-analysis.

We estimated the P-score for each infection and each outcome by fitting a random-effects frequentist network meta-analysis using netmeta in R (version 2.9.0) on the log-RMTL ratio. One condition for the P-score to be meaningful is that there is no inconsistency in the network. Inconsistency would not be present if the comparisons were unmatched (since the exact same cohorts would be used across comparisons). However, inconsistencies might occur because after matching, cohorts can be different between comparisons. For instance, the RTI cohort matched to the meningitides cohort may be somewhat different from the RTI cohort matched to the STI cohort. Global inconsistency was assessed using Q-tests (as returned by netmeta).

Of note, the network meta-analysis model can also provide estimates of effects among all exposures. However, these estimates were not used in our analysis since comparisons are not independent. For instance, when comparing RTI to meningitis and RTI to STI, the same patients can be included in both RTI cohorts (unlike many conventional network meta-analyses wherein comparisons come from independent studies). Ignoring this dependence between comparisons would be equivalent to artificially inflating sample size and thus leading to invalid standard errors. By contrast, the relative ranking of the exposures (P-scores) is not expected to be affected by this limitation and is therefore reported.

Details of risk factor profile clustering
The correlation matrix between risk factor profiles for each disorder was transformed into a distance matrix using the arccosine transformation. Hierarchical clustering was then applied on this distance matrix using the function hclust in R with Ward's method. The 'optimal' number of clusters was determined using a dynamic tree cutting approach as implemented with cutreeDynamic function of the package dynamicTreeCut v. 1.63 in R (setting the maximum cut height to 1).

eReferences

1 McDermott KW, Roemer M. Most Frequent Principal Diagnoses for Inpatient Stays in U.S. Hospitals, 2018. *HCUP Statistical Brief* 2021; **#277**. https://hcup-us.ahrq.gov/reports/statbriefs/sb277-Top-Reasons-Hospital-Stays-2018.pdf.

© 2026 Taquet M et al. *JAMA Psychiatry*.

Supplementary Results

eResults 1. Main infections by body system and main non-infective events on the day of admission

1) Bone infections
a. Children
- Osteomyelitis (M86): 100%

b. Younger adults
- Osteomyelitis (M86): 100%

c. Middle-aged adults
- Osteomyelitis (M86): 100%

d. Older adults
- Osteomyelitis (M86): 100%

2) Cardiac infections
a. Children
- Endocarditis, valve unspecified (I38): 49%
- Acute/subacute infective endocarditis (I33.0): 34%
- Infective myocarditis (I40.0): 15%
- Infective pericarditis (I30.1): 7%

b. Younger adults
- Endocarditis, valve unspecified (I38): 56%
- Acute/subacute infective endocarditis (I33.0): 50%

c. Middle-aged adults
- Endocarditis, valve unspecified (I38): 61%
- Acute/subacute infective endocarditis (I33.0): 43%

d. Older adults
- Endocarditis, valve unspecified (I38): 67%
- Acute/subacute infective endocarditis (I33.0): 37%

3) GI infections
a. Children
- Viral/other specified intestinal infections (A08): 62%
- Other bacterial intestinal infectiouns (A04): 25%
- Unspecified infectious gastroenteritis/colitis (A09): 12%

b. Younger adults
- Other bacterial intestinal infections (A04): 56%
- Viral/other specified intestinal infections (A08): 28%
- Unspecified infectious gastroenteritis/colitis (A09): 14%

c. Middle-aged adults
- Other bacterial intestinal infections (A04): 66%
- Viral/other specified intestinal infections (A08): 20%
- Unspecified infectious gastroenteritis/colitis (A09): 13%

d. Older adults
- Other bacterial intestinal infections (A04): 71%
- Viral/other specified intestinal infections (A08): 17%
- Unspecified infectious gastroenteritis/colitis (A09): 13%

4) Hepatitidies
a. Children
- Other acute viral hepatitis (B17): 83%
- Acute hepatitis A (B15): 14%
- Acute hepatitis B (B16): 5%

b. Younger adults
- Other acute viral hepatitis (B17): 65%
- Acute hepatitis A (B15): 22%

© 2026 Taquet M et al. *JAMA Psychiatry*.

- Acute hepatitis B (B16): 18%

c. Middle-aged adults
- Other acute viral hepatitis (B17): 56%
- Acute hepatitis A (B15): 30%
- Acute hepatitis B (B16): 17%

d. Older adults
- Other acute viral hepatitis (B17): 56%
- Acute hepatitis A (B15): 27%
- Acute hepatitis B (B16): 22%

5) Inf. Encephalitidies

a. Children
- Herpesviral encephalitis (B00.4): 68%
- Enteroviral encephalitis (A85.0): 20%
- Other specified viral encephalitis (A85.8): 18%

b. Younger adults
- Herpesviral encephalitis (B00.4): 83%
- Other specified viral encephalitis (A85.8): 15%
- Enteroviral encephalitis (A85.0): 8%

c. Middle-aged adults
- Herpesviral encephalitis (B00.4): 87%
- Other specified viral encephalitis (A85.8): 13%

d. Older adults
- Herpesviral encephalitis (B00.4): 89%
- Other specified viral encephalitis (A85.8): 10%

6) Meningitidies

a. Children
- Viral meningitis (A87): 60%
- Bacterial meningitis, not elsewhere classified (G00): 40%

b. Younger adults
- Viral meningitis (A87): 64%
- Bacterial meningitis, not elsewhere classified (G00): 34%

c. Middle-aged adults
- Bacterial meningitis, not elsewhere classified (G00): 56%
- Viral meningitis (A87): 40%

d. Older adults
- Bacterial meningitis, not elsewhere classified (G00): 64%
- Viral meningitis (A87): 33%

7) RTI

a. Children
- Acute bronchiolitis due to RSV (J21.0): 35%
- Bronchiolitis due to other specified organisms (J21.8): 28%
- Influenza with respiratory manifestations (J10.1): 11%
- Streptococcal pharyngitis (J02.0): 11%

b. Younger adults
- Influenza with respiratory manifestations (J10.1): 22%
- Streptococcal pharyngitis (J02.0): 13%
- Pseudomonas Pneumonia (J15.1): 8%
- Other Gram -ve pneumonias (J15.6): 7%
- Strept. Pneumoniae pneumonia (J13): 6%

c. Middle-aged adults

- Influenza with respiratory manifestations (J10.1): 21%
- Other Gram -ve pneumonias (J15.6): 11%
- Strept. Pneumoniae pneumonia (J13): 9%
- Streptococcal pharyngitis (J02.0): 8%
- Pseudomonas Pneumonia (J15.1): 7%
- Klebsiella pneumoniae pneumonia (J15.0): 5%
- Influenza with pneumonia (J10.0): 5%
- Pneumonia due to other specified organisms (J16.8): 5%

d. Older adults
- Influenza with respiratory manifestations (J10.1): 26%
- Other Gram -ve pneumonias (J15.6): 13%
- Pseudomonas Pneumonia (J15.1): 9%
- Pneumonia due to MRSA (J15.212): 7%
- Influenza with pneumonia (J10.0): 6%
- Strept. Pneumoniae pneumonia (J13): 6%
- Pneumonia due to MSSA (J15.211): 5%
- Klebsiella pneumoniae pneumonia (J15.0): 5%
- Pneumonia due to other specified organisms (J16.8): 5%
- Acute bronchitis due to other specified organisms (J20.8): 5%

8) STI

a. Children
- Other sexually transmitted chlamydial diseases (A56): 33%
- Anogenital herpesviral infections (A60): 21%
- Gonococcal infection (A54): 18%
- Trichomoniasis (A59): 16%
- Other STIs, not elsewhere classified (A63): 9%
- HIV (B20): 8%

b. Younger adults
- Anogenital herpesviral infections (A60): 35%
- HIV (B20): 28%
- Other STIs, not elsewhere classified (A63): 14%
- Trichomoniasis (A59): 13%
- Other sexually transmitted chlamydial diseases (A56): 6%

c. Middle-aged adults
- HIV (B20): 70%
- Anogenital herpesviral infections (A60): 11%
- Other STIs, not elsewhere classified (A63): 10%
- Other and unspecified syphilis (A53): 5%
- Trichomoniasis (A59): 5%

d. Older adults
- HIV (B20): 52%
- Anogenital herpesviral infections (A60): 20%
- Other STIs, not elsewhere classified (A63): 12%
- Other and unspecified syphilis (A53): 11%

9) Skin infections

a. Children
- Cellulitis and acute lymphangitis (L03): 56%
- Cutaneous abscess, furuncle and carbuncle (L02): 28%
- Other local skin/subcutaneous infections (L08): 8%
- Skin/nail candidiasis (B37.2): 7%
- Dermatophytosis (B35): 5%

© 2026 Taquet M et al. *JAMA Psychiatry*.

b. Younger adults
- Cellulitis and acute lymphangitis (L03): 64%
- Cutaneous abscess, furuncle and carbuncle (L02): 34%
- Other local skin/subcutaneous infections (L08): 11%

c. Middle-aged adults
- Cellulitis and acute lymphangitis (L03): 66%
- Cutaneous abscess, furuncle and carbuncle (L02): 27%
- Other local skin/subcutaneous infections (L08): 12%
- Dermatophytosis (B35): 6%

d. Older adults
- Cellulitis and acute lymphangitis (L03): 64%
- Cutaneous abscess, furuncle and carbuncle (L02): 19%
- Other local skin/subcutaneous infections (L08): 10%
- Dermatophytosis (B35): 7%
- Zoster (B02): 7%

10) UTI

a. Children
- UTI, site not specified (N39.0): 88%
- Acute pyelonephritis (N10): 17%

b. Younger adults
- UTI, site not specified (N39.0): 91%
- Acute pyelonephritis (N10): 12%

c. Middle-aged adults
- UTI, site not specified (N39.0): 96%
- Acute pyelonephritis (N10): 6%

d. Older adults
- UTI, site not specified (N39.0): 98%
- Acute pyelonephritis (N10): 0%

11) Non-infective

a. Children
- Injury and poisoning (S00-T88): 13%
- Depressive disorders (F32-F33): 7%
- COPD, asthma and bronchiectasis (J40-J4A): 6%
- Fluid and electrolyte disorders (E86-E87): 2%
- Respiratory failure/insufficiency/arrest (J96, R06.89, R09.2): 2%
- Cardiac dysrhythmias (I47-I49): 1%
- Diabetes (E08-E13): 1%
- Schizophrenia spectrum and other psychotic disorders (F20-F29): 1%
- Pregnancy, childbirth, and the puerperium (O00-O9A): 1%
- Coronary atherosclerosis and other heart disease (I25): 0%
- Heart failure (I50): 0%
- Acute myocardial infarction (I21-I22): 0%
- Cerebral infarction (I63): 0%
- Spondylopathies / spondyloarthropathy (M45-M49): 0%
- Acute and unspecified renal failure (N17, N19): 0%
- Biliary tract diseases (K80-K83): 0%
- Osteoarthritis (M15-M19): 0%

b. Younger adults
- Pregnancy, childbirth, and the puerperium (O00-O9A): 15%
- Injury and poisoning (S00-T88): 10%
- Depressive disorders (F32-F33): 7%
- Cardiac dysrhythmias (I47-I49): 3%

© 2026 Taquet M et al. *JAMA Psychiatry*.

- COPD, asthma and bronchiectasis (J40-J4A): 3%
- Fluid and electrolyte disorders (E86-E87): 3%
- Diabetes (E08-E13): 2%
- Schizophrenia spectrum and other psychotic disorders (F20-F29): 2%
- Coronary atherosclerosis and other heart disease (I25): 1%
- Heart failure (I50): 1%
- Acute myocardial infarction (I21-I22): 1%
- Cerebral infarction (I63): 1%
- Spondylopathies / spondyloarthropathy (M45-M49): 1%
- Respiratory failure/insufficiency/arrest (J96, R06.89, R09.2): 1%
- Acute and unspecified renal failure (N17, N19): 1%
- Biliary tract diseases (K80-K83): 1%
- Osteoarthritis (M15-M19): 0%

c. Middle-aged adults
- Injury and poisoning (S00-T88): 8%
- Diabetes (E08-E13): 8%
- Cardiac dysrhythmias (I47-I49): 7%
- Coronary atherosclerosis and other heart disease (I25): 7%
- Depressive disorders (F32-F33): 6%
- COPD, asthma and bronchiectasis (J40-J4A): 5%
- Fluid and electrolyte disorders (E86-E87): 4%
- Acute myocardial infarction (I21-I22): 4%
- Heart failure (I50): 3%
- Osteoarthritis (M15-M19): 3%
- Cerebral infarction (I63): 2%
- Spondylopathies / spondyloarthropathy (M45-M49): 2%
- Respiratory failure/insufficiency/arrest (J96, R06.89, R09.2): 1%
- Acute and unspecified renal failure (N17, N19): 1%
- Biliary tract diseases (K80-K83): 1%
- Schizophrenia spectrum and other psychotic disorders (F20-F29): 1%
- Pregnancy, childbirth, and the puerperium (O00-O9A): 0%

d. Older adults
- Coronary atherosclerosis and other heart disease (I25): 12%
- Cardiac dysrhythmias (I47-I49): 12%
- Diabetes (E08-E13): 11%
- Injury and poisoning (S00-T88): 8%
- COPD, asthma and bronchiectasis (J40-J4A): 6%
- Heart failure (I50): 5%
- Osteoarthritis (M15-M19): 5%
- Fluid and electrolyte disorders (E86-E87): 5%
- Acute myocardial infarction (I21-I22): 4%
- Cerebral infarction (I63): 3%
- Depressive disorders (F32-F33): 3%
- Spondylopathies / spondyloarthropathy (M45-M49): 2%
- Respiratory failure/insufficiency/arrest (J96, R06.89, R09.2): 2%
- Acute and unspecified renal failure (N17, N19): 2%
- Biliary tract diseases (K80-K83): 1%
- Schizophrenia spectrum and other psychotic disorders (F20-F29): 0%
- Pregnancy, childbirth, and the puerperium (O00-O9A): 0%

© 2026 Taquet M et al. *JAMA Psychiatry*.

eResults 2. Repeated exposures

Some people may enter more than one cohort if they experienced multiple qualifying exposures during the study period. The privacy constraints within TriNetX prevent identifying individuals across cohorts. Two concerns follow: (i) standard errors might be underestimated (since robust variance cannot be estimated), and (ii) if a second exposure occurs during follow-up, risks of neurologic or psychiatric outcomes from one exposure could be correlated with those from the other. We gauge the size of these issues by the observed overlap between cohorts. Fewer than 0.05% of those hospitalised for UTI or RTI (the two most common infections) also had a non-infectious hospitalisation in the study period, and only 6% of those hospitalised with a UTI were ever hospitalised with an RTI (not necessarily within two years of one another). This limited overlap implies that repeated exposure during follow-up is rare, any induced correlation is small, precision inflation is unlikely to affect inference (most p-values are many orders of magnitude below 0.05), and point estimates are likely to be unaffected.

© 2026 Taquet M et al. *JAMA Psychiatry*.

Supplementary figures

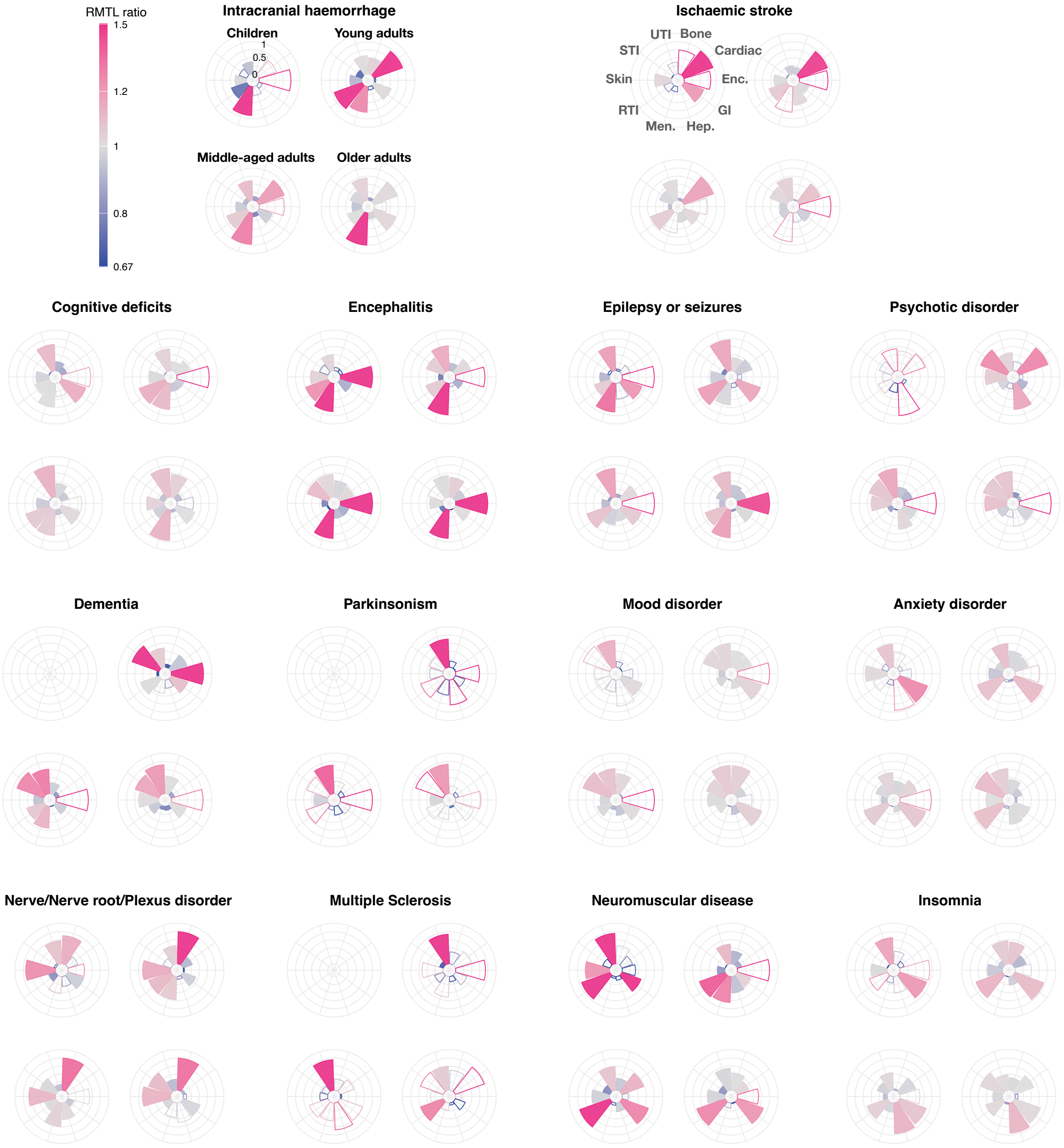

eFigure 1. Summary of the head-to-head comparisons between hospitalised infections, stratified by age group. Each panel shows the risk of one disorder following each infection. The top left plot corresponds to children, the top right to young adults, the bottom left to middle-aged adults, and the bottom right to older adults. The colour of the spoke encodes the median RMTL ratio for the comparison between the corresponding infection and all other infections. It is kept empty if the risk did not differ from that seen in the control group (i.e. if the RMTL ratio for the comparison with the control group was not significantly different from 1). The length of each spoke encodes the P-score (graduations provided in the top left panel) representing the specificity of the infection for the disorder. Absent spokes indicate that the outcome was not observed in that age group. Bone=Bone infections; Enc.=Infectious encephalitides; GI=Gastrointestinal infections; Hep.=Hepatitides; Men.=Meningitides; RTI=Respiratory tract infections; Skin=Skin infections; STI=Sexually-transmitted infections; UTI=Urinary tract infections.

© 2026 Taquet M et al. *JAMA Psychiatry*.

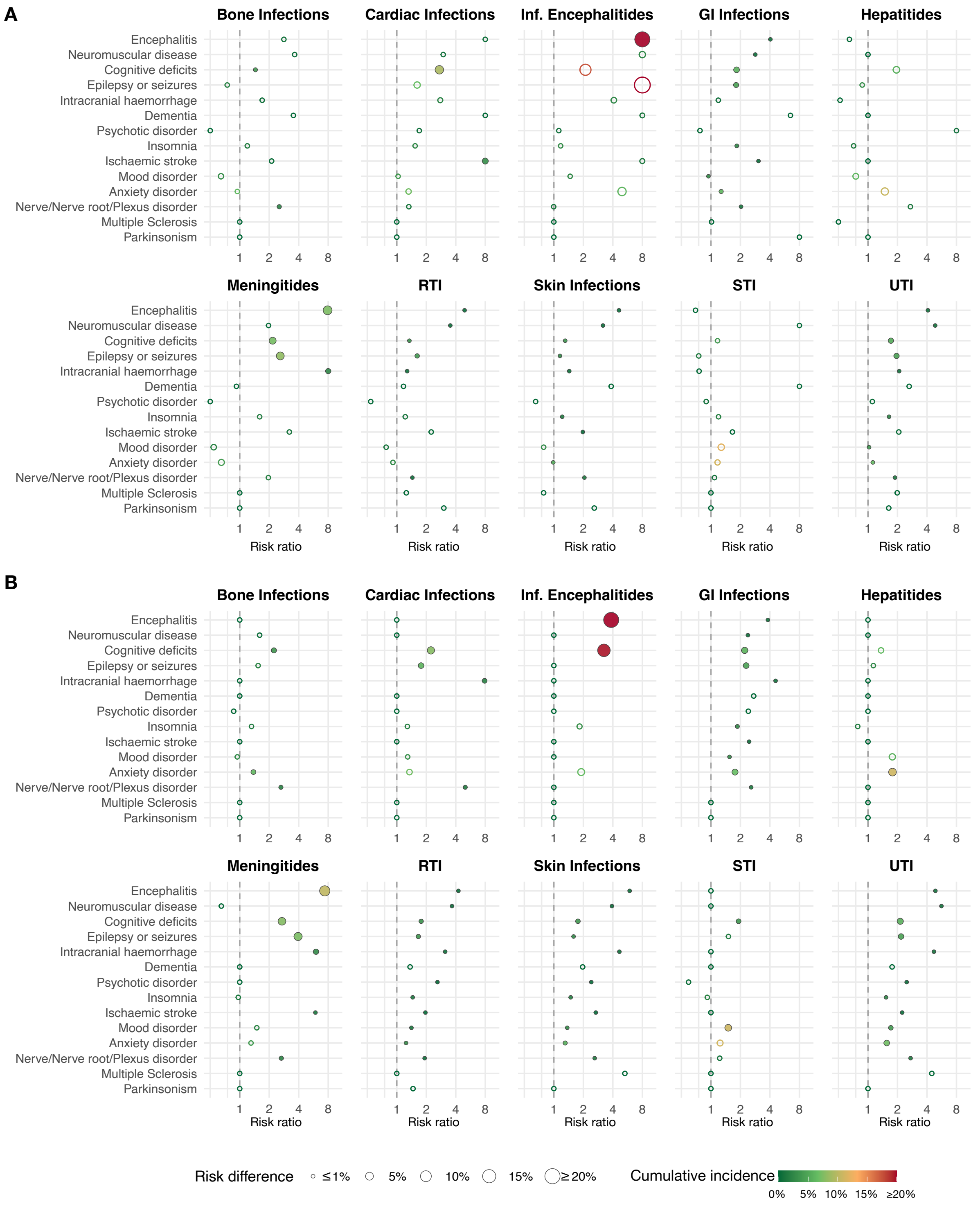

eFigure 2. Comparison of the risk of each outcome between hospitalised infections and (A) hospitalisations for other causes, (B) the general population in children**.** The location of each dot on the x-axis represents the risk ratio of the disorder occurring within 2 years; its size represents the absolute risk difference; and its colour represents the 2-year risk of the condition after the infection. Dots that are filled represent statistically significant risks after Bonferroni correction, whereas empty dots denote no statistical significance. Inf=Infectious; GI=Gastrointestinal. Other abbreviations as per eFigure 1.

© 2026 Taquet M et al. *JAMA Psychiatry.*

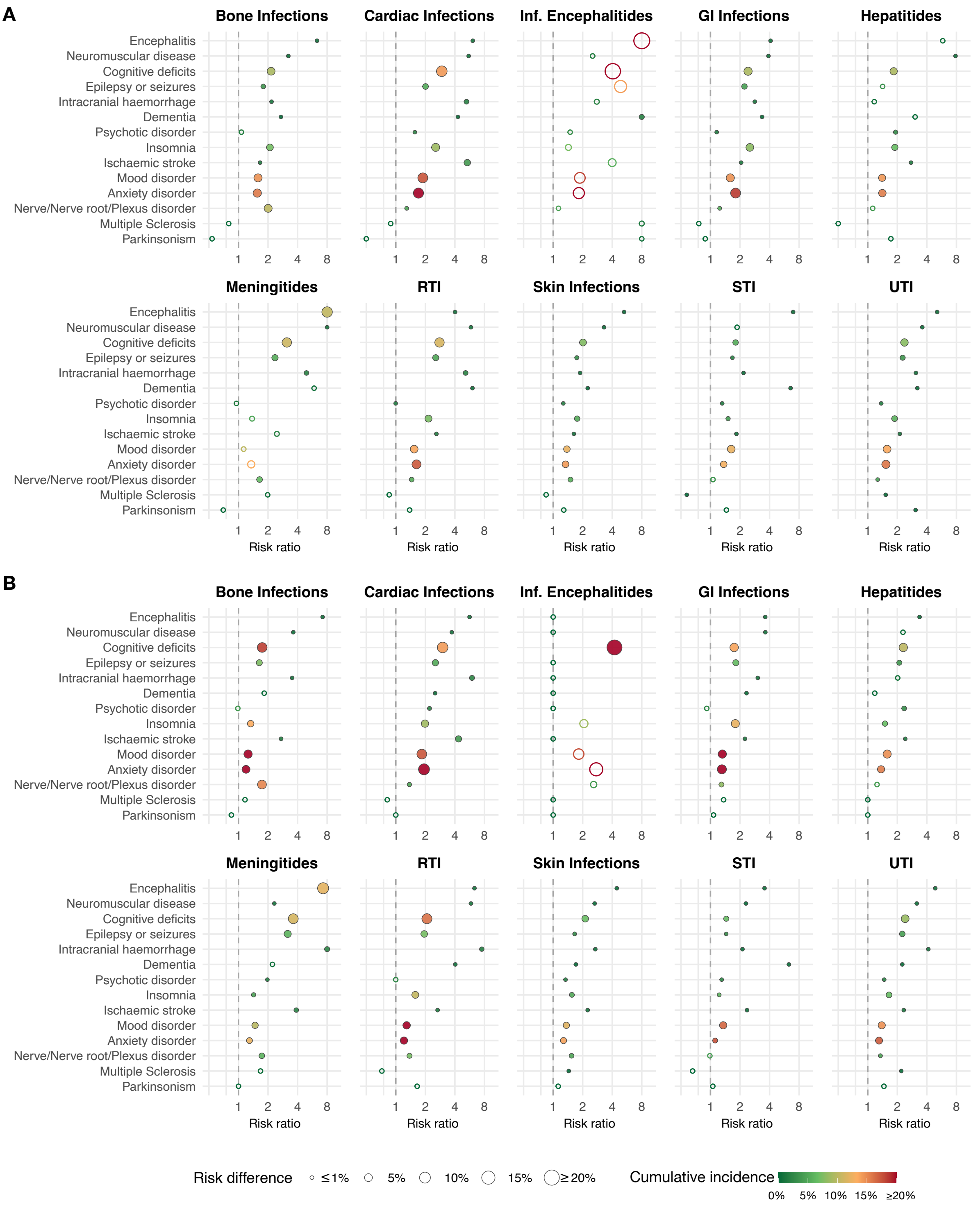

eFigure 3. Comparison of the risk of each outcome between hospitalised infections and (A) hospitalisations for other causes, (B) the general population in young adults**.** The location of each dot on the x-axis represents the risk ratio of the disorder occurring within 2 years; its size represents the absolute risk difference; and its colour represents the 2-year risk of the condition after the infection. Dots that are filled represent statistically significant risks after Bonferroni correction, whereas empty dots denote no statistical significance. Inf=Infectious; GI=Gastrointestinal. Other abbreviations as per eFigure 1.

© 2026 Taquet M et al. *JAMA Psychiatry*.

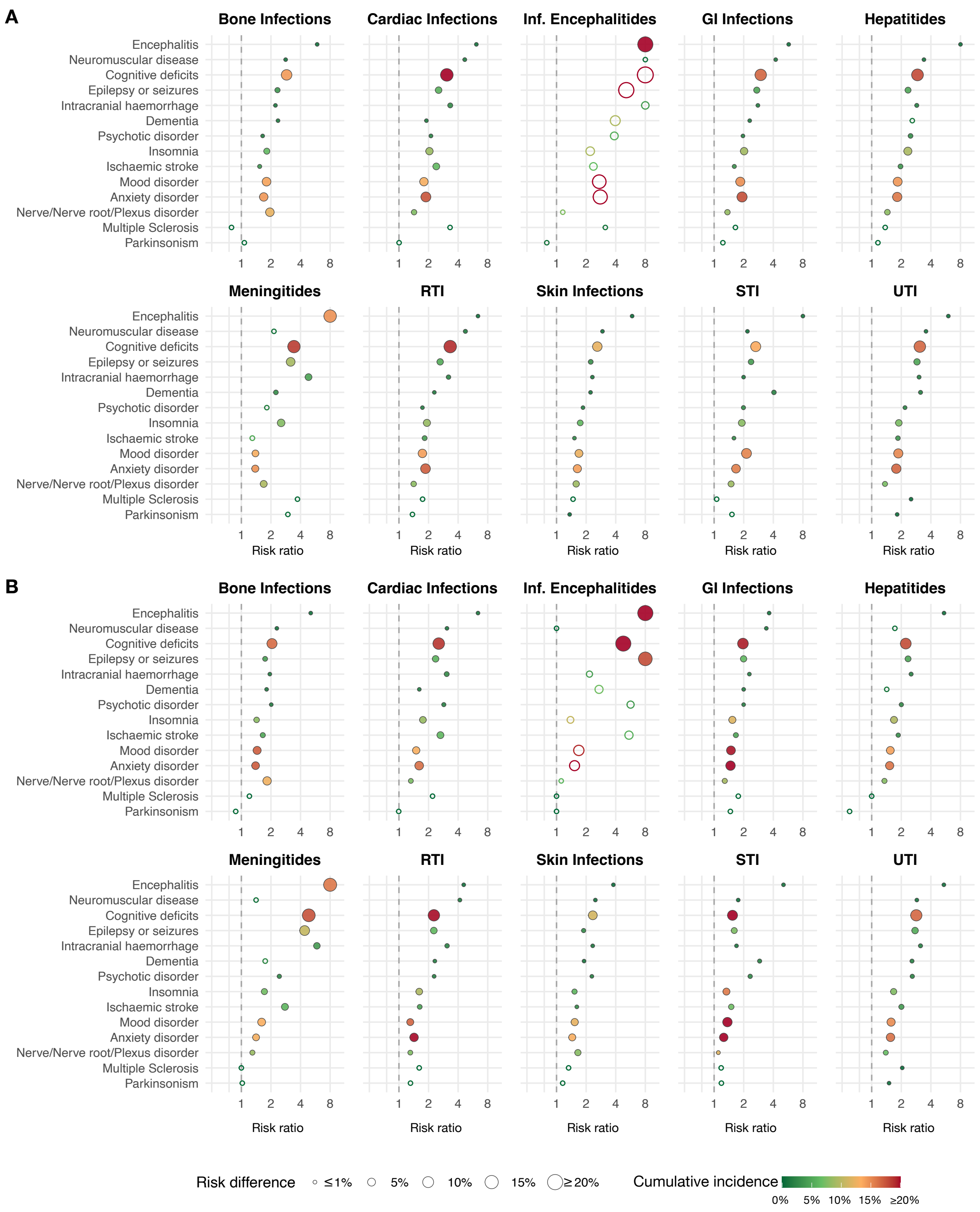

eFigure 4. Comparison of the risk of each outcome between hospitalised infections and (A) hospitalisations for other causes, (B) the general population in middle-aged adults**.** The location of each dot on the x-axis represents the risk ratio of the disorder occurring within 2 years; its size represents the absolute risk difference; and its colour represents the 2-year risk of the condition after the infection. Dots that are filled represent statistically significant risks after Bonferroni correction, whereas empty dots denote no statistical significance. Inf=Infectious; GI=Gastrointestinal. Other abbreviations as per eFigure 1.

© 2026 Taquet M et al. *JAMA Psychiatry*.

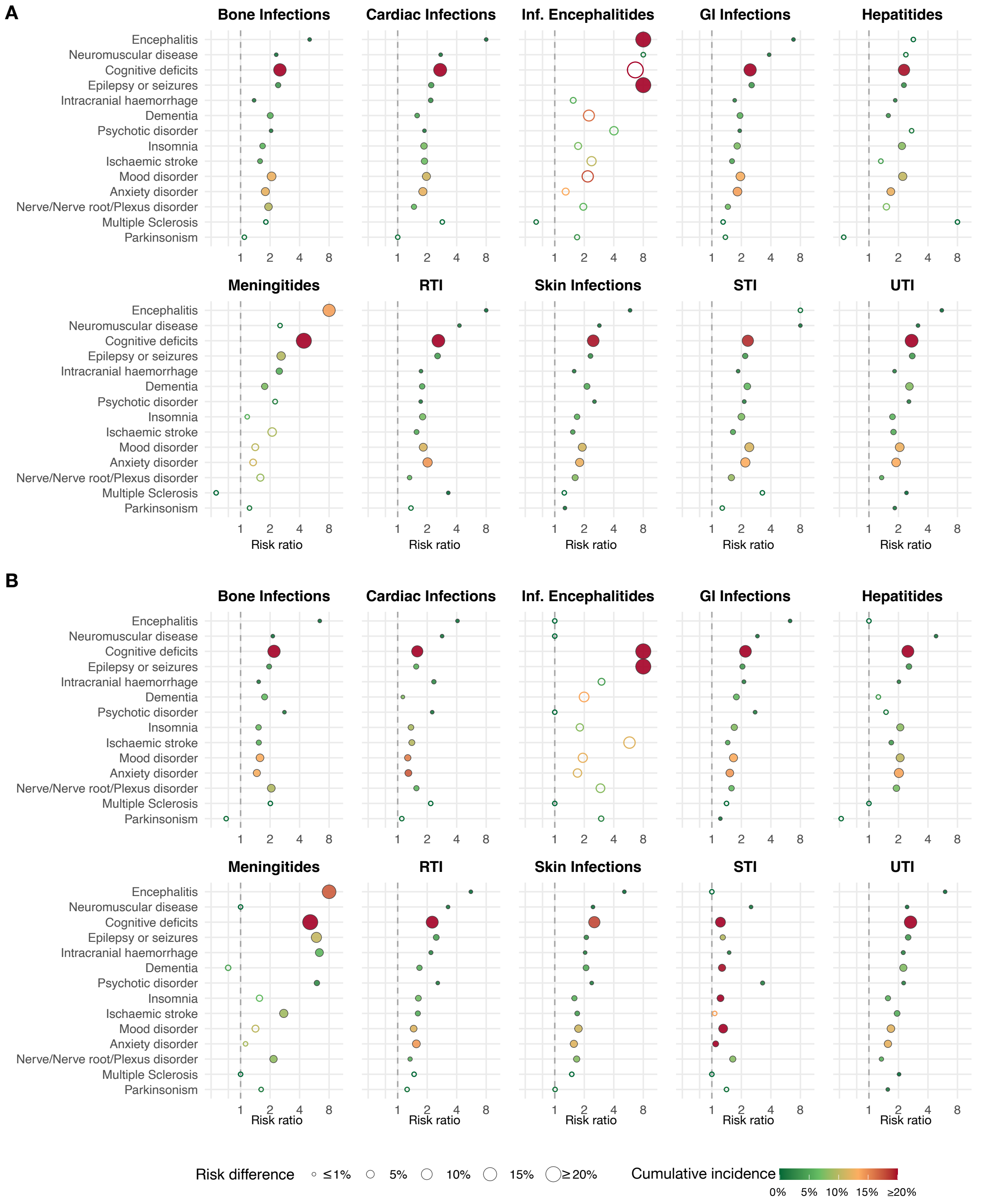

eFigure 5. Comparison of the risk of each outcome between hospitalised infections and (A) hospitalisations for other causes, (B) the general population in older adults**.** The location of each dot on the x-axis represents the risk ratio of the disorder occurring within 2 years; its size represents the absolute risk difference; and its colour represents the 2-year risk of the condition after the infection. Dots that are filled represent statistically significant risks after Bonferroni correction, whereas empty dots denote no statistical significance. Inf=Infectious; GI=Gastrointestinal. Other abbreviations as per eFigure 1.

© 2026 Taquet M et al. *JAMA Psychiatry*.

Supplementary tables

eTable 1. Comparison between each infection and hospitalisation for other causes (Pooled across Age Groups).

Infection	Outcome	RMTL Ratio	Cumulative Incidence (%)	Risk Difference (%)	P Value
**Bone Infections**	Anxiety disorder	1.86 (1.77, 1.96)	13.47	5.3	5.60E-123
**Bone Infections**	Cognitive deficits	2.97 (2.81, 3.14)	14.65	8.91	0.00E+00
**Bone Infections**	Dementia	2.48 (2.18, 2.81)	2.16	1.13	1.60E-45
**Bone Infections**	Encephalitis	4.81 (3.36, 6.89)	0.6	0.48	1.20E-17
**Bone Infections**	Epilepsy or seizures	2.48 (2.24, 2.74)	3.64	1.88	9.70E-69
**Bone Infections**	Insomnia	1.99 (1.85, 2.13)	6.19	2.76	4.70E-81
**Bone Infections**	Intracranial haemorrhage	2.38 (2.01, 2.81)	1.26	0.55	1.10E-23
**Bone Infections**	Ischaemic stroke	1.89 (1.71, 2.08)	3.36	1.2	3.00E-37
**Bone Infections**	Mood disorder	2.06 (1.95, 2.18)	12.8	5.54	3.50E-145
**Bone Infections**	Neuromuscular disease	2.96 (2.37, 3.70)	0.75	0.47	2.00E-21
**Bone Infections**	Nerve/Nerve root/Plexus disorder	2.15 (2.04, 2.27)	10.6	5.18	8.70E-174
**Bone Infections**	Parkinsonism	1.11 (0.86, 1.43)	0.3	0.01	4.40E-01
**Bone Infections**	Psychotic disorder	1.84 (1.60, 2.11)	1.57	0.53	2.40E-17
**Bone Infections**	Multiple Sclerosis	0.99 (0.65, 1.51)	0.1	0	9.60E-01
**Cardiac Infections**	Anxiety disorder	2.15 (1.99, 2.32)	16.04	7.07	1.90E-88
**Cardiac Infections**	Cognitive deficits	3.27 (3.02, 3.55)	19.08	12.37	3.20E-182
**Cardiac Infections**	Dementia	2.01 (1.68, 2.40)	2.16	0.87	8.20E-15
**Cardiac Infections**	Encephalitis	6.54 (3.78, 11.31)	0.74	0.65	2.00E-11
**Cardiac Infections**	Epilepsy or seizures	2.61 (2.28, 2.98)	5.1	2.82	1.40E-45
**Cardiac Infections**	Insomnia	2.17 (1.97, 2.39)	7.95	4.1	4.50E-55
**Cardiac Infections**	Intracranial haemorrhage	3.98 (3.21, 4.92)	2.68	1.75	4.20E-37
**Cardiac Infections**	Ischaemic stroke	2.72 (2.38, 3.11)	5.77	3.39	5.10E-49
**Cardiac Infections**	Mood disorder	2.23 (2.06, 2.43)	13.12	6.07	2.10E-80
**Cardiac Infections**	Neuromuscular disease	3.76 (2.76, 5.10)	1.13	0.83	2.70E-17
**Cardiac Infections**	Nerve/Nerve root/Plexus disorder	1.53 (1.40, 1.68)	6.27	1.82	3.10E-19
**Cardiac Infections**	Parkinsonism	0.85 (0.56, 1.28)	0.26	-0.03	4.40E-01
**Cardiac Infections**	Psychotic disorder	2.19 (1.79, 2.67)	1.88	0.87	2.80E-14
**Cardiac Infections**	Multiple Sclerosis	2.04 (1.13, 3.69)	0.17	0.07	1.90E-02
**Inf. Encephalitides**	Anxiety disorder	1.18 (0.93, 1.51)	28.38	4.72	1.80E-01

© 2026 Taquet M et al. *JAMA Psychiatry*.

**Inf. Encephalitides**	Cognitive deficits	1.81 (1.27, 2.59)	44.47	24.58	1.10E-03
**Inf. Encephalitides**	Dementia	2.32 (1.67, 3.24)	16.66	9.09	6.20E-07
**Inf. Encephalitides**	Encephalitis	4.75 (3.09, 7.29)	33.01	29.46	1.00E-12
**Inf. Encephalitides**	Epilepsy or seizures	2.44 (1.61, 3.71)	28.83	19.63	2.90E-05
**Inf. Encephalitides**	Insomnia	1.07 (0.79, 1.44)	15.8	-1.45	6.60E-01
**Inf. Encephalitides**	Intracranial haemorrhage	1.12 (0.70, 1.81)	6.52	0.28	6.30E-01
**Inf. Encephalitides**	Ischaemic stroke	1.57 (1.01, 2.45)	12.49	4.73	4.40E-02
**Inf. Encephalitides**	Mood disorder	1.74 (1.29, 2.34)	28.04	11.27	3.00E-04
**Inf. Encephalitides**	Neuromuscular disease	1.85 (0.91, 3.74)	2.96	1.23	8.90E-02
**Inf. Encephalitides**	Nerve/Nerve root/Plexus disorder	1.06 (0.76, 1.48)	12.52	2.48	7.30E-01
**Inf. Encephalitides**	Parkinsonism	1.09 (0.58, 2.04)	2.99	0.64	7.90E-01
**Inf. Encephalitides**	Psychotic disorder	2.86 (1.43, 5.73)	4.98	2.94	3.10E-03
**Inf. Encephalitides**	Multiple Sclerosis	1.29 (0.41, 4.02)	1.08	0.32	6.60E-01
**GI Infections**	Anxiety disorder	2.03 (1.94, 2.12)	14.12	6.17	8.80E-227
**GI Infections**	Cognitive deficits	2.84 (2.72, 2.97)	14.39	8.61	0.00E+00
**GI Infections**	Dementia	2.32 (2.10, 2.56)	2.13	1.04	1.50E-61
**GI Infections**	Encephalitis	4.00 (2.98, 5.37)	0.52	0.42	3.80E-20
**GI Infections**	Epilepsy or seizures	2.98 (2.75, 3.23)	4.37	2.54	3.20E-154
**GI Infections**	Insomnia	2.20 (2.08, 2.32)	7.01	3.49	1.30E-166
**GI Infections**	Intracranial haemorrhage	2.72 (2.38, 3.11)	1.44	0.72	2.00E-48
**GI Infections**	Ischaemic stroke	1.97 (1.81, 2.15)	2.8	1.05	6.80E-53
**GI Infections**	Mood disorder	2.02 (1.93, 2.12)	11.38	4.74	1.10E-185
**GI Infections**	Neuromuscular disease	3.84 (3.21, 4.58)	1.03	0.76	7.20E-50
**GI Infections**	Nerve/Nerve root/Plexus disorder	1.44 (1.37, 1.51)	6.04	1.59	5.80E-44
**GI Infections**	Parkinsonism	1.25 (1.02, 1.53)	0.39	0.09	3.00E-02
**GI Infections**	Psychotic disorder	2.03 (1.80, 2.30)	1.38	0.54	2.60E-29
**GI Infections**	Multiple Sclerosis	1.28 (0.89, 1.84)	0.12	0.02	1.90E-01
**Hepatitides**	Anxiety disorder	1.76 (1.59, 1.95)	16.82	5.94	1.40E-27
**Hepatitides**	Cognitive deficits	2.53 (2.26, 2.84)	16.27	9.15	2.30E-58
**Hepatitides**	Dementia	1.94 (1.42, 2.64)	1.53	0.74	2.70E-05
**Hepatitides**	Encephalitis	4.76 (2.14, 10.59)	0.49	0.4	1.30E-04
**Hepatitides**	Epilepsy or seizures	2.13 (1.76, 2.58)	4.34	1.93	1.20E-14
**Hepatitides**	Insomnia	2.32 (2.02, 2.66)	8.62	4.33	5.40E-33
**Hepatitides**	Intracranial haemorrhage	3.00 (2.11, 4.27)	1.57	0.77	1.00E-09
**Hepatitides**	Ischaemic stroke	2.06 (1.64, 2.59)	3.06	1.39	6.80E-10

© 2026 Taquet M et al. *JAMA Psychiatry*.

**Hepatitides**	Mood disorder	1.85 (1.65, 2.06)	15.01	5.46	3.10E-27
**Hepatitides**	Neuromuscular disease	3.88 (2.38, 6.31)	0.85	0.57	5.10E-08
**Hepatitides**	Nerve/Nerve root/Plexus disorder	1.33 (1.18, 1.52)	6.67	1.75	8.80E-06
**Hepatitides**	Parkinsonism	0.94 (0.53, 1.66)	0.23	-0.08	8.20E-01
**Hepatitides**	Psychotic disorder	2.72 (2.08, 3.56)	2.65	1.44	3.70E-13
**Hepatitides**	Multiple Sclerosis	0.70 (0.31, 1.59)	0.1	-0.03	4.00E-01
**Meningitides**	Anxiety disorder	1.17 (1.04, 1.33)	13.13	1.27	1.10E-02
**Meningitides**	Cognitive deficits	2.10 (1.80, 2.44)	15.59	8.41	8.00E-22
**Meningitides**	Dementia	2.02 (1.51, 2.70)	2.38	0.94	2.60E-06
**Meningitides**	Encephalitis	4.50 (3.44, 5.88)	11.24	10	3.00E-28
**Meningitides**	Epilepsy or seizures	2.36 (1.93, 2.90)	9.03	5.36	1.10E-16
**Meningitides**	Insomnia	1.47 (1.22, 1.77)	5.77	1.92	4.30E-05
**Meningitides**	Intracranial haemorrhage	4.73 (3.29, 6.81)	3.65	2.68	5.40E-17
**Meningitides**	Ischaemic stroke	1.50 (1.17, 1.92)	4.02	1.5	1.30E-03
**Meningitides**	Mood disorder	1.24 (1.08, 1.42)	10.1	0.48	1.80E-03
**Meningitides**	Neuromuscular disease	2.20 (1.35, 3.58)	0.73	0.24	1.60E-03
**Meningitides**	Nerve/Nerve root/Plexus disorder	1.46 (1.24, 1.73)	7.12	2.23	8.20E-06
**Meningitides**	Parkinsonism	0.85 (0.47, 1.54)	0.34	-0.01	5.90E-01
**Meningitides**	Psychotic disorder	1.09 (0.76, 1.58)	1.29	0	6.30E-01
**Meningitides**	Multiple Sclerosis	1.53 (0.69, 3.38)	0.31	0.07	2.90E-01
**RTI**	Anxiety disorder	1.78 (1.71, 1.85)	9.27	3.35	7.60E-170
**RTI**	Cognitive deficits	2.58 (2.47, 2.70)	11.13	6.44	0.00E+00
**RTI**	Dementia	2.07 (1.88, 2.28)	1.51	0.66	1.30E-50
**RTI**	Encephalitis	3.99 (3.04, 5.23)	0.45	0.37	1.10E-23
**RTI**	Epilepsy or seizures	2.46 (2.31, 2.63)	4.42	2.35	3.20E-165
**RTI**	Insomnia	1.89 (1.79, 1.99)	4.67	1.96	8.50E-118
**RTI**	Intracranial haemorrhage	2.81 (2.50, 3.16)	1.49	0.78	1.10E-66
**RTI**	Ischaemic stroke	1.88 (1.73, 2.04)	2.3	0.88	3.80E-52
**RTI**	Mood disorder	1.75 (1.67, 1.83)	6.85	2.28	1.20E-122
**RTI**	Neuromuscular disease	4.85 (4.11, 5.72)	0.95	0.73	2.00E-78
**RTI**	Nerve/Nerve root/Plexus disorder	1.39 (1.32, 1.46)	4.05	0.92	1.40E-35
**RTI**	Parkinsonism	1.33 (1.09, 1.62)	0.27	0.06	4.90E-03
**RTI**	Psychotic disorder	1.74 (1.54, 1.96)	0.88	0.22	7.30E-19
**RTI**	Multiple Sclerosis	1.58 (1.12, 2.23)	0.1	0.04	9.70E-03
**Skin Infections**	Anxiety disorder	1.70 (1.66, 1.74)	12.46	4.3	0.00E+00

© 2026 Taquet M et al. *JAMA Psychiatry*.

**Skin Infections**	Cognitive deficits	2.67 (2.60, 2.74)	11.76	6.72	0.00E+00
**Skin Infections**	Dementia	2.41 (2.25, 2.57)	1.74	0.91	1.20E-153
**Skin Infections**	Encephalitis	4.58 (3.71, 5.66)	0.37	0.3	2.10E-45
**Skin Infections**	Epilepsy or seizures	2.27 (2.16, 2.38)	3.04	1.48	6.40E-226
**Skin Infections**	Insomnia	1.85 (1.79, 1.91)	5.34	2.2	4.00E-268
**Skin Infections**	Intracranial haemorrhage	2.60 (2.38, 2.83)	1.12	0.51	2.90E-105
**Skin Infections**	Ischaemic stroke	1.88 (1.78, 1.99)	2.35	0.8	5.30E-119
**Skin Infections**	Mood disorder	1.83 (1.78, 1.88)	10.68	3.94	0.00E+00
**Skin Infections**	Neuromuscular disease	3.24 (2.88, 3.63)	0.66	0.43	3.00E-87
**Skin Infections**	Nerve/Nerve root/Plexus disorder	1.62 (1.57, 1.67)	6.88	2.51	2.10E-237
**Skin Infections**	Parkinsonism	1.34 (1.18, 1.53)	0.31	0.07	7.60E-06
**Skin Infections**	Psychotic disorder	2.15 (2.01, 2.30)	1.51	0.63	2.30E-105
**Skin Infections**	Multiple Sclerosis	1.16 (0.94, 1.43)	0.11	0.01	1.60E-01
**STI**	Anxiety disorder	1.62 (1.54, 1.70)	13.93	4.52	1.50E-76
**STI**	Cognitive deficits	2.28 (2.13, 2.44)	8.72	4.7	1.90E-128
**STI**	Dementia	3.93 (3.26, 4.75)	1.38	0.97	2.30E-46
**STI**	Encephalitis	6.31 (4.26, 9.34)	0.7	0.61	3.50E-20
**STI**	Epilepsy or seizures	2.04 (1.83, 2.28)	2.93	1.41	2.90E-36
**STI**	Insomnia	1.76 (1.63, 1.90)	5.46	2.22	2.60E-50
**STI**	Intracranial haemorrhage	2.52 (2.01, 3.15)	0.83	0.41	5.40E-16
**STI**	Ischaemic stroke	1.89 (1.65, 2.18)	1.69	0.64	4.40E-19
**STI**	Mood disorder	2.06 (1.94, 2.18)	13.44	5.93	1.70E-129
**STI**	Neuromuscular disease	2.62 (1.94, 3.54)	0.4	0.24	3.60E-10
**STI**	Nerve/Nerve root/Plexus disorder	1.35 (1.26, 1.44)	5.49	1.23	3.40E-18
**STI**	Parkinsonism	1.40 (0.95, 2.07)	0.16	0.04	8.90E-02
**STI**	Psychotic disorder	2.02 (1.77, 2.31)	1.93	0.71	1.10E-24
**STI**	Multiple Sclerosis	0.80 (0.52, 1.23)	0.11	-0.03	3.10E-01
**UTI**	Anxiety disorder	1.90 (1.85, 1.94)	14.27	5.95	0.00E+00
**UTI**	Cognitive deficits	2.98 (2.90, 3.06)	15.71	9.85	0.00E+00
**UTI**	Dementia	2.87 (2.72, 3.01)	3.49	2.13	0.00E+00
**UTI**	Encephalitis	4.67 (3.88, 5.61)	0.5	0.41	3.30E-60
**UTI**	Epilepsy or seizures	3.11 (2.96, 3.26)	4.32	2.68	0.00E+00
**UTI**	Insomnia	1.94 (1.87, 2.01)	6.03	2.68	0.00E+00
**UTI**	Intracranial haemorrhage	3.02 (2.78, 3.27)	1.6	0.87	4.50E-158
**UTI**	Ischaemic stroke	2.12 (2.02, 2.23)	3.38	1.48	2.90E-196

© 2026 Taquet M et al. *JAMA Psychiatry*.

**UTI**	Mood disorder	2.06 (2.00, 2.11)	12.63	5.67	0.00E+00
**UTI**	Neuromuscular disease	3.64 (3.26, 4.08)	0.81	0.57	4.20E-112
**UTI**	Nerve/Nerve root/Plexus disorder	1.36 (1.32, 1.41)	5.52	1.36	4.40E-84
**UTI**	Parkinsonism	1.87 (1.69, 2.08)	0.61	0.27	3.90E-32
**UTI**	Psychotic disorder	2.44 (2.27, 2.61)	1.75	0.88	1.40E-139
**UTI**	Multiple Sclerosis	2.22 (1.84, 2.68)	0.21	0.11	5.10E-17

Cumulative incidence is within the infection group.

© 2026 Taquet M et al. *JAMA Psychiatry*.

eTable 2. Comparison between each infection and the general population (Pooled across Age Groups)

Infection	Outcome	RMTL Ratio	Cumulative Incidence (%)	Risk Difference (%)	P Value
**Bone Infections**	Anxiety disorder	1.58 (1.52, 1.65)	16.75	4.45	6.20E-103
**Bone Infections**	Cognitive deficits	2.55 (2.44, 2.67)	17.90	9.12	0.00E+00
**Bone Infections**	Dementia	2.23 (2.01, 2.48)	2.35	1.08	2.50E-51
**Bone Infections**	Encephalitis	8.30 (6.18, 11.15)	0.60	0.50	4.90E-45
**Bone Infections**	Epilepsy or seizures	2.02 (1.86, 2.20)	4.44	1.93	1.40E-60
**Bone Infections**	Insomnia	1.66 (1.56, 1.76)	7.86	2.37	1.60E-62
**Bone Infections**	Intracranial haemorrhage	2.33 (2.03, 2.68)	1.20	0.55	1.70E-32
**Bone Infections**	Ischaemic stroke	1.94 (1.79, 2.11)	4.32	1.86	3.10E-55
**Bone Infections**	Mood disorder	1.64 (1.57, 1.72)	15.86	4.68	2.40E-106
**Bone Infections**	Multiple Sclerosis	1.53 (1.03, 2.27)	0.12	0.03	3.50E-02
**Bone Infections**	Neuromuscular disease	2.77 (2.28, 3.37)	0.69	0.39	2.20E-24
**Bone Infections**	Nerve/Nerve root/Plexus disorder	2.24 (2.13, 2.35)	11.10	5.28	1.90E-226
**Bone Infections**	Parkinsonism	0.95 (0.75, 1.19)	0.29	-0.09	6.50E-01
**Bone Infections**	Psychotic disorder	1.97 (1.75, 2.21)	1.66	0.68	2.50E-29
**Cardiac Infections**	Anxiety disorder	1.97 (1.86, 2.09)	17.71	6.18	1.70E-112
**Cardiac Infections**	Cognitive deficits	2.92 (2.75, 3.10)	19.47	10.46	9.20E-270
**Cardiac Infections**	Dementia	1.54 (1.34, 1.77)	2.41	0.57	7.70E-10
**Cardiac Infections**	Encephalitis	8.82 (6.33, 12.30)	0.89	0.73	9.20E-38
**Cardiac Infections**	Epilepsy or seizures	2.49 (2.25, 2.75)	5.90	3.06	8.20E-71
**Cardiac Infections**	Insomnia	2.05 (1.89, 2.21)	8.64	3.43	1.40E-71
**Cardiac Infections**	Intracranial haemorrhage	4.39 (3.74, 5.15)	2.61	1.78	1.30E-73
**Cardiac Infections**	Ischaemic stroke	3.15 (2.84, 3.49)	6.19	3.43	8.10E-104
**Cardiac Infections**	Mood disorder	1.90 (1.78, 2.02)	14.63	4.87	1.50E-84
**Cardiac Infections**	Multiple Sclerosis	1.84 (1.12, 3.00)	0.16	0.05	1.50E-02
**Cardiac Infections**	Neuromuscular disease	4.17 (3.25, 5.36)	1.01	0.68	5.40E-29
**Cardiac Infections**	Nerve/Nerve root/Plexus disorder	1.60 (1.48, 1.72)	6.14	1.82	2.60E-33
**Cardiac Infections**	Parkinsonism	1.31 (0.93, 1.84)	0.30	0.02	1.20E-01
**Cardiac Infections**	Psychotic disorder	3.06 (2.55, 3.68)	1.74	1.04	1.40E-32
**GI Infections**	Anxiety disorder	1.76 (1.69, 1.83)	16.10	5.28	6.20E-183
**GI Infections**	Cognitive deficits	2.63 (2.52, 2.75)	14.44	7.18	0.00E+00
**GI Infections**	Dementia	2.19 (1.98, 2.42)	1.78	0.79	6.30E-52

© 2026 Taquet M et al. *JAMA Psychiatry*.

**GI Infections**	Encephalitis	5.42 (4.42, 6.64)	0.60	0.45	1.70E-59
**GI Infections**	Epilepsy or seizures	2.42 (2.25, 2.59)	5.24	2.68	1.50E-132
**GI Infections**	Insomnia	1.98 (1.87, 2.09)	7.87	3.16	8.10E-137
**GI Infections**	Intracranial haemorrhage	2.99 (2.68, 3.34)	1.51	0.86	6.90E-85
**GI Infections**	Ischaemic stroke	1.94 (1.78, 2.11)	2.69	1.01	3.40E-51
**GI Infections**	Mood disorder	1.78 (1.70, 1.85)	12.45	4.11	1.30E-145
**GI Infections**	Multiple Sclerosis	1.46 (1.02, 2.10)	0.10	0.04	3.90E-02
**GI Infections**	Neuromuscular disease	4.02 (3.46, 4.67)	0.94	0.64	4.90E-73
**GI Infections**	Nerve/Nerve root/Plexus disorder	1.66 (1.58, 1.75)	5.97	1.81	1.00E-87
**GI Infections**	Parkinsonism	1.35 (1.12, 1.63)	0.36	0.06	1.60E-03
**GI Infections**	Psychotic disorder	2.28 (2.05, 2.53)	1.49	0.66	2.70E-54
**Hepatitides**	Anxiety disorder	1.71 (1.58, 1.86)	17.54	5.30	2.00E-37
**Hepatitides**	Cognitive deficits	2.80 (2.56, 3.07)	16.04	8.27	3.70E-111
**Hepatitides**	Dementia	1.27 (1.00, 1.63)	1.42	0.29	5.30E-02
**Hepatitides**	Encephalitis	5.34 (2.88, 9.91)	0.45	0.34	1.10E-07
**Hepatitides**	Epilepsy or seizures	2.61 (2.22, 3.06)	4.60	2.36	8.00E-32
**Hepatitides**	Insomnia	1.88 (1.68, 2.09)	8.68	3.16	2.00E-30
**Hepatitides**	Intracranial haemorrhage	2.35 (1.79, 3.07)	1.61	0.80	4.60E-10
**Hepatitides**	Ischaemic stroke	2.21 (1.82, 2.70)	2.73	1.13	3.70E-15
**Hepatitides**	Mood disorder	1.80 (1.64, 1.98)	15.21	4.97	3.60E-35
**Hepatitides**	Multiple Sclerosis	-	0.00	0.00	-
**Hepatitides**	Neuromuscular disease	2.69 (1.78, 4.06)	0.68	0.34	2.40E-06
**Hepatitides**	Nerve/Nerve root/Plexus disorder	1.50 (1.34, 1.68)	6.37	1.68	1.40E-12
**Hepatitides**	Parkinsonism	0.59 (0.33, 1.06)	0.19	-0.13	7.70E-02
**Hepatitides**	Psychotic disorder	2.37 (1.93, 2.90)	2.64	1.29	2.00E-16
**Inf. Encephalitides**	Anxiety disorder	1.19 (1.00, 1.43)	36.12	-0.50	5.60E-02
**Inf. Encephalitides**	Cognitive deficits	2.51 (1.98, 3.19)	46.04	23.30	3.00E-14
**Inf. Encephalitides**	Dementia	1.43 (1.07, 1.92)	18.74	5.59	1.60E-02
**Inf. Encephalitides**	Encephalitis	6.14 (4.33, 8.70)	20.97	17.21	1.90E-24
**Inf. Encephalitides**	Epilepsy or seizures	4.18 (2.78, 6.28)	15.04	9.82	5.50E-12
**Inf. Encephalitides**	Insomnia	1.24 (0.98, 1.56)	43.64	15.85	7.80E-02
**Inf. Encephalitides**	Intracranial haemorrhage	1.94 (1.19, 3.17)	7.76	3.55	8.10E-03
**Inf. Encephalitides**	Ischaemic stroke	1.78 (1.25, 2.54)	19.88	10.01	1.40E-03
**Inf. Encephalitides**	Mood disorder	1.27 (1.01, 1.60)	25.51	-0.05	4.00E-02
**Inf. Encephalitides**	Multiple Sclerosis	-	0.00	0.00	-

© 2026 Taquet M et al. *JAMA Psychiatry*.

**Inf. Encephalitides**	Neuromuscular disease	-	0.00	0.00	-
**Inf. Encephalitides**	Nerve/Nerve root/Plexus disorder	1.08 (0.82, 1.42)	11.90	-2.05	6.00E-01
**Inf. Encephalitides**	Parkinsonism	1.68 (0.66, 4.33)	6.65	2.71	2.80E-01
**Inf. Encephalitides**	Psychotic disorder	1.40 (0.63, 3.11)	1.43	0.27	4.10E-01
**Meningitides**	Anxiety disorder	1.33 (1.20, 1.49)	12.31	1.23	1.90E-07
**Meningitides**	Cognitive deficits	3.11 (2.75, 3.52)	15.73	8.28	3.60E-73
**Meningitides**	Dementia	1.06 (0.83, 1.34)	2.00	-0.26	6.50E-01
**Meningitides**	Encephalitis	11.02 (9.27, 13.09)	13.41	11.62	1.30E-163
**Meningitides**	Epilepsy or seizures	3.94 (3.34, 4.64)	9.52	6.18	2.50E-60
**Meningitides**	Insomnia	1.45 (1.24, 1.69)	5.67	0.69	2.20E-06
**Meningitides**	Intracranial haemorrhage	6.46 (4.91, 8.50)	3.89	2.91	1.40E-40
**Meningitides**	Ischaemic stroke	2.94 (2.40, 3.61)	4.30	2.18	3.50E-25
**Meningitides**	Mood disorder	1.51 (1.33, 1.70)	10.23	1.82	3.70E-11
**Meningitides**	Multiple Sclerosis	1.97 (0.88, 4.39)	0.19	0.03	9.80E-02
**Meningitides**	Neuromuscular disease	1.98 (1.28, 3.07)	0.67	0.19	2.20E-03
**Meningitides**	Nerve/Nerve root/Plexus disorder	1.80 (1.57, 2.06)	6.69	1.93	2.70E-17
**Meningitides**	Parkinsonism	1.18 (0.70, 1.98)	0.46	0.02	5.30E-01
**Meningitides**	Psychotic disorder	2.94 (2.17, 3.98)	1.71	0.74	2.60E-12
**RTI**	Anxiety disorder	1.60 (1.55, 1.66)	9.97	2.63	1.80E-157
**RTI**	Cognitive deficits	2.79 (2.69, 2.90)	10.64	5.46	0.00E+00
**RTI**	Dementia	2.13 (1.94, 2.34)	1.41	0.62	1.30E-57
**RTI**	Encephalitis	7.35 (6.04, 8.95)	0.53	0.42	4.10E-88
**RTI**	Epilepsy or seizures	2.43 (2.30, 2.57)	4.80	2.39	1.70E-231
**RTI**	Insomnia	1.89 (1.80, 1.98)	4.95	1.78	1.10E-142
**RTI**	Intracranial haemorrhage	3.84 (3.49, 4.22)	1.50	0.95	1.10E-170
**RTI**	Ischaemic stroke	2.14 (1.99, 2.31)	2.14	0.84	7.20E-88
**RTI**	Mood disorder	1.56 (1.50, 1.63)	7.43	1.87	3.80E-109
**RTI**	Multiple Sclerosis	1.23 (0.89, 1.70)	0.08	0.01	2.10E-01
**RTI**	Neuromuscular disease	4.83 (4.21, 5.55)	0.91	0.67	7.50E-111
**RTI**	Nerve/Nerve root/Plexus disorder	1.49 (1.42, 1.56)	3.89	0.92	1.00E-62
**RTI**	Parkinsonism	1.32 (1.13, 1.56)	0.32	0.05	6.60E-04
**RTI**	Psychotic disorder	2.26 (2.05, 2.50)	1.11	0.49	3.40E-59
**Skin Infections**	Anxiety disorder	1.61 (1.57, 1.64)	11.67	3.40	0.00E+00
**Skin Infections**	Cognitive deficits	2.88 (2.80, 2.96)	10.22	5.78	0.00E+00
**Skin Infections**	Dementia	2.33 (2.20, 2.48)	1.50	0.76	4.70E-166

© 2026 Taquet M et al. *JAMA Psychiatry*.

**Skin Infections**	Encephalitis	6.28 (5.42, 7.28)	0.42	0.32	1.50E-131
**Skin Infections**	Epilepsy or seizures	2.03 (1.94, 2.13)	2.82	1.27	6.20E-192
**Skin Infections**	Insomnia	1.78 (1.72, 1.84)	5.07	1.80	7.40E-252
**Skin Infections**	Intracranial haemorrhage	2.87 (2.68, 3.07)	1.12	0.63	7.50E-196
**Skin Infections**	Ischaemic stroke	1.98 (1.88, 2.08)	2.08	0.88	5.90E-147
**Skin Infections**	Mood disorder	1.73 (1.68, 1.77)	10.04	3.45	0.00E+00
**Skin Infections**	Multiple Sclerosis	1.45 (1.21, 1.74)	0.12	0.04	4.70E-05
**Skin Infections**	Neuromuscular disease	3.07 (2.77, 3.41)	0.58	0.35	1.60E-98
**Skin Infections**	Nerve/Nerve root/Plexus disorder	1.89 (1.84, 1.95)	6.18	2.42	0.00E+00
**Skin Infections**	Parkinsonism	1.14 (1.03, 1.27)	0.31	0.01	1.30E-02
**Skin Infections**	Psychotic disorder	2.20 (2.07, 2.33)	1.56	0.76	1.60E-144
**STI**	Anxiety disorder	1.30 (1.25, 1.36)	20.93	2.76	3.10E-33
**STI**	Cognitive deficits	1.74 (1.65, 1.84)	11.78	3.42	1.00E-83
**STI**	Dementia	2.57 (2.23, 2.96)	1.36	0.76	3.50E-38
**STI**	Encephalitis	5.83 (4.46, 7.61)	0.62	0.47	3.20E-38
**STI**	Epilepsy or seizures	1.76 (1.60, 1.93)	4.25	1.34	7.10E-31
**STI**	Insomnia	1.42 (1.33, 1.51)	8.53	1.59	1.20E-26
**STI**	Intracranial haemorrhage	2.30 (1.91, 2.77)	0.70	0.30	2.60E-18
**STI**	Ischaemic stroke	1.72 (1.53, 1.93)	1.96	0.66	3.70E-19
**STI**	Mood disorder	1.53 (1.46, 1.60)	19.64	4.93	2.90E-68
**STI**	Multiple Sclerosis	0.71 (0.46, 1.08)	0.09	-0.02	1.10E-01
**STI**	Neuromuscular disease	2.48 (1.91, 3.23)	0.37	0.19	1.40E-11
**STI**	Nerve/Nerve root/Plexus disorder	1.26 (1.18, 1.35)	6.15	0.45	2.50E-11
**STI**	Parkinsonism	1.16 (0.81, 1.65)	0.15	0.02	4.20E-01
**STI**	Psychotic disorder	1.89 (1.68, 2.12)	2.33	0.94	8.30E-27
**UTI**	Anxiety disorder	1.77 (1.73, 1.81)	13.65	4.55	0.00E+00
**UTI**	Cognitive deficits	3.44 (3.35, 3.53)	15.31	9.41	0.00E+00
**UTI**	Dementia	2.73 (2.61, 2.85)	3.20	1.79	0.00E+00
**UTI**	Encephalitis	7.70 (6.73, 8.81)	0.61	0.49	3.30E-194
**UTI**	Epilepsy or seizures	3.04 (2.92, 3.18)	4.55	2.73	0.00E+00
**UTI**	Insomnia	1.90 (1.84, 1.97)	6.16	2.34	0.00E+00
**UTI**	Intracranial haemorrhage	3.73 (3.51, 3.96)	1.72	1.07	0.00E+00
**UTI**	Ischaemic stroke	2.44 (2.34, 2.55)	3.44	1.69	0.00E+00
**UTI**	Mood disorder	1.85 (1.81, 1.90)	12.31	4.51	0.00E+00
**UTI**	Multiple Sclerosis	2.30 (1.96, 2.69)	0.21	0.11	4.90E-25

© 2026 Taquet M et al. *JAMA Psychiatry*.

**UTI**	Neuromuscular disease	3.62 (3.28, 3.99)	0.74	0.48	4.50E-146
**UTI**	Nerve/Nerve root/Plexus disorder	1.55 (1.51, 1.59)	5.31	1.40	3.20E-196
**UTI**	Parkinsonism	1.73 (1.59, 1.88)	0.62	0.20	2.50E-38
**UTI**	Psychotic disorder	2.70 (2.55, 2.86)	1.92	1.04	4.30E-252

Cumulative incidence is within the infection group.

© 2026 Taquet M et al. *JAMA Psychiatry*.

eTable 3. Moderation of RMTL ratios by age group and infection (hospitalisation for other causes).

Infection	Age group	Ratio (95% CI)	P Value
**Average**	Children	0.79 (0.74, 0.84)	1e-19
**Average**	Young adults	1.02 (0.99, 1.05)	0.11
**Average**	Middle-aged adults	1.12 (1.09, 1.15)	2.1e-21
**Average**	Older adults	1.11 (1.08, 1.14)	3.4e-19
**Bone Infections**	Children	0.68 (0.52, 0.89)	0.0013
**Bone Infections**	Young adults	1.13 (0.99, 1.30)	0.023
**Bone Infections**	Middle-aged adults	1.15 (1.03, 1.29)	0.0045
**Bone Infections**	Older adults	1.13 (1.00, 1.27)	0.016
**Cardiac Infections**	Children	0.83 (0.56, 1.21)	0.3
**Cardiac Infections**	Young adults	1.13 (0.95, 1.35)	0.3
**Cardiac Infections**	Middle-aged adults	1.08 (0.92, 1.27)	0.3
**Cardiac Infections**	Older adults	0.99 (0.84, 1.16)	0.86
**GI Infections**	Children	0.89 (0.73, 1.08)	0.26
**GI Infections**	Young adults	1.05 (0.94, 1.18)	0.34
**GI Infections**	Middle-aged adults	1.07 (0.98, 1.18)	0.21
**GI Infections**	Older adults	0.99 (0.90, 1.09)	0.88
**Hepatitides**	Children	0.81 (0.57, 1.16)	0.29
**Hepatitides**	Young adults	0.99 (0.81, 1.20)	0.87
**Hepatitides**	Middle-aged adults	1.19 (1.00, 1.42)	0.051
**Hepatitides**	Older adults	1.04 (0.85, 1.28)	0.79
**Inf. Encephalitides**	Children	1.42 (0.82, 2.47)	0.46
**Inf. Encephalitides**	Young adults	0.88 (0.56, 1.38)	0.63
**Inf. Encephalitides**	Middle-aged adults	0.96 (0.66, 1.41)	0.8
**Inf. Encephalitides**	Older adults	0.83 (0.56, 1.24)	0.5
**Meningitides**	Children	0.96 (0.72, 1.29)	0.75
**Meningitides**	Young adults	1.05 (0.86, 1.29)	0.71
**Meningitides**	Middle-aged adults	1.09 (0.89, 1.32)	0.57
**Meningitides**	Older adults	0.91 (0.73, 1.13)	0.57
**RTI**	Children	0.72 (0.60, 0.86)	1.6e-05
**RTI**	Young adults	1.14 (1.03, 1.27)	0.0027
**RTI**	Middle-aged adults	1.17 (1.07, 1.27)	3.5e-05

© 2026 Taquet M et al. *JAMA Psychiatry.*

**RTI**	Older adults	1.04 (0.95, 1.14)	0.26
**Skin Infections**	Children	0.79 (0.66, 0.94)	0.00094
**Skin Infections**	Young adults	1.01 (0.92, 1.10)	0.83
**Skin Infections**	Middle-aged adults	1.12 (1.04, 1.21)	0.00053
**Skin Infections**	Older adults	1.13 (1.04, 1.22)	0.00053
**STI**	Children	0.76 (0.58, 1.00)	0.019
**STI**	Young adults	0.94 (0.83, 1.07)	0.26
**STI**	Middle-aged adults	1.16 (1.02, 1.31)	0.0096
**STI**	Older adults	1.20 (1.02, 1.40)	0.0096
**UTI**	Children	0.86 (0.72, 1.04)	0.062
**UTI**	Young adults	0.99 (0.90, 1.08)	0.71
**UTI**	Middle-aged adults	1.10 (1.01, 1.19)	0.014
**UTI**	Older adults	1.07 (0.99, 1.16)	0.062

© 2026 Taquet M et al. *JAMA Psychiatry*.

eTable 4. Moderation of RMTL ratio by age group and outcome (hospitalisation for other causes).

Outcome	Age group	Ratio (95% CI)	P Value
**Average**	Children	0.79 (0.74, 0.84)	1e-19
**Average**	Young adults	1.02 (0.99, 1.05)	0.11
**Average**	Middle-aged adults	1.12 (1.09, 1.15)	2.1e-21
**Average**	Older adults	1.11 (1.08, 1.14)	3.4e-19
**Cognitive deficits**	Children	0.75 (0.66, 0.84)	3.3e-09
**Cognitive deficits**	Young adults	0.99 (0.91, 1.08)	0.81
**Cognitive deficits**	Middle-aged adults	1.24 (1.16, 1.33)	6.5e-13
**Cognitive deficits**	Older adults	1.09 (1.01, 1.17)	0.0036
**Encephalitis**	Children	0.73 (0.49, 1.09)	0.1
**Encephalitis**	Young adults	0.93 (0.68, 1.28)	0.61
**Encephalitis**	Middle-aged adults	1.36 (0.99, 1.88)	0.061
**Encephalitis**	Older adults	1.07 (0.75, 1.53)	0.61
**Neuromuscular disease**	Children	1.06 (0.74, 1.53)	0.94
**Neuromuscular disease**	Young adults	1.03 (0.78, 1.37)	0.94
**Neuromuscular disease**	Middle-aged adults	0.99 (0.80, 1.23)	0.94
**Neuromuscular disease**	Older adults	0.91 (0.74, 1.14)	0.94
**Intracranial haemorrhage**	Children	0.77 (0.58, 1.01)	0.022
**Intracranial haemorrhage**	Young adults	1.47 (1.16, 1.87)	0.00017
**Intracranial haemorrhage**	Middle-aged adults	1.14 (0.96, 1.36)	0.052
**Intracranial haemorrhage**	Older adults	0.78 (0.66, 0.91)	0.00017
**Epilepsy or seizures**	Children	0.80 (0.70, 0.92)	9e-05
**Epilepsy or seizures**	Young adults	0.92 (0.83, 1.03)	0.064
**Epilepsy or seizures**	Middle-aged adults	1.17 (1.06, 1.29)	9e-05
**Epilepsy or seizures**	Older adults	1.15 (1.04, 1.28)	0.00086
**Dementia**	Children	0.85 (0.36, 1.96)	0.82
**Dementia**	Young adults	1.40 (0.92, 2.14)	0.18
**Dementia**	Middle-aged adults	1.01 (0.73, 1.39)	0.96
**Dementia**	Older adults	0.84 (0.62, 1.14)	0.29
**Psychotic disorder**	Children	0.53 (0.36, 0.77)	3e-05
**Psychotic disorder**	Young adults	0.94 (0.79, 1.12)	0.4
**Psychotic disorder**	Middle-aged adults	1.34 (1.13, 1.58)	3e-05

© 2026 Taquet M et al. *JAMA Psychiatry*.

**Psychotic disorder**	Older adults	1.50 (1.25, 1.79)	9.9e-08
**Ischaemic stroke**	Children	1.07 (0.73, 1.56)	0.67
**Ischaemic stroke**	Young adults	1.22 (1.00, 1.50)	0.027
**Ischaemic stroke**	Middle-aged adults	0.90 (0.77, 1.06)	0.15
**Ischaemic stroke**	Older adults	0.85 (0.73, 0.99)	0.027
**Mood disorder**	Children	0.72 (0.63, 0.82)	1.8e-09
**Mood disorder**	Young adults	1.03 (0.95, 1.11)	0.36
**Mood disorder**	Middle-aged adults	1.13 (1.06, 1.22)	1.6e-05
**Mood disorder**	Older adults	1.19 (1.10, 1.28)	4.6e-08
**Insomnia**	Children	0.86 (0.74, 1.00)	0.023
**Insomnia**	Young adults	1.13 (1.03, 1.24)	0.0032
**Insomnia**	Middle-aged adults	1.07 (0.99, 1.17)	0.038
**Insomnia**	Older adults	0.96 (0.88, 1.05)	0.22
**Anxiety disorder**	Children	0.75 (0.67, 0.83)	7.6e-11
**Anxiety disorder**	Young adults	0.98 (0.91, 1.06)	0.52
**Anxiety disorder**	Middle-aged adults	1.15 (1.08, 1.23)	1.9e-07
**Anxiety disorder**	Older adults	1.19 (1.11, 1.27)	3.7e-09
**Nerve/Nerve root/Plexus disorder**	Children	1.32 (1.06, 1.63)	0.0029
**Nerve/Nerve root/Plexus disorder**	Young adults	0.90 (0.81, 1.00)	0.019
**Nerve/Nerve root/Plexus disorder**	Middle-aged adults	0.95 (0.87, 1.04)	0.18
**Nerve/Nerve root/Plexus disorder**	Older adults	0.88 (0.80, 0.97)	0.0029
**Multiple Sclerosis**	Children	0.86 (0.27, 2.75)	0.75
**Multiple Sclerosis**	Young adults	0.78 (0.47, 1.30)	0.47
**Multiple Sclerosis**	Middle-aged adults	1.14 (0.70, 1.86)	0.66
**Multiple Sclerosis**	Older adults	1.29 (0.75, 2.22)	0.47
**Parkinsonism**	Children	1.24 (0.26, 5.82)	0.98
**Parkinsonism**	Young adults	0.87 (0.42, 1.80)	0.98
**Parkinsonism**	Middle-aged adults	1.00 (0.56, 1.78)	1
**Parkinsonism**	Older adults	0.93 (0.53, 1.62)	0.98

© 2026 Taquet M et al. *JAMA Psychiatry*.

eTable 5. Moderation of absolute risks by age group and infection (hospitalisation for other causes).

Infection	Age group	Difference (95% CI)	P Value
**Average**	Children	-0.33 (-0.38, -0.27)	1.1e-44
**Average**	Young adults	-0.16 (-0.22, -0.091)	5.1e-09
**Average**	Middle-aged adults	0.059 (-0.0075, 0.13)	0.026
**Average**	Older adults	0.42 (0.33, 0.51)	2e-28
**Bone Infections**	Children	-1.51 (-1.83, -1.19)	3.5e-28
**Bone Infections**	Young adults	-0.28 (-0.50, -0.059)	0.0016
**Bone Infections**	Middle-aged adults	0.69 (0.48, 0.90)	7.8e-16
**Bone Infections**	Older adults	1.10 (0.87, 1.33)	3.5e-28
**Cardiac Infections**	Children	-1.42 (-2.02, -0.83)	8.5e-09
**Cardiac Infections**	Young adults	-0.31 (-0.63, 0.0064)	0.014
**Cardiac Infections**	Middle-aged adults	0.70 (0.38, 1.02)	1.1e-07
**Cardiac Infections**	Older adults	1.04 (0.70, 1.38)	3.7e-13
**GI Infections**	Children	-1.54 (-1.68, -1.40)	2.1e-99
**GI Infections**	Young adults	-0.28 (-0.47, -0.10)	0.00011
**GI Infections**	Middle-aged adults	0.76 (0.58, 0.94)	1e-22
**GI Infections**	Older adults	1.06 (0.89, 1.24)	5.4e-41
**Hepatitides**	Children	-1.65 (-2.72, -0.58)	0.00018
**Hepatitides**	Young adults	-0.31 (-0.78, 0.15)	0.093
**Hepatitides**	Middle-aged adults	0.82 (0.32, 1.31)	9e-05
**Hepatitides**	Older adults	1.14 (0.69, 1.60)	3.7e-09
**Inf. Encephalitides**	Children	-1.98 (-8.61, 4.66)	0.95
**Inf. Encephalitides**	Young adults	0.21 (-7.34, 7.75)	0.95
**Inf. Encephalitides**	Middle-aged adults	0.34 (-5.27, 5.96)	0.95
**Inf. Encephalitides**	Older adults	1.43 (-5.02, 7.88)	0.95
**Meningitides**	Children	-1.84 (-2.37, -1.32)	9.2e-17
**Meningitides**	Young adults	-0.28 (-0.99, 0.42)	0.31
**Meningitides**	Middle-aged adults	1.04 (0.19, 1.89)	0.0048
**Meningitides**	Older adults	1.09 (-0.15, 2.33)	0.037
**RTI**	Children	-1.62 (-1.75, -1.50)	4.3e-123
**RTI**	Young adults	-0.22 (-0.41, -0.028)	0.0042
**RTI**	Middle-aged adults	0.77 (0.59, 0.96)	3.2e-23

© 2026 Taquet M et al. *JAMA Psychiatry*.

**RTI**	Older adults	1.07 (0.89, 1.25)	1.3e-40
**Skin Infections**	Children	-1.49 (-1.60, -1.38)	4.5e-129
**Skin Infections**	Young adults	-0.29 (-0.43, -0.16)	3.6e-08
**Skin Infections**	Middle-aged adults	0.68 (0.56, 0.81)	9.6e-35
**Skin Infections**	Older adults	1.10 (0.96, 1.24)	1.2e-62
**STI**	Children	-1.72 (-2.09, -1.34)	1.2e-26
**STI**	Young adults	-0.33 (-0.55, -0.12)	0.00013
**STI**	Middle-aged adults	0.77 (0.53, 1.02)	5e-14
**STI**	Older adults	1.27 (0.89, 1.66)	4.5e-15
**UTI**	Children	-1.57 (-1.70, -1.45)	3e-119
**UTI**	Young adults	-0.31 (-0.45, -0.18)	2.3e-08
**UTI**	Middle-aged adults	0.80 (0.66, 0.94)	1.9e-38
**UTI**	Older adults	1.09 (0.95, 1.23)	2.5e-62

The ‘difference’ represents the moderation of the absolute risk difference by the age group, in %. For instance, in children, the absolute risk difference of outcomes after bone infections (compared to non-infectious hospitalisations) were on average 1.51% lower than in the average across age groups.

© 2026 Taquet M et al. *JAMA Psychiatry*.

eTable 6. Moderation of absolute risks by age group and outcome (hospitalisation for other causes).

Outcome	Age group	Difference (95% CI)	P Value
**Average**	Children	-0.33 (-0.38, -0.27)	1.1e-44
**Average**	Young adults	-0.16 (-0.22, -0.091)	5.1e-09
**Average**	Middle-aged adults	0.059 (-0.0075, 0.13)	0.026
**Average**	Older adults	0.42 (0.33, 0.51)	2e-28
**Cognitive deficits**	Children	-5.95 (-6.86, -5.05)	1.3e-47
**Cognitive deficits**	Young adults	-2.80 (-3.78, -1.82)	3.9e-12
**Cognitive deficits**	Middle-aged adults	2.47 (1.58, 3.35)	9.9e-12
**Cognitive deficits**	Older adults	6.29 (5.22, 7.36)	4.7e-40
**Encephalitis**	Children	-0.15 (-0.84, 0.54)	0.88
**Encephalitis**	Young adults	0.12 (-0.65, 0.89)	0.88
**Encephalitis**	Middle-aged adults	0.063 (-0.52, 0.64)	0.88
**Encephalitis**	Older adults	-0.04 (-0.71, 0.63)	0.88
**Neuromuscular disease**	Children	-0.26 (-0.95, 0.44)	0.77
**Neuromuscular disease**	Young adults	-0.091 (-0.86, 0.68)	0.77
**Neuromuscular disease**	Middle-aged adults	0.13 (-0.46, 0.72)	0.77
**Neuromuscular disease**	Older adults	0.22 (-0.46, 0.90)	0.77
**Intracranial haemorrhage**	Children	-0.51 (-1.22, 0.20)	0.3
**Intracranial haemorrhage**	Young adults	-0.081 (-0.86, 0.70)	0.8
**Intracranial haemorrhage**	Middle-aged adults	0.32 (-0.29, 0.92)	0.38
**Intracranial haemorrhage**	Older adults	0.27 (-0.43, 0.97)	0.45
**Epilepsy or seizures**	Children	-0.76 (-1.61, 0.096)	0.083
**Epilepsy or seizures**	Young adults	-0.24 (-1.10, 0.61)	0.48
**Epilepsy or seizures**	Middle-aged adults	0.55 (-0.13, 1.23)	0.083
**Epilepsy or seizures**	Older adults	0.45 (-0.31, 1.20)	0.19
**Dementia**	Children	-1.16 (-1.85, -0.46)	7.3e-05
**Dementia**	Young adults	-0.82 (-1.60, -0.044)	0.011
**Dementia**	Middle-aged adults	-0.30 (-0.91, 0.30)	0.21
**Dementia**	Older adults	2.28 (1.51, 3.05)	2.3e-12
**Psychotic disorder**	Children	-0.68 (-1.37, 0.018)	0.06
**Psychotic disorder**	Young adults	-0.12 (-0.92, 0.68)	0.71
**Psychotic disorder**	Middle-aged adults	0.35 (-0.26, 0.97)	0.2

© 2026 Taquet M et al. *JAMA Psychiatry*.

**Psychotic disorder**	Older adults	0.44 (-0.26, 1.14)	0.2
**Ischaemic stroke**	Children	-0.91 (-1.62, -0.21)	0.0026
**Ischaemic stroke**	Young adults	-0.42 (-1.21, 0.38)	0.25
**Ischaemic stroke**	Middle-aged adults	0.27 (-0.37, 0.92)	0.29
**Ischaemic stroke**	Older adults	1.06 (0.30, 1.83)	0.0022
**Mood disorder**	Children	-4.45 (-5.31, -3.59)	2.8e-32
**Mood disorder**	Young adults	0.48 (-0.67, 1.64)	0.29
**Mood disorder**	Middle-aged adults	2.04 (1.08, 3.00)	3.3e-07
**Mood disorder**	Older adults	1.93 (0.94, 2.92)	2e-06
**Insomnia**	Children	-1.95 (-2.73, -1.17)	3.7e-09
**Insomnia**	Young adults	0.42 (-0.49, 1.33)	0.25
**Insomnia**	Middle-aged adults	1.06 (0.31, 1.81)	0.00089
**Insomnia**	Older adults	0.48 (-0.35, 1.30)	0.2
**Anxiety disorder**	Children	-4.45 (-5.40, -3.49)	3.7e-27
**Anxiety disorder**	Young adults	0.51 (-0.69, 1.71)	0.29
**Anxiety disorder**	Middle-aged adults	2.17 (1.18, 3.16)	1.3e-07
**Anxiety disorder**	Older adults	1.77 (0.75, 2.79)	2.1e-05
**Nerve/Nerve root/Plexus disorder**	Children	-1.37 (-2.13, -0.62)	2.6e-05
**Nerve/Nerve root/Plexus disorder**	Young adults	-0.33 (-1.23, 0.57)	0.36
**Nerve/Nerve root/Plexus disorder**	Middle-aged adults	1.18 (0.41, 1.94)	0.00025
**Nerve/Nerve root/Plexus disorder**	Older adults	0.52 (-0.30, 1.35)	0.15
**Multiple Sclerosis**	Children	-0.12 (-0.80, 0.56)	0.99
**Multiple Sclerosis**	Young adults	0.0031 (-0.76, 0.77)	0.99
**Multiple Sclerosis**	Middle-aged adults	0.052 (-0.52, 0.63)	0.99
**Multiple Sclerosis**	Older adults	0.061 (-0.60, 0.72)	0.99
**Parkinsonism**	Children	-0.18 (-0.86, 0.50)	0.96
**Parkinsonism**	Young adults	-0.016 (-0.78, 0.75)	0.96
**Parkinsonism**	Middle-aged adults	-0.023 (-0.60, 0.56)	0.96
**Parkinsonism**	Older adults	0.22 (-0.46, 0.90)	0.96

The ‘difference’ represents the moderation of the absolute risk difference by the age group, in %. For instance, in children, the absolute risk difference of cognitive deficits (averaged over all infections) was 5.95% lower than in the average across age groups and infections.

© 2026 Taquet M et al. *JAMA Psychiatry.*

eTable 7. Moderation of RMTL ratios by age group and infection (general population).

Infection	Age group	Difference (95% CI)	P Value
**Average**	Children	1.21 (1.16, 1.26)	8.90E-27
**Average**	Young adults	0.97 (0.95, 0.99)	7.00E-04
**Average**	Middle-aged adults	0.90 (0.88, 0.92)	2.20E-33
**Average**	Older adults	0.95 (0.93, 0.96)	1.30E-11
**Bone Infections**	Children	1.21 (0.85, 1.72)	0.36
**Bone Infections**	Young adults	0.94 (0.80, 1.10)	0.42
**Bone Infections**	Middle-aged adults	0.92 (0.80, 1.05)	0.36
**Bone Infections**	Older adults	0.96 (0.83, 1.10)	0.45
**Cardiac Infections**	Children	0.85 (0.60, 1.21)	0.33
**Cardiac Infections**	Young adults	1.27 (1.08, 1.49)	0.0011
**Cardiac Infections**	Middle-aged adults	1.02 (0.88, 1.18)	0.75
**Cardiac Infections**	Older adults	0.91 (0.79, 1.05)	0.22
**GI Infections**	Children	0.98 (0.57, 1.67)	0.92
**GI Infections**	Young adults	1.03 (0.71, 1.48)	0.92
**GI Infections**	Middle-aged adults	0.97 (0.72, 1.30)	0.92
**GI Infections**	Older adults	1.03 (0.77, 1.38)	0.92
**Hepatitides**	Children	1.22 (0.90, 1.67)	0.14
**Hepatitides**	Young adults	0.91 (0.79, 1.05)	0.14
**Hepatitides**	Middle-aged adults	0.89 (0.79, 1.01)	0.088
**Hepatitides**	Older adults	1.01 (0.89, 1.14)	0.87
**Inf. Encephalitides**	Children	0.83 (0.56, 1.25)	0.54
**Inf. Encephalitides**	Young adults	1.04 (0.86, 1.26)	0.59
**Inf. Encephalitides**	Middle-aged adults	1.06 (0.89, 1.26)	0.54
**Inf. Encephalitides**	Older adults	1.09 (0.89, 1.32)	0.54
**Meningitides**	Children	1.22 (0.85, 1.74)	0.34
**Meningitides**	Young adults	1.07 (0.87, 1.31)	0.43
**Meningitides**	Middle-aged adults	0.94 (0.78, 1.13)	0.43
**Meningitides**	Older adults	0.82 (0.67, 1.00)	0.057
**RTI**	Children	1.10 (0.81, 1.48)	0.76
**RTI**	Young adults	0.97 (0.84, 1.11)	0.76
**RTI**	Middle-aged adults	0.95 (0.84, 1.08)	0.76
**RTI**	Older adults	0.99 (0.87, 1.12)	0.81

© 2026 Taquet M et al. *JAMA Psychiatry*.

**Skin Infections**	Children	1.14 (0.84, 1.55)	0.55
**Skin Infections**	Young adults	0.90 (0.79, 1.03)	0.21
**Skin Infections**	Middle-aged adults	0.97 (0.86, 1.09)	0.7
**Skin Infections**	Older adults	1.00 (0.89, 1.13)	0.97
**STI**	Children	1.15 (0.80, 1.66)	0.59
**STI**	Young adults	0.95 (0.82, 1.11)	0.59
**STI**	Middle-aged adults	1.02 (0.88, 1.18)	0.77
**STI**	Older adults	0.90 (0.76, 1.05)	0.35
**UTI**	Children	1.17 (0.86, 1.59)	0.35
**UTI**	Young adults	0.88 (0.77, 1.00)	0.062
**UTI**	Middle-aged adults	0.95 (0.84, 1.07)	0.35
**UTI**	Older adults	1.02 (0.91, 1.15)	0.65

© 2026 Taquet M et al. *JAMA Psychiatry*.

eTable 8. Moderation of RMTL ratios by age group and outcome (general population).

Outcome	Age group	Difference (95% CI)	P Value
**Average**	Children	1.21 (1.16, 1.26)	8.90E-27
**Average**	Young adults	0.97 (0.95, 0.99)	7.00E-04
**Average**	Middle-aged adults	0.90 (0.88, 0.92)	2.20E-33
**Average**	Older adults	0.95 (0.93, 0.96)	1.30E-11
**Anxiety disorder**	Children	0.91 (0.79, 1.05)	0.12
**Anxiety disorder**	Young adults	0.95 (0.87, 1.04)	0.2
**Anxiety disorder**	Middle-aged adults	1.07 (0.98, 1.17)	0.092
**Anxiety disorder**	Older adults	1.08 (0.98, 1.18)	0.092
**Any first**	Children	0.92 (0.82, 1.04)	0.12
**Any first**	Young adults	0.92 (0.85, 0.99)	0.012
**Any first**	Middle-aged adults	1.00 (0.93, 1.08)	0.91
**Any first**	Older adults	1.18 (1.10, 1.27)	9.40E-08
**Any first or Death**	Children	0.92 (0.82, 1.03)	0.089
**Any first or Death**	Young adults	0.90 (0.83, 0.97)	0.00084
**Any first or Death**	Middle-aged adults	1.02 (0.95, 1.09)	0.5
**Any first or Death**	Older adults	1.19 (1.11, 1.28)	5.00E-10
**Cognitive deficit**	Children	0.76 (0.65, 0.90)	0.00025
**Cognitive deficit**	Young adults	1.01 (0.90, 1.14)	0.83
**Cognitive deficit**	Middle-aged adults	1.14 (1.04, 1.26)	0.0014
**Cognitive deficit**	Older adults	1.13 (1.03, 1.25)	0.0017
**Death**	Children	1.54 (1.09, 2.16)	0.0034
**Death**	Young adults	1.02 (0.85, 1.23)	0.76
**Death**	Middle-aged adults	0.87 (0.76, 1.01)	0.023
**Death**	Older adults	0.73 (0.64, 0.84)	3.40E-08
**Dementia**	Children	0.70 (0.18, 2.64)	0.7
**Dementia**	Young adults	1.31 (0.69, 2.47)	0.7
**Dementia**	Middle-aged adults	1.13 (0.69, 1.86)	0.7
**Dementia**	Older adults	0.97 (0.60, 1.56)	0.86
**Encephalitis**	Children	0.82 (0.49, 1.38)	0.67
**Encephalitis**	Young adults	0.95 (0.61, 1.47)	0.92
**Encephalitis**	Middle-aged adults	1.02 (0.67, 1.54)	0.92
**Encephalitis**	Older adults	1.27 (0.74, 2.17)	0.67

© 2026 Taquet M et al. *JAMA Psychiatry*.

**Encephalitis**	Children	1.01 (0.35, 2.91)	0.98
**Encephalitis**	Young adults	1.12 (0.54, 2.33)	0.98
**Encephalitis**	Middle-aged adults	0.91 (0.49, 1.68)	0.98
**Encephalitis**	Older adults	0.97 (0.48, 1.96)	0.98
**Epilepsy or seizures**	Children	0.79 (0.65, 0.95)	0.007
**Epilepsy or seizures**	Young adults	0.97 (0.82, 1.14)	0.64
**Epilepsy or seizures**	Middle-aged adults	1.14 (0.98, 1.32)	0.04
**Epilepsy or seizures**	Older adults	1.15 (0.98, 1.34)	0.04
**Hospitalisation**	Children	1.26 (1.18, 1.36)	3.30E-15
**Hospitalisation**	Young adults	1.09 (1.04, 1.15)	5.80E-06
**Hospitalisation**	Middle-aged adults	0.85 (0.82, 0.89)	1.80E-20
**Hospitalisation**	Older adults	0.85 (0.81, 0.88)	1.80E-20

© 2026 Taquet M et al. *JAMA Psychiatry.*

eTable 9. Moderation of absolute risks by age group and infection (general population).

Infection	Age group	Difference (95% CI)	P Value
**Average**	Children	-0.29 (-0.42, -0.17)	1.00E-08
**Average**	Young adults	-0.15 (-0.23, -0.065)	9.60E-06
**Average**	Middle-aged adults	0.065 (-0.019, 0.15)	0.053
**Average**	Older adults	0.37 (0.26, 0.49)	6.80E-14
**Bone Infections**	Children	-1.71 (-2.28, -1.14)	4.10E-13
**Bone Infections**	Young adults	-0.63 (-0.96, -0.30)	3.10E-06
**Bone Infections**	Middle-aged adults	0.60 (0.28, 0.92)	3.10E-06
**Bone Infections**	Older adults	1.73 (1.39, 2.07)	1.10E-32
**Cardiac Infections**	Children	-0.93 (-3.11, 1.25)	0.3
**Cardiac Infections**	Young adults	-0.70 (-1.54, 0.13)	0.072
**Cardiac Infections**	Middle-aged adults	0.33 (-0.47, 1.14)	0.3
**Cardiac Infections**	Older adults	1.30 (0.49, 2.12)	0.00029
**GI Infections**	Children	-5.85 (-14.55, 2.86)	0.13
**GI Infections**	Young adults	8.38 (2.22, 14.54)	0.0028
**GI Infections**	Middle-aged adults	-3.93 (-9.87, 2.01)	0.13
**GI Infections**	Older adults	1.40 (-4.88, 7.67)	0.58
**Hepatitides**	Children	-1.62 (-1.85, -1.39)	8.70E-55
**Hepatitides**	Young adults	-0.71 (-0.97, -0.45)	3.20E-11
**Hepatitides**	Middle-aged adults	0.64 (0.36, 0.92)	1.40E-08
**Hepatitides**	Older adults	1.69 (1.43, 1.94)	4.10E-49
**Inf. Encephalitides**	Children	-1.48 (-9.14, 6.18)	0.63
**Inf. Encephalitides**	Young adults	-0.70 (-3.32, 1.92)	0.63
**Inf. Encephalitides**	Middle-aged adults	0.55 (-2.07, 3.17)	0.63
**Inf. Encephalitides**	Older adults	1.63 (-1.18, 4.45)	0.59
**Meningitides**	Children	-2.15 (-3.49, -0.82)	0.00023
**Meningitides**	Young adults	-1.00 (-2.34, 0.35)	0.085
**Meningitides**	Middle-aged adults	0.38 (-1.14, 1.90)	0.54
**Meningitides**	Older adults	2.77 (-0.045, 5.59)	0.028
**RTI**	Children	-1.77 (-1.97, -1.57)	1.20E-78
**RTI**	Young adults	-0.59 (-0.87, -0.30)	3.00E-07
**RTI**	Middle-aged adults	0.69 (0.41, 0.96)	7.10E-10

© 2026 Taquet M et al. *JAMA Psychiatry*.

**RTI**	Older adults	1.67 (1.42, 1.93)	1.60E-49
**Skin Infections**	Children	-1.60 (-1.79, -1.41)	2.20E-72
**Skin Infections**	Young adults	-0.71 (-0.92, -0.49)	3.80E-15
**Skin Infections**	Middle-aged adults	0.57 (0.35, 0.79)	2.20E-10
**Skin Infections**	Older adults	1.73 (1.50, 1.96)	9.80E-64
**STI**	Children	-2.08 (-3.56, -0.60)	0.00096
**STI**	Young adults	-0.58 (-1.19, 0.023)	0.016
**STI**	Middle-aged adults	0.81 (0.18, 1.44)	0.0019
**STI**	Older adults	1.86 (0.84, 2.87)	2.20E-05
**UTI**	Children	-1.64 (-1.85, -1.43)	1.20E-65
**UTI**	Young adults	-0.72 (-0.95, -0.49)	1.90E-14
**UTI**	Middle-aged adults	0.68 (0.45, 0.92)	6.50E-13
**UTI**	Older adults	1.67 (1.45, 1.90)	1.40E-60

The ‘difference’ represents the moderation of the absolute risk difference by the age group, in %. For instance, in children, the absolute risk difference of outcomes after bone infections (compared to general population) were on average 1.71% lower than in the average across age groups.

eTable 10. Moderation of absolute risks by age group and outcome (general population).

Outcome	Age group	Difference (95% CI)	P Value
**Average**	Children	-0.29 (-0.42, -0.17)	1.00E-08
**Average**	Young adults	-0.15 (-0.23, -0.065)	9.60E-06
**Average**	Middle-aged adults	0.065 (-0.019, 0.15)	0.053
**Average**	Older adults	0.37 (0.26, 0.49)	6.80E-14
**Anxiety disorder**	Children	-2.85 (-4.34, -1.37)	7.70E-06
**Anxiety disorder**	Young adults	0.95 (-0.57, 2.47)	0.12
**Anxiety disorder**	Middle-aged adults	1.01 (-0.36, 2.39)	0.12
**Anxiety disorder**	Older adults	0.89 (-0.44, 2.23)	0.12
**Any first**	Children	-5.39 (-7.27, -3.51)	8.30E-12
**Any first**	Young adults	-1.03 (-3.12, 1.05)	0.29
**Any first**	Middle-aged adults	0.84 (-1.28, 2.96)	0.32
**Any first**	Older adults	5.58 (3.44, 7.72)	2.90E-10
**Any first or Death**	Children	-7.27 (-9.19, -5.36)	1.80E-19
**Any first or Death**	Young adults	-2.46 (-4.59, -0.34)	0.0051
**Any first or Death**	Middle-aged adults	1.42 (-0.77, 3.62)	0.11
**Any first or Death**	Older adults	8.31 (6.10, 10.52)	1.80E-19
**Cognitive deficit**	Children	-5.27 (-6.71, -3.84)	1.20E-18
**Cognitive deficit**	Young adults	-1.57 (-2.71, -0.43)	0.00079
**Cognitive deficit**	Middle-aged adults	1.20 (-0.038, 2.43)	0.015
**Cognitive deficit**	Older adults	5.64 (4.21, 7.08)	1.10E-20
**Death**	Children	-6.54 (-7.88, -5.21)	7.90E-31
**Death**	Young adults	-3.33 (-4.29, -2.37)	5.90E-17
**Death**	Middle-aged adults	1.68 (0.57, 2.78)	0.00017
**Death**	Older adults	8.20 (6.96, 9.44)	2.10E-50
**Dementia**	Children	-1.40 (-2.62, -0.18)	0.0082
**Dementia**	Young adults	0.038 (-0.69, 0.77)	0.9
**Dementia**	Middle-aged adults	-0.77 (-1.51, -0.03)	0.013
**Dementia**	Older adults	2.13 (1.17, 3.09)	1.60E-07
**Encephalitis**	Children	-0.44 (-1.66, 0.79)	0.5
**Encephalitis**	Young adults	0.93 (0.20, 1.65)	0.0055
**Encephalitis**	Middle-aged adults	-0.45 (-1.16, 0.25)	0.21

© 2026 Taquet M et al. *JAMA Psychiatry.*

**Encephalitis**	Older adults	-0.034 (-0.81, 0.74)	0.91
**Encephalitis**	Children	-0.46 (-1.67, 0.75)	0.45
**Encephalitis**	Young adults	0.91 (0.20, 1.61)	0.0053
**Encephalitis**	Middle-aged adults	-0.46 (-1.15, 0.23)	0.2
**Encephalitis**	Older adults	0.0092 (-0.76, 0.78)	0.98
**Epilepsy or seizures**	Children	-0.79 (-2.15, 0.56)	0.28
**Epilepsy or seizures**	Young adults	0.70 (-0.21, 1.61)	0.21
**Epilepsy or seizures**	Middle-aged adults	-0.15 (-1.03, 0.74)	0.68
**Epilepsy or seizures**	Older adults	0.24 (-0.70, 1.17)	0.68
**Hospitalisation**	Children	0.96 (-1.30, 3.23)	0.38
**Hospitalisation**	Young adults	0.87 (-0.83, 2.57)	0.38
**Hospitalisation**	Middle-aged adults	-1.38 (-2.93, 0.17)	0.1
**Hospitalisation**	Older adults	-0.45 (-2.01, 1.10)	0.46

The ‘difference’ represents the moderation of the absolute risk difference by the age group, in %. For instance, in children, the absolute risk difference of cognitive deficits (averaged over all infections) was 5.27% lower than in the average across age groups and infections.

© 2026 Taquet M et al. *JAMA Psychiatry*.
