# Supplementary material for: Mapping 2-Year Psychiatric and Neurologic Risks After Infections Across Body Systems and Age Groups
Source: JAMA Psychiatry. 2026 Jul 15:e261904. Online ahead of print. doi: 10.1001/jamapsychiatry.2026.1904 (PMC13373730; doi:10.1001/jamapsychiatry.2026.1904)
Supplement: Supplement 2. — Data Sharing Statement. [file jamapsychiatry-e261904-s002.pdf]

Data Sharing Statement

Taquet. Mapping 2-Year Psychiatric and Neurologic Risks After Infections Across Body Systems and Age Groups. *JAMA Psychiatry*. Published July 15, 2026.
doi:10.1001/jamapsychiatry.2026.1904

Data
Data available: Yes
Data types: Other (please specify)
Additional Information: Aggregate data for each comparison (i.e. summary statistics for each comparison, each outcome, and each age group) will be freely accessible
How to access data: The data will be stored on Open Science Framework (URL provided upon acceptance of the paper).
When available: With publication

Supporting Documents
Document types: Statistical/analytic code
How to access documents: The code will be available via the same Open Science Framework repository as the data (URL provided upon acceptance of the paper).
When available: With publication

Additional Information
Who can access the data: Anyone with the URL
Types of analyses: Any
Mechanisms of data availability: Via an open access online repository
